# Supplementary material for: Operando-electrified solvay process
Source: Nat Commun. 2025 Oct 14;16:9128. doi: 10.1038/s41467-025-63539-3 (PMC12521556; doi:10.1038/s41467-025-63539-3)
Supplement: Supplementary file 1 — Supporting Information [file 41467_2025_63539_MOESM1_ESM.pdf]

Supplementary Note 1:

Derivation of quantification for HCO_3^- product

According to the dissociation equilibria of carbonic acid before and at the end points of the titration, we have

$$\frac{[HCO_3^-]_i[H]_i}{[H_2CO_3]_i} = K,\ i = 1,2,3 \tag{1}$$

Where $[HCO_3^-]_i$, $[H_2CO_3]$ and $[H]_i$ are concentrations of bicarbonate, carbonic acid and hydrogen ions before the titration (i = 1), at the first endpoint (i = 2) and at the second endpoint (i = 3), respectively.

Commonly, carbon dioxide emissions from the sample during titration are negligible, so the sum of bicarbonate and carbonic acid in a sample is constant. Therefore,

$$[HCO_3^-]_i + [H_2CO_3]_i = C,\ i = 1,2,3 \tag{2}$$

where C is the sum of concentrations of carbonic acid and bicarbonate in the sample. In addition, the mass change between the acid consumption during the titration (a_1) and the corresponding conversion of the relevant chemical species in the sample (a_2) can be written as:

$$a_1 = [HCO_3^-]_1 - [HCO_3^-]_2 \tag{3}$$

$$a_2 = [HCO_3^-]_1 - [HCO_3^-]_3 \tag{4}$$

Next, we substitute equations (1) to (2) with appropriate rearrangement:

$$[HCO_3^-]_i = \frac{K + [H]_1}{K + [H]_i}[HCO_3^-]_1,\ i = 2,3 \tag{5}$$

And we substitute equations (5) into (3) and (4) with appropriate rearrangement:

$$a_1 = \frac{[HCO_3^-]_1([H]_2 - [H]_1)}{[H]_2 + K} \tag{6}$$

$$a_2 = \frac{[HCO_3^-]_1([H]_3 - [H]_1)}{[H]_3 + K} \quad (7)$$

The $[HCO_3^-]_1$ can be replaced with $[HCO_3^-]_{(a_1)}$ and $[HCO_3^-]_{(a_2)}$ in equation (6) and (7)， respectively. The total bicarbonate concentration is the summation of $[HCO_3^-]$ from equation (6) and (7).

Supplementary Note 2:

Economic calculation

1. Electrochemical method in this work

To clearly prove the economic viability of our $NaHCO_3$ production system, we conducted a comprehensive economic assessment. To better reflect the actual industrial situation, equipment investment of electrolyzer and supporting facilities, electricity cost, electrolyte and membrane replacement cost, and maintenance cost are included in the calculation scope. Furthermore, separation costs, which are of significant importance, have been included in the evaluation scope. For convenient considerations, the cost calculation parameters of the separation system are borrowed from the report that has been reported literature. On the basis of the US Department of Energy's recently announced utility-scale solar 2025 cost target,[1] we estimated a cost of renewable electricity of 0.03 USD/kWh and 90% power conversion efficiency.[2] The specific calculation method is as follows.

The main operating conditions are as follows: 10 tons/day $NaHCO_3$ production capacity, 350 days/year of operation time, 240 mA cm^{-2} current density at 2.42V (in unit module cell at 25 cm^2). Other operation parameters are as follows:

According to the production capacity and experimental conversion efficiency (6.6) of reaction process. the required total current is calculated as:

$$total\ current\ =10000\frac{kg}{day}*\frac{day}{24h}*\frac{h}{3600s}*\frac{kmol}{84kg}*8e^{-}*96485\frac{c}{mol}*\frac{1}{6.6} \tag{8}$$
$$=161143.5A$$

The electrolyzer area needed is the total current divided by the current density:

$$total\ electrolyzer\ area=\frac{161143.5A}{0.24\frac{A}{cm^2}}\frac{m^2}{10000cm^2}=67.1\,m^2 \tag{9}$$

The power needed is given from P = UI, the cell voltage is 2.42 V for 240 mA cm^{-2}:

$$power=voltage*161143.5A*\frac{kW}{1000W}=2.42*161143.5/1000=390kW \tag{10}$$

The water flow rate for the OER reaction is:

$$H_2O\ flow\,rate=161143.5A*\frac{1}{4e^{-}*96485\frac{C}{mol}}*\frac{0.018kg}{mol}*\frac{86400s}{day}$$
$$=649.4\frac{kg}{day} \tag{11}$$

Given the Na^{+} utilization of 89%, the $NaNO_3$ flow rate in cathode is:

$$NaNO_3\ flow\,rate=161143.5A*\frac{1}{8e^{-}*96485\frac{C}{mol}}*\frac{0.085kg}{mol}*\frac{86400s}{day}*\frac{1}{0.89}$$
$$=1722.7\frac{kg}{day}$$

(12)

The CO_2 flow rate needed in cathode is:

$$CO_2\ flow\,rate==10000\frac{kg}{day}*\frac{44}{84}=5238.1\frac{kg}{day} \tag{13}$$

1.1. Raw material cost

For the raw chemicals cost, we account for the cost from the consumed H_2O for OER reaction. The water price is estimated as \$ 0.003 per liter:

$$H_2O\cos t=\$0.003*649.4\frac{kg}{day}=\$1.9/day \tag{14}$$

We calculate the cost of consumed $NaNO_3$ in cathodic reaction. The $NaNO_3$ price is estimated as \$ 280 per ton:[3]

$$cathode\ NaNO_3\ cost = \frac{\$280}{ton} * 1722.7\frac{kg}{day} * \frac{ton}{1000kg} = \$482.4 / day \quad (15)$$

Consider 1% loss for the $NaNO_3$ in anode system:

$$anode\ NaNO_3\ cost = \frac{\$280}{ton} * 649.4\frac{kg}{day} * \frac{ton}{1000kg} * 17\% * 1\% = \$0.3 / day \quad (16)$$

The consumed CO_2 is considered. The CO_2 price is estimated as \$ 0.056 per kg:[4]

$$CO_2\ cost = \$0.056 * 5238.1\frac{kg}{day} = \$293.3 / day \quad (17)$$

1.2. Electrolyzer cost

Since cathode catalysts are non-precious metal-based materials, and their cost is negligible compared with that of noble metal Pt and Ir. Here, we use 500 USD/kW as the stack cost.[5] The reference electrolyzer operates at 1.8 V and 2 A cm^{-2} and the installation factor is 1.12. The balance of plant (BOP) cost is assumed to be \$4914 m^{-2},[6] and these values were derived from the H_2A model. We assume that the capital cost of the separation process accounts for 20% of the raw material cost.[7]

$$\begin{aligned} cost &= reported\ cost(\$/kW) * reported\ power\ density(kW/m^2) \\ &= 500\frac{\$}{kW} * 2\frac{A}{cm^2} * 1.8V * \frac{10000cm^2}{m^2} * \frac{kW}{1000W} = \$18000/m^2 \end{aligned} \quad (18)$$

At 240 mA cm^{-2}, the total electrolyzer area is 67.1 m^2 and the total cost is:

$$\begin{aligned} electrolyzer\ cost &= area * cost(\$/m^2) * installation\ factor \\ &= 67.1m^2 * \$18000/m^2 * 1.12 = \$1352736 \end{aligned} \quad (19)$$

Herein, the capital recovery factor (CRF) is based on a discount rate (denoted as i; we use 5% for all the CRF calculations) and the material lifetime (40 years).

$$CRF_{electrolyzer} = \frac{i(i+1)^{year}}{(1+i)^{year} - 1} = \frac{0.05 * 1.05^{40}}{1.05^{40} - 1} = 0.058 \quad (20)$$

$$electrolyzer\ \cos t_{\ per\ ton\ NaHCO_3} = \$1352736 * \frac{0.058}{350day} = \$224.2 / day \tag{21}$$

the total BOP capital cost is:

$$\begin{aligned} &BOP\ capital\ \cos t = area * reference\ \cos t * (installation\ \ factor) \\ &= 67.1m^2 * \$4914 / m^2 * 1.12 = \$369296.9 \end{aligned} \tag{22}$$

Herein, the CRF is based on a discount rate (denoted as i; we use 5% for all the CRF calculations) and the material lifetime (40 years).

$$BOP\ capital\ \cos t_{\ per\ ton\ NaHCO_3} = \$369296.9 * \frac{0.058}{350day} = \$61.2 / day \tag{23}$$

$$overall\ capital\ \cos t_{\ per\ ton\ NaHCO_3} = \$224.2 / day + \$61.2 / day = \$285.4 / day \tag{24}$$

1.3. Electricity cost

The electricity cost is calculated from the power requirement and the price of electricity, assuming the electricity price is 5 cent kWh^{-1}:

$$electricity\ \cos t_{\ per\ ton\ NaHCO_3} = 390kW * 24hr / day * \frac{\$0.05}{kWh} = \$468 / day \tag{25}$$

1.4. Stack replacement cost

For the stack replacement cost, assuming 30% of the uninstall cost for 10 years.

$$stack\ \ replacement\ \ \cos t = \$1352736 * \frac{0.30}{10year * 350day} = \$115.9 / day \tag{26}$$

1.5. Separation cost

We assume that the capital cost of the separation accounts for 20% of the raw material cost.

$$separation\ \cos t = (\$429.3 + \$0.3) * 0.2 = \$85.9 / day \tag{27}$$

1.6. Other cost

Other operational costs, such as labor and maintenance, are assumed to be 2.5% of the electrolyzer cost per ton of $NaHCO_3$:

$$other\cos t_{\ per\ ton\ NaHCO_3} = \$1352736 * \frac{0.025}{350day} = \$96.6/day \quad (28)$$

Thus, the overall cost to generate on ton of $NaHCO_3$ per day is:

$$overall\cos t_{\ per\ ton\ NaHCO_3} = \$285.4 + \$468 + \$1.9 + \$482.4 + \$0.3 + \$293.3 \\ +\$115.9 + \$85.9 + \$96.6 = \$1829.7/day \quad (29)$$

2. Traditional Solvay process

In order to gain a more comprehensive understanding of the relative merits of our electrochemical production system, we also undertook an evaluation of the economic costs associated with the traditional Solvay process. Once more, we assumed a production of 10000 kg of $NaHCO_3$ per day. The demand for raw salt was set to 153% based on the literature.[8] The primary costs comprise raw material costs, equipment and replacement costs, energy consumption costs, product separation costs, and other costs. We convert these costs into a proportion of the cost of raw salt.

2.1. Raw material cost

The raw materials required for traditional production are raw salt (NaCl), NH_3, quicklime (CaO), CO_2, and water. The requirements for NH_3, CaO and CO_2 are based on stoichiometric ratios of $NaHCO_3$.

The NaCl price is estimated as \$ 70 per ton [9]:

$$NaCl\cos t = \$70/ton * 10000\frac{kg}{day} * \frac{ton}{1000kg} * 1.53 = \$1071/day \quad (30)$$

The NH_3 price is estimated as \$ 450 per ton[10]:

$$NH_3\cos t = \frac{\$450}{ton} * 10000\frac{kg}{day} * 1.53 * \frac{17}{59} * \frac{ton}{1000kg} = \$1983/day \quad (31)$$

The CO_2 price is estimated as \$ 0.056 per kg[4]:

$$CO_2\cos t = \$0.056 * 10000\frac{kg}{day} * \frac{44}{84} = \$293.3/day \quad (32)$$

The CaO price is estimated as \$ 30 per ton[11]:

$$CaO\cos t = \frac{\$30}{ton} * 10000\frac{kg}{day} * 1.53 * \frac{17}{59} * \frac{1}{2} * \frac{ton}{1000kg} = \$66.1/day \quad (33)$$

We assume that the water needed is 5% of the NaCl consumption:

$$H_2O\cos t = \$0.003/kg * 10000\frac{kg}{day} * 1.53 * 5\% = \$2.3/day \quad (34)$$

2.2. Equipment cost

Conventional production processes require similarly high equipment requirements, and here we have assumed costs consistent with our electrochemical process.

$$overall\ capital\cos t_{\ per\ ton\ NaHCO_3} = \$285.4/day \quad (35)$$

2.3. Equipment replacement cost

Here we assume that the cost of equipment replacement is 20% of the overall cost of the equipment:

$$equipment\ replacement\cos t_{\ per\ ton\ NaHCO_3} = \$285.4/day * 0.2 = \$57/day \quad (36)$$

2.4. Energy consumption cost

Here, we assume that the energy consumption cost is 20% of the cost of raw salt:

$$energy\ consumption\cos t = \$1071 * 0.2 = \$214.2/day \quad (37)$$

2.5. Separation cost

We assume that the capital cost of the separation accounts for 10% of the raw material cost.

$$separation\cos t = \$1071 * 0.1 = \$107.1/day \quad (38)$$

2.6. Other cost

Other operational costs, such as labor and maintenance, are assumed to be 2.5% of the electrolyzer cost per ton of $NaHCO_3$:

$$other\cos t_{\ per\ ton\ NaHCO_3} = \$1071 * 0.025 = \$26.7/day \quad (39)$$

Thus, the overall cost to generate 1-ton $NaHCO_3$/day is:

$$overall\ \cos t_{\ per\ ton\ NaHCO_3} = \$1071 + \$1983 + \$293.3 + \$66.1 + \$2.3 + \$285.4 + \$57 + \$214.2 + \$107.1 + \$26.7 = \$4106.1 / day \quad (40)$$

Supplementary Figures

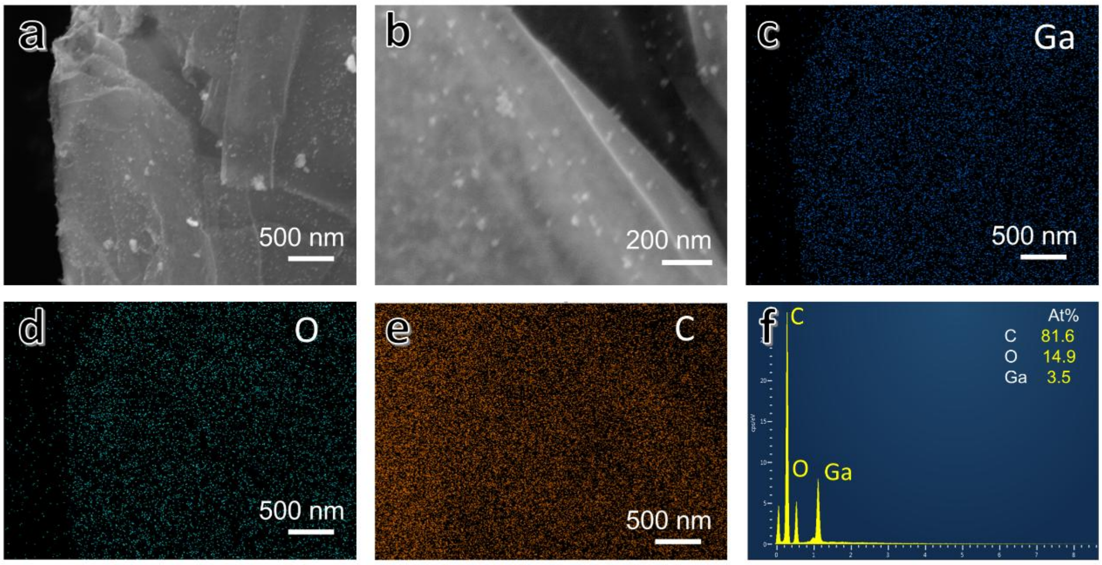

Supplementary Fig. 1. a–**b**, SEM images of GaOOH/rGO. **c**–**e**, EDS mapping images of GaOOH/rGO. **f**, element percentages of GaOOH/rGO.

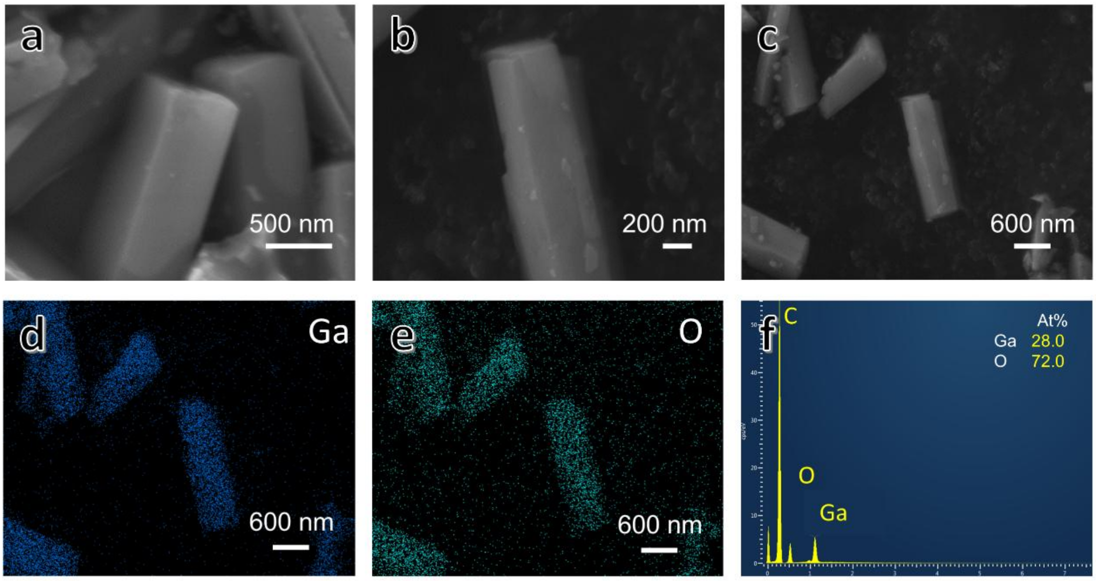

Supplementary Fig. 2. **a**–**c**, SEM images of GaOOH. **d**–**e**, EDS mapping images of GaOOH. **f,** element percentages of GaOOH.

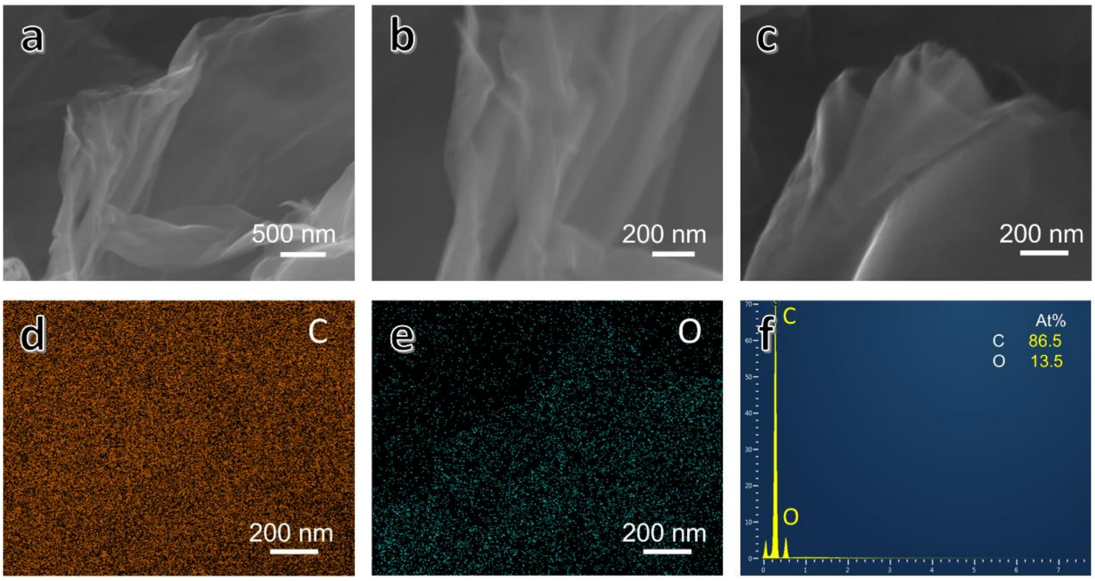

Supplementary Fig. 3. a–**c**, SEM images of rGO. **d**–**e,** EDS mapping images of rGO. **f,** element percentages of rGO.

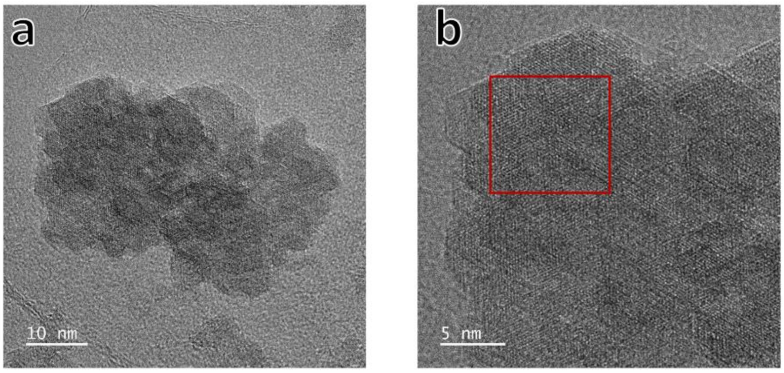

Supplementary Fig. 4. **a–b,** TEM images of GaOOH/rGO, with the marked area corresponding to the Fig. 2a.

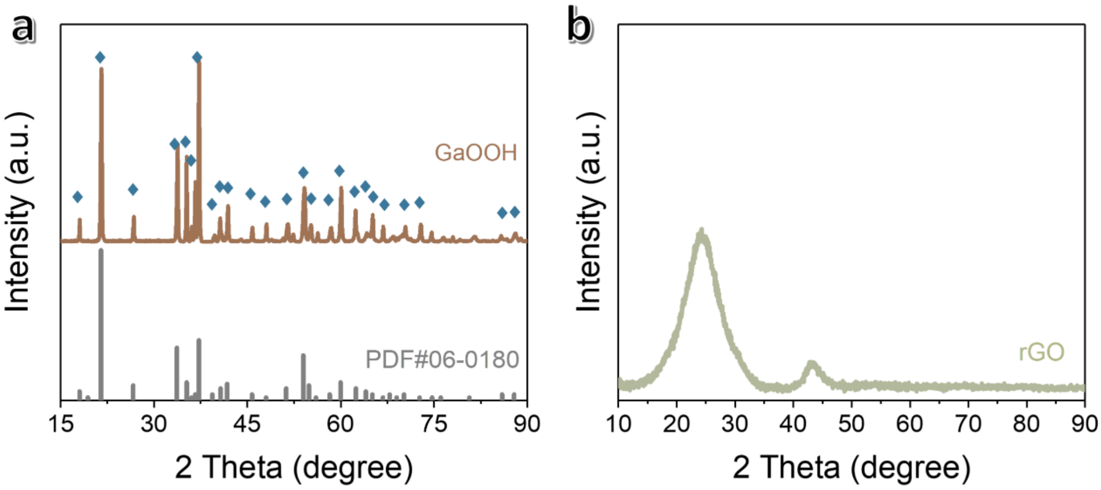

Supplementary Fig. 5. **a,** XRD pattern of GaOOH. **b**, XRD pattern of rGO. Source data for **a-b** are provided as a Source Data file.

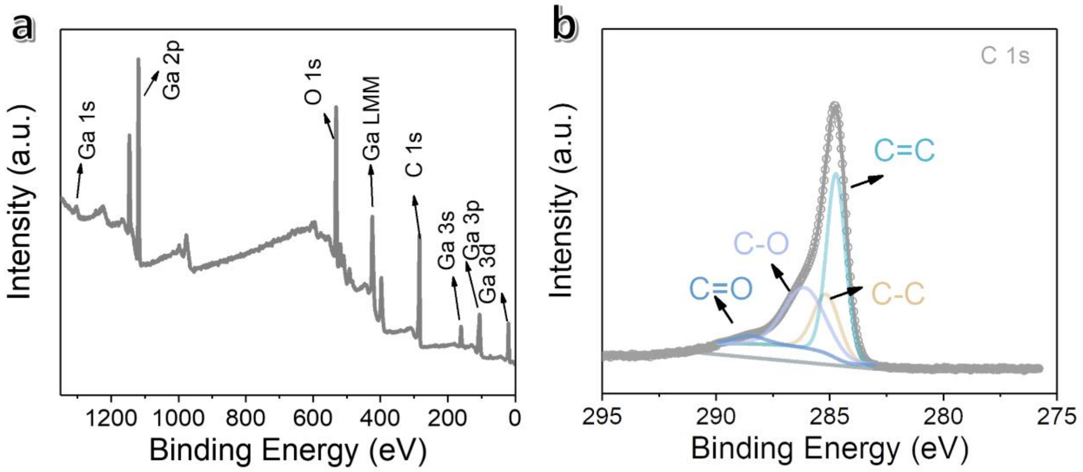

Supplementary Fig. 6. a, XPS survey of GaOOH/rGO. **b**, XPS C *1s* peaks of GaOOH/rGO. Source data for **a-b** are provided as a Source Data file.

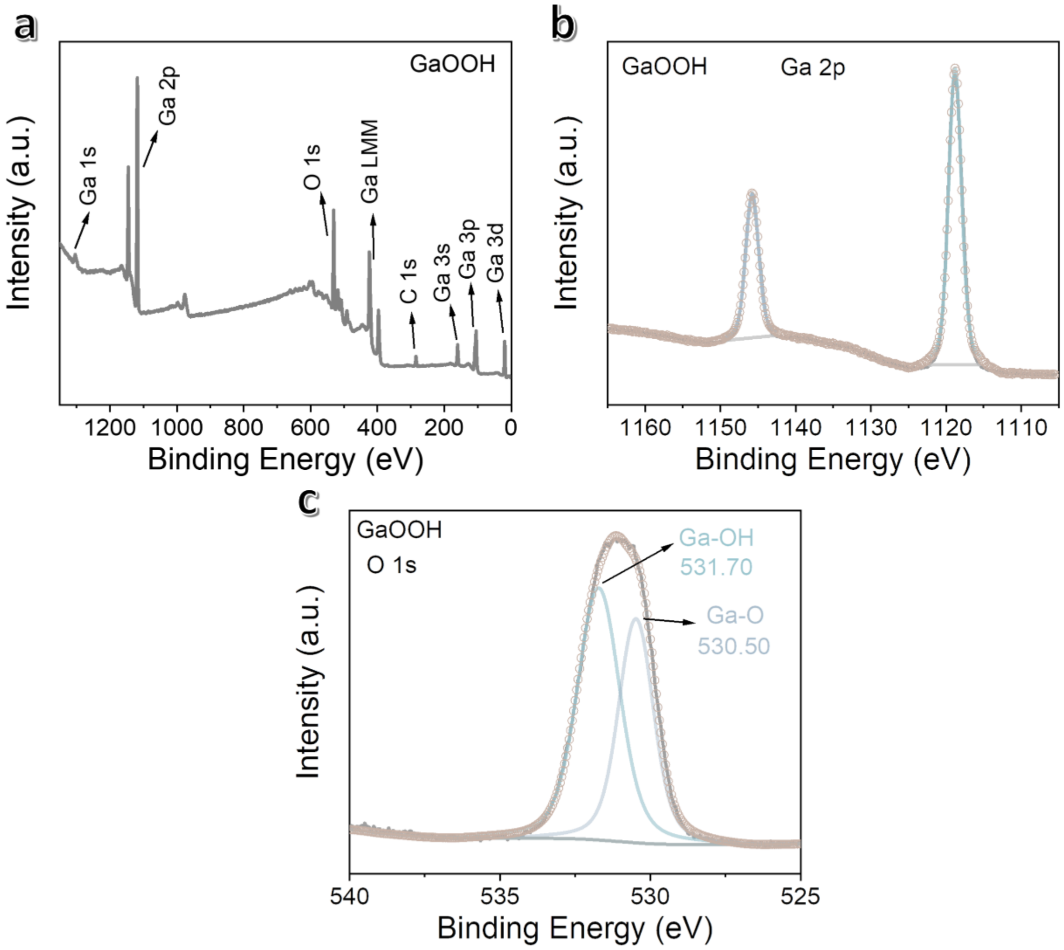

Supplementary Fig. 7. a, XPS survey of GaOOH. **b**, XPS Ga *2p* peaks of GaOOH. **c**, XPS O *1s* peaks of GaOOH. Source data for **a-c** are provided as a Source Data file.

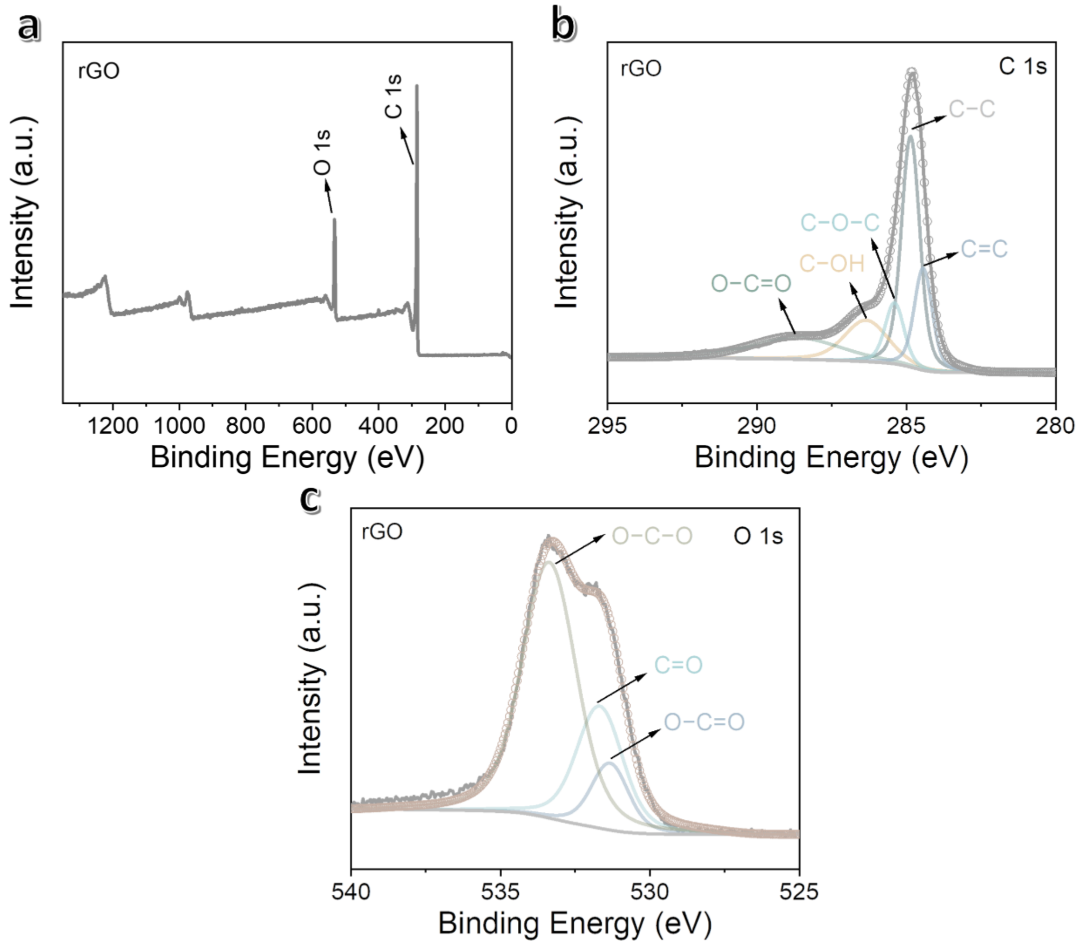

Supplementary Fig. 8. a, XPS survey of rGO. **b**, XPS C *1s* peaks of rGO. **c**, XPS O *1s* peaks of rGO. Source data for **a-c** are provided as a Source Data file.

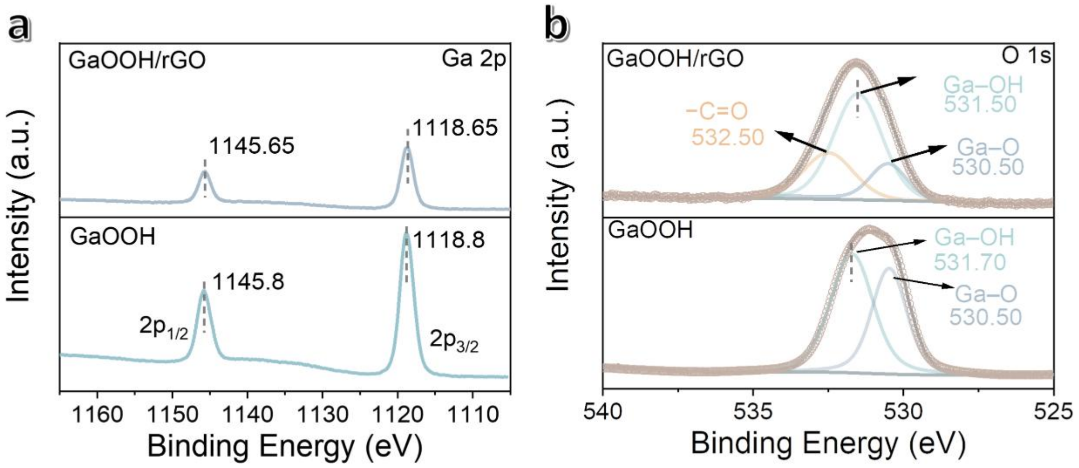

Supplementary Fig. 9. a, The comparison of Ga *2p* peaks for GaOOH/rGO and GaOOH. **b**, The comparison of O *1s* peaks for GaOOH/rGO and GaOOH. The Ga *2p* peak in GaOOH/rGO has shifted downward by 0.15 eV relative to pure GaOOH, while the O *1s* peak (Ga–OH) has shifted by 0.2 eV. These changes are attributed to electron transfer from electron-rich rGO to GaOOH, leading to an increase in outer-shell electrons of Ga and O atoms and corresponding decrease in binding energies. Source data for **a-b** are provided as a Source Data file.

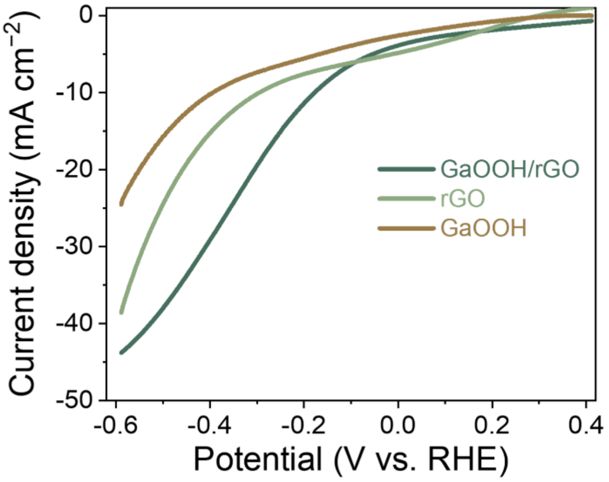

Supplementary Fig. 10. The linear sweep voltammetry curves of GaOOH/rGO, GaOOH and rGO catalysts. Source data is provided as a Source Data file.

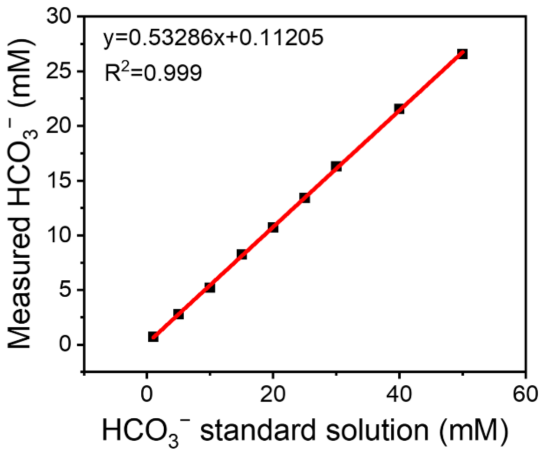

Supplementary Fig. 11. The standard curve of titration method for determining the concentration of bicarbonate. Source data is provided as a Source Data file.

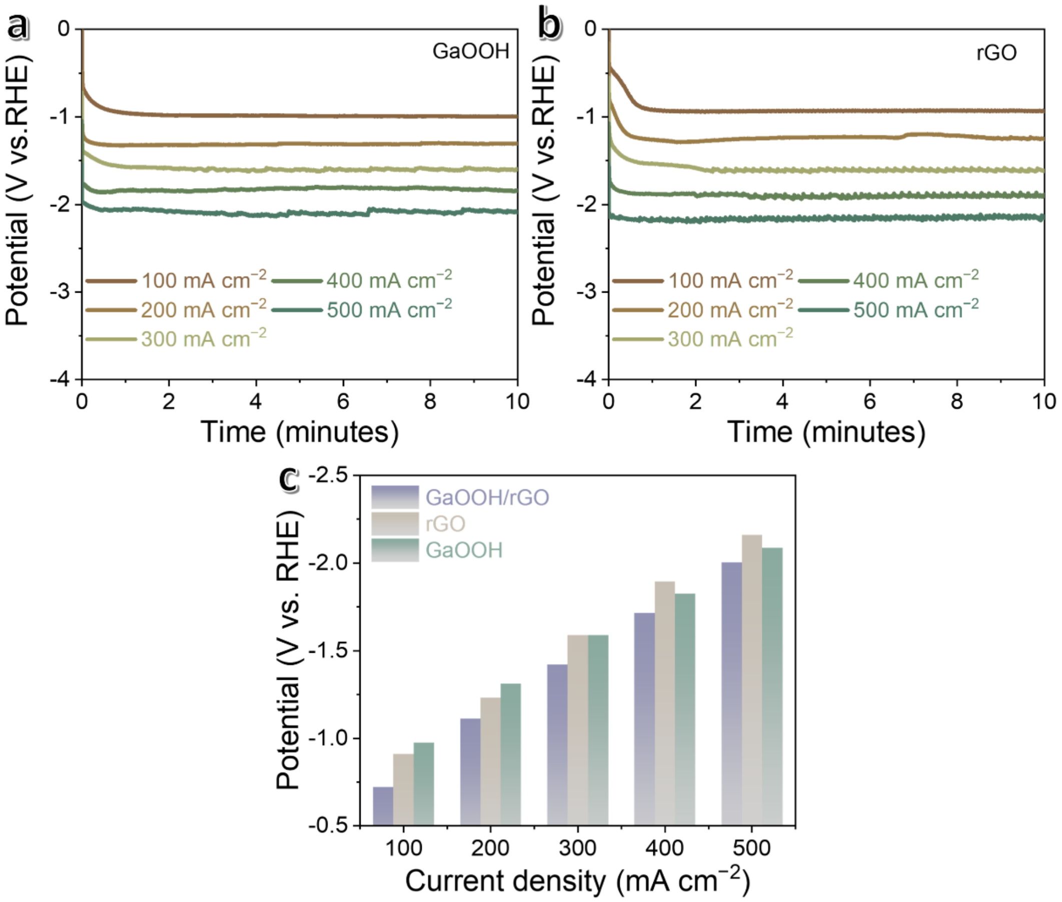

Supplementary Fig. 12. **a,** The chronopotentiometry tests of GaOOH catalyst at different current densities in 2M $NaNO_3$ aqueous solution with CO_2 pumping. **b,** The chronopotentiometry tests of rGO catalyst at different current densities in 2 M $NaNO_3$ aqueous solution with CO_2 pumping. **c**, The average potentials of GaOOH/rGO, GaOOH and rGO catalysts at different current densities in chronopotentiometry tests. Source data for **a-c** are provided as a Source Data file.

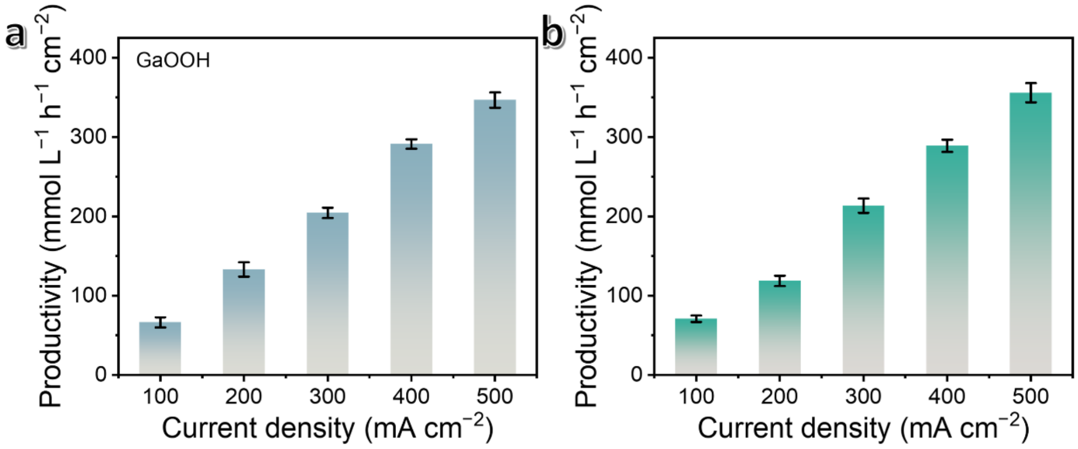

Supplementary Fig. 13. The $NaHCO_3$ productivity in chronopotentiometry tests. **a**, GaOOH catalyst in 2 M $NaNO_3$ aqueous solution with CO_2 pumping. **b**, rGO catalyst in 2 M $NaNO_3$ aqueous solution with CO_2 pumping. The error bars correspond to the standard deviation. Source data for **a-b** are provided as a Source Data file.

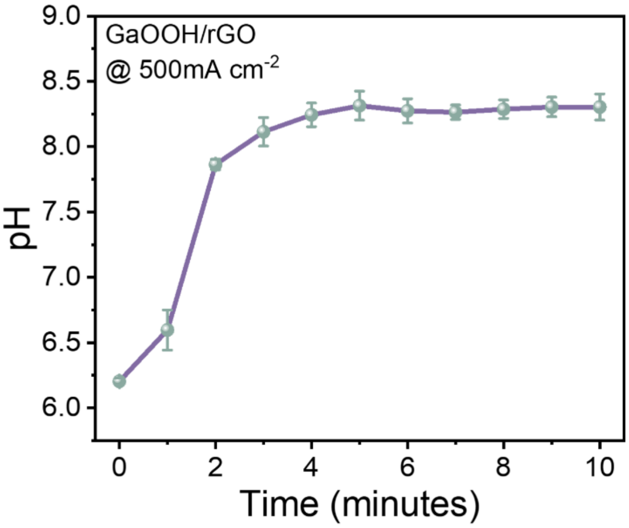

Supplementary Fig. 14. The pH values for GaOOH/rGO system at 500 mA cm^{-2} in a 10-minute test. The error bars correspond to the standard deviation. Source data is provided as a Source Data file.

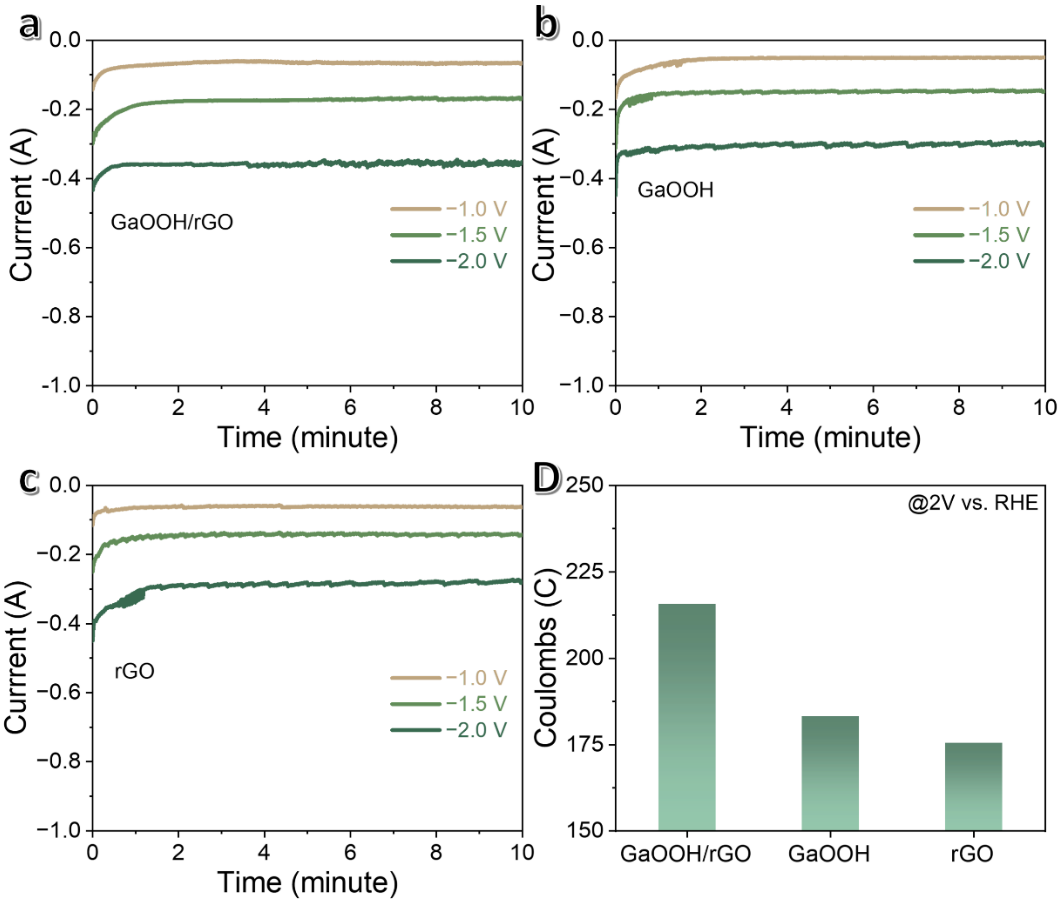

Supplementary Fig. 15. The chronoamperometry tests at different potentials (vs. RHE) in 2 M $NaNO_3$ aqueous solution with CO_2 pumping for **a,** GaOOH/rGO catalyst. **b**, GaOOH catalyst and **c**, rGO catalyst, respectively. **d**, reaction coulombs of GaOOH/rGO, GaOOH and rGO under chronoamperometry tests at 2V vs. RHE. Source data for **a-d** are provided as a Source Data file.

We have found discrepancy between different electrochemical testing techniques, *i.e.*, chronoamperometry (Figures 2d, 2e) and chronopotentiometry (Figure 2f). Generally, chronopotentiometry uses fixed current as controlled variable and potential as response variable. This is different from chronoamperometry with fixed applied potential as controlled variable and current as response variable. Ideally, the results obtained by these two techniques should be the same because of they reflecting the performance of the same electrocatalytic system. Nevertheless, in actual

electrochemical conditions, applying these two techniques can cause different alterations in system parameters, leading to discrepant test results. The system parameters mainly include electrode surface states (active sites, deposition of by-products), mass transfer (reactant supply under high current), and ohmic drop.

Consequently, in our electrochemical system, the amount of reaction transferred coulombs is used as the benchmark to evaluate reaction performance.[12, 13] As shown in Supplementary Fig. 15d, the reaction coulombs of GaOOH/rGO, GaOOH and rGO under chronoamperometry testing at 2V *vs.* RHE are 215.7 C, 183.2 C and 175.5 C, which correspond to yields of 293.0, 228.3 and 218.8 mmol L^{-1} h^{-1} cm^{-2}, respectively (Figure 2f). On the other hand, in chronopotentiometry tests (Figure 2d–e; Supplementary Fig. 12–13), the coulombs transferred at 500 mA cm^{-2} are constant at 300 C, with yields of 374.4, 343.1 and 355.8 mmol L^{-1} h^{-1} cm^{-2}.

Further, we have surveyed literature and found many previous studies with discrepant results in chronoamperometry and chronopotentiometry results, including nitrate reduction reaction,[12] oxygen reduction reaction,[13] organic oxidation reaction,[14, 15] and carbon dioxide reduction reaction.[16] All of the productivities have been evaluated by the amount of reaction transferred coulombs as the benchmark.

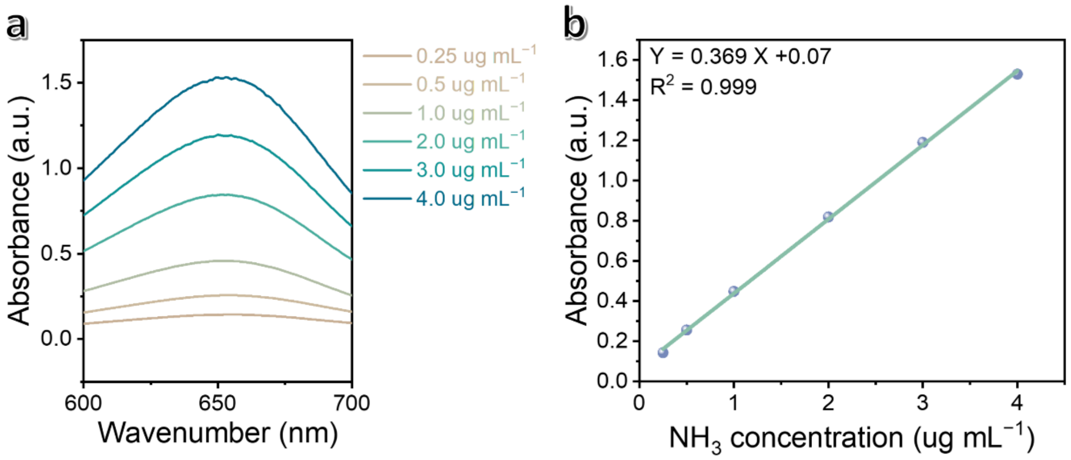

Supplementary Fig. 16. UV-vis calibration curve of NH_3. **a**, the UV-vis spectra of standard NH_4Cl solution with different concentrations. **b**, fitted calibration curve used for estimating NH_3 byproduct. Source data for **a-b** are provided as a Source Data file.

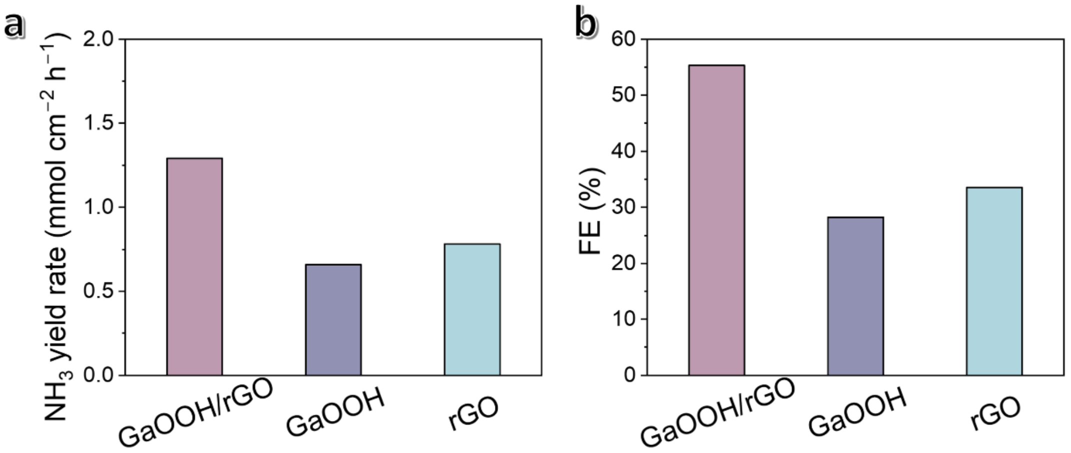

Supplementary Fig. 17. The NH_3 yield rate and Faradaic efficiency for GaOOH/rGO, GaOOH and rGO systems at a current density of 500 mA cm^{-2}. **a**, NH_3 yield rate. **b**, Faradaic efficiency of NH_3 yield. At a current density of 500 mA cm^{-2}, GaOOH/rGO achieved a NH_3 yield rate of 1.29 mmol cm^{-2} h^{-1} and Faradaic efficiency of 55.3%, outperforming the comparison samples of GaOOH (0.66 mmol cm^{-2} h^{-1}, 28.2%) and rGO (0.78 mmol cm^{-2} h^{-1}, 33.5%). Source data for **a-b** are provided as a Source Data file.

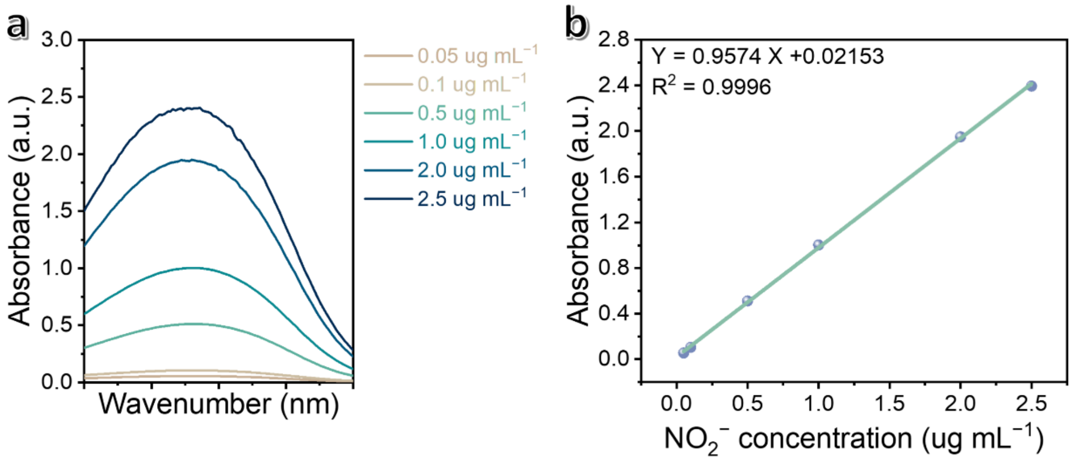

Supplementary Fig. 18. UV-vis calibration curve of NO_2^-. **a**, the UV-vis spectra of standard $NaNO_2$ solution with different concentrations. **b**, fitted calibration curve used for estimating NO_2^- byproduct. Source data for **a-b** are provided as a Source Data file.

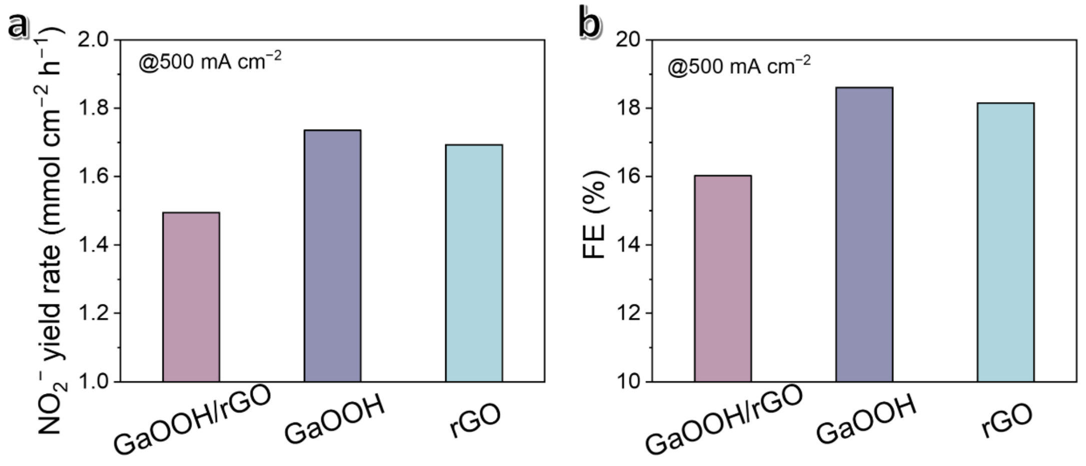

Supplementary Fig. 19. The NO_2^- yield rate and Faradaic efficiency for GaOOH/rGO, GaOOH and rGO samples at a current density of 500 mA cm^{-2}. **a**, yield rate. **b**, Faradaic efficiency. The NO_2^- yield rate of GaOOH/rGO was 1.49 mmol cm^{-2} h^{-1} (Faradaic efficiency of 16.0%), while GaOOH was 1.74 mmol cm^{-2} h^{-1} (Faradaic efficiency of 18.6%) and rGO was 1.69 mmol cm^{-2} h^{-1} (Faradaic efficiency of 18.2%), respectively. Source data for **a-b** are provided as a Source Data file.

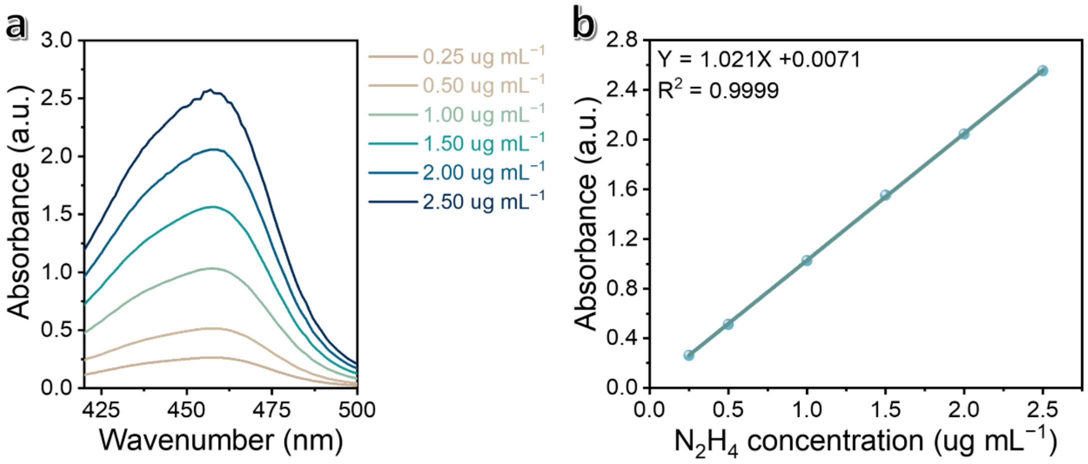

Supplementary Fig. 20. UV-vis calibration curve of N_2H_4. **a**, the UV-vis spectra of standard N_2H_4 solution with different concentrations. **b**, fitted calibration curve used for estimating N_2H_4 byproduct. Source data for **a-b** are provided as a Source Data file.

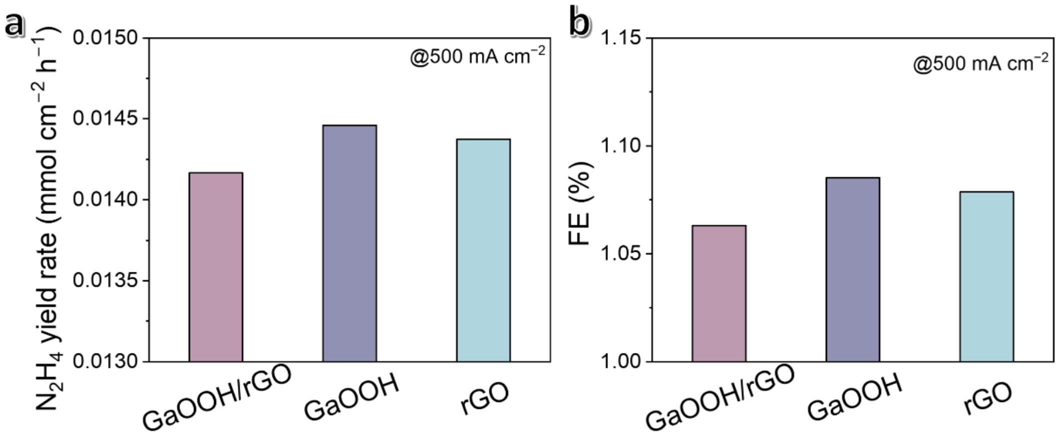

Supplementary Fig. 21. The N_2H_4 yield rate and Faradaic efficiency for GaOOH/rGO, GaOOH and rGO samples at a current density of 500 mA cm^{-2}. **a**, yield rate. **b**, Faradaic efficiency. The N_2H_4 yield rate of GaOOH/rGO was 0.0142 mmol cm^{-2} h^{-1} (Faradaic efficiency of 1.06%), while GaOOH was 0.0145 mmol cm^{-2} h^{-1} (Faradaic efficiency of 1.09%) and rGO was 0.0144 mmol cm^{-2} h^{-1} (Faradaic efficiency of 1.08%), respectively. Source data for **a-b** are provided as a Source Data file.

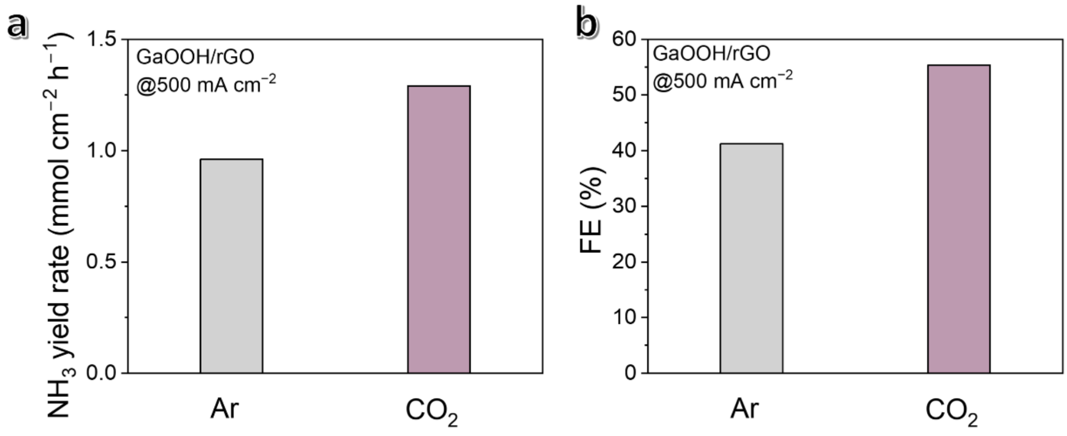

Supplementary Fig. 22. The NH_3 yield rate and Faradaic efficiency for GaOOH/rGO at a current density of 500 mA cm^{-2}, with Ar and CO_2 pumping, respectively. **a**, yield rate. **b**, Faradaic efficiency. Source data for **a-b** are provided as a Source Data file.

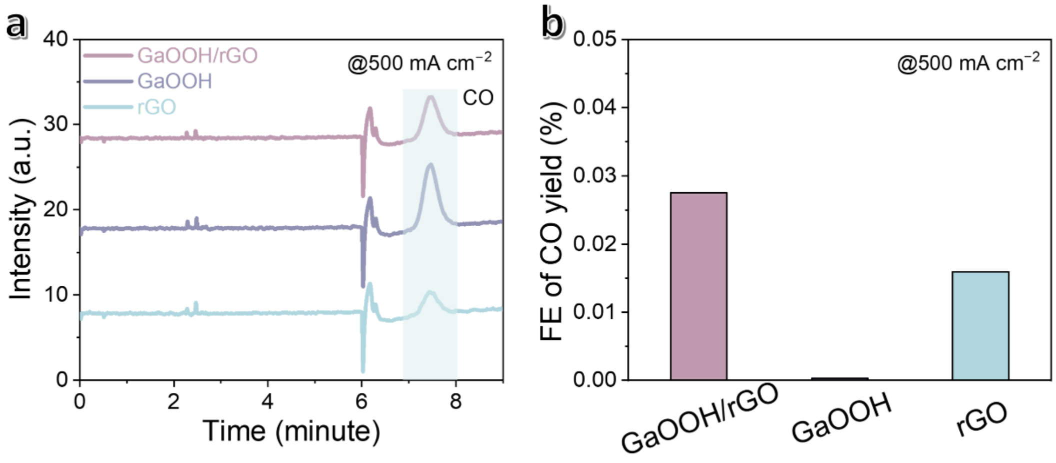

Supplementary Fig. 23. The CO byproduct determination by FID for GaOOH/rGO, GaOOH and rGO at a current density of 500 mA cm^{-2}. **a**, the detected curves of FID. **b**, the calculated Faradaic efficiency of CO. Source data for **a-b** are provided as a Source Data file.

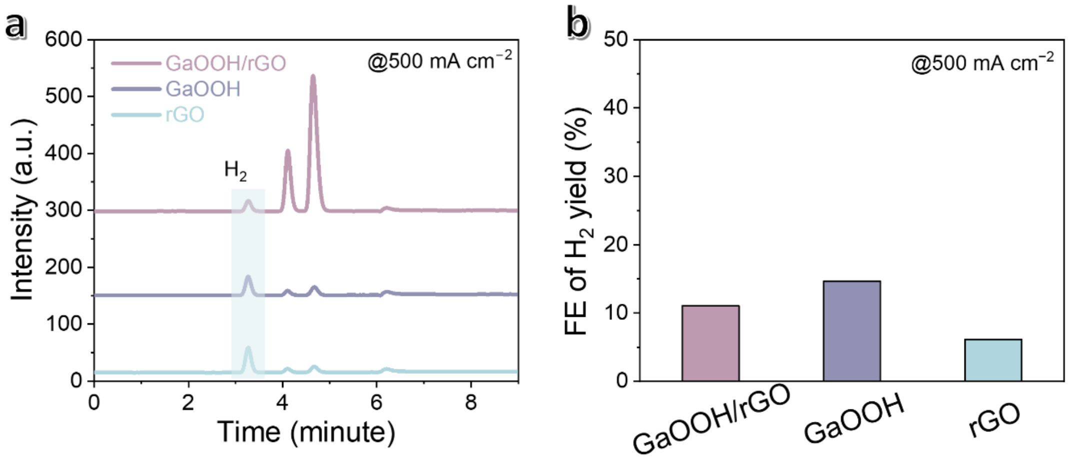

Supplementary Fig. 24. The H_2 byproduct determination by TCD for GaOOH/rGO, GaOOH and rGO at a current density of 500 mA cm^{-2}. **a**, the detected curves of TCD. **b**, the calculated Faradaic efficiency of H_2. Source data for **a-b** are provided as a Source Data file.

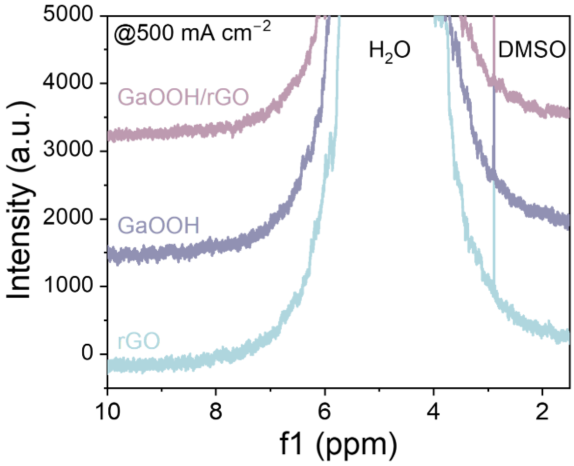

Supplementary Fig. 25. The ^{1}H NMR spectrum of measured electrolytes for GaOOH/rGO, GaOOH and rGO at a current density of 500 mA cm^{-2}. Source data is provided as a Source Data file.

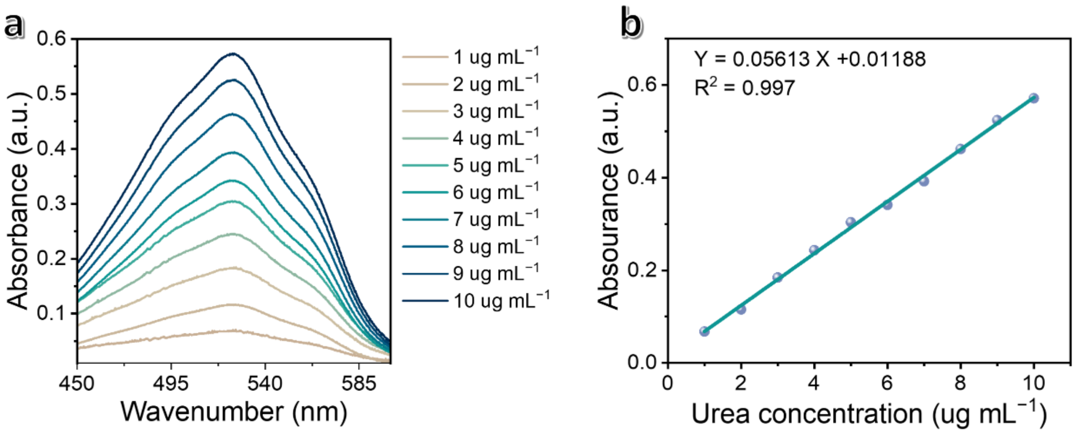

Supplementary Fig. 26. UV-vis calibration curve of urea byproduct. **a**, the UV-vis spectra of standard urea solution with different concentrations. **b**, fitted calibration curve used for estimating urea byproduct. Source data for **a-b** are provided as a Source Data file.

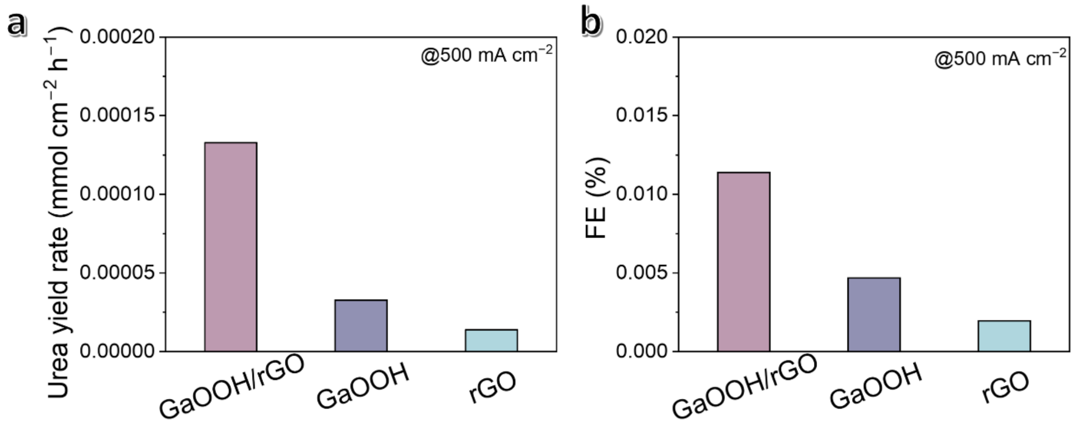

Supplementary Fig. 27. The urea yield rate and Faradaic efficiency for GaOOH/rGO, GaOOH and rGO samples at a current density of 500 mA cm^{-2}. **a**, urea yield rate. **b**, Faradaic efficiency. Source data for **a-b** are provided as a Source Data file.

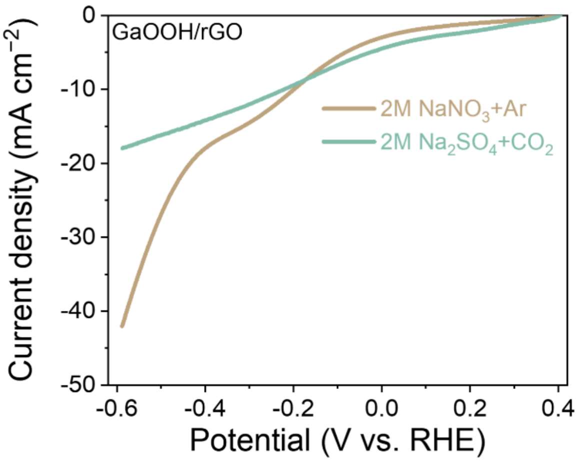

Supplementary Fig. 28. The linear sweep voltammetry curves of GaOOH/rGO catalyst in comparison experiments: in 2M Na_2SO_4 aqueous solution with CO_2 pumping and 2M $NaNO_3$ aqueous solution with Ar pumping. Source data is provided as a Source Data file.

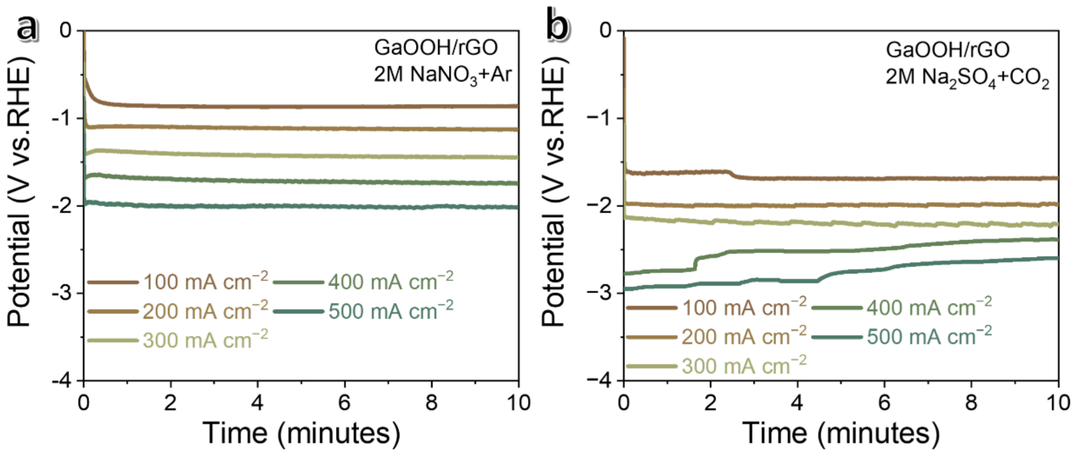

Supplementary Fig. 29. **a,** The chronopotentiometry tests of GaOOH/rGO catalyst at different current densities in 2M $NaNO_3$ aqueous solution with Ar pumping. **b**, The chronopotentiometry tests of GaOOH/rGO catalyst at different current densities in 2M Na_2SO_4 aqueous solution with CO_2 pumping. Source data for **a-b** are provided as a Source Data file.

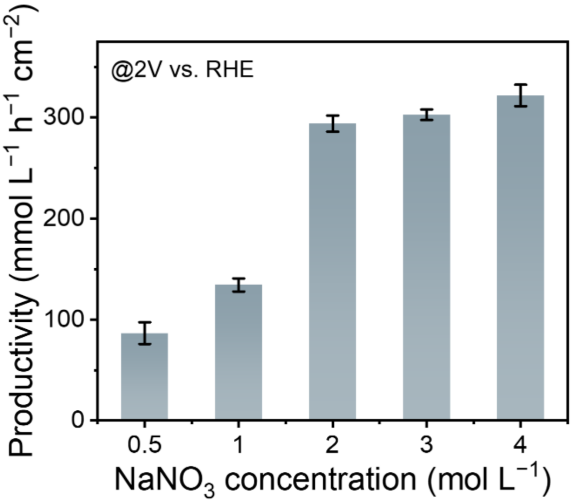

Supplementary Fig. 30. The productivity of $NaHCO_3$ for GaOOH/rGO catalyst in different $NaNO_3$ concentrations at 2 V vs. RHE. The error bars correspond to the standard deviation. The amount of the product $NaHCO_3$ is closely related to the amount of NO_3^-. That is, when the NO_3^- concentration is below 2 M, a higher NO_3^- concentration leads to an increase in $NaHCO_3$ productivity (86.7 mmol L^{-1} cm^{-2} h^{-1} in 0.5 M $NaNO_3$; 134.3 mmol L^{-1} cm^{-2} h^{-1} in 1 M $NaNO_3$). When the NO_3^- concentration is more than 2 M, the increase rate of $NaHCO_3$ productivity becomes slow (293.9 mmol L^{-1} cm^{-2} h^{-1} in 2 M $NaNO_3$; 302.7 mmol L^{-1} cm^{-2} h^{-1} in 3 M $NaNO_3$; 321.7 mmol L^{-1} cm^{-2} h^{-1} in 4 M $NaNO_3$). Source data is provided as a Source Data file.

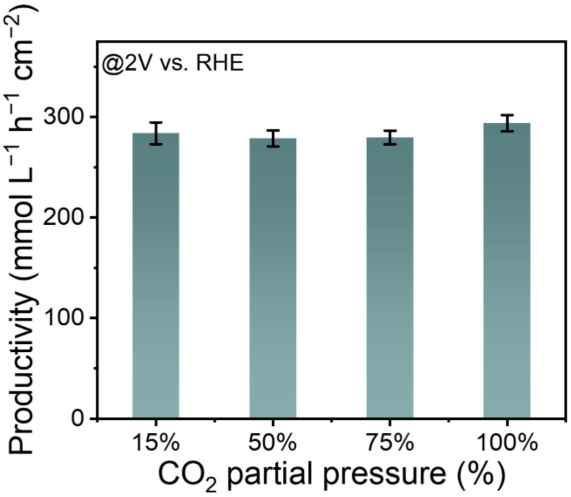

Supplementary Fig. 31. The productivity of $NaHCO_3$ for GaOOH/rGO catalyst in different CO_2 partial pressures at 2 V vs. RHE. The error bars correspond to the standard deviation. When the CO_2 partial pressures range from 15% to 100%, the productivity of $NaHCO_3$ is comparable (from 284.8 to 293.9 mmol L^{-1} cm^{-2} h^{-1}). Therefore, the partial pressure of CO_2 does not have a significant effect on the produced $NaHCO_3$. Source data is provided as a Source Data file.

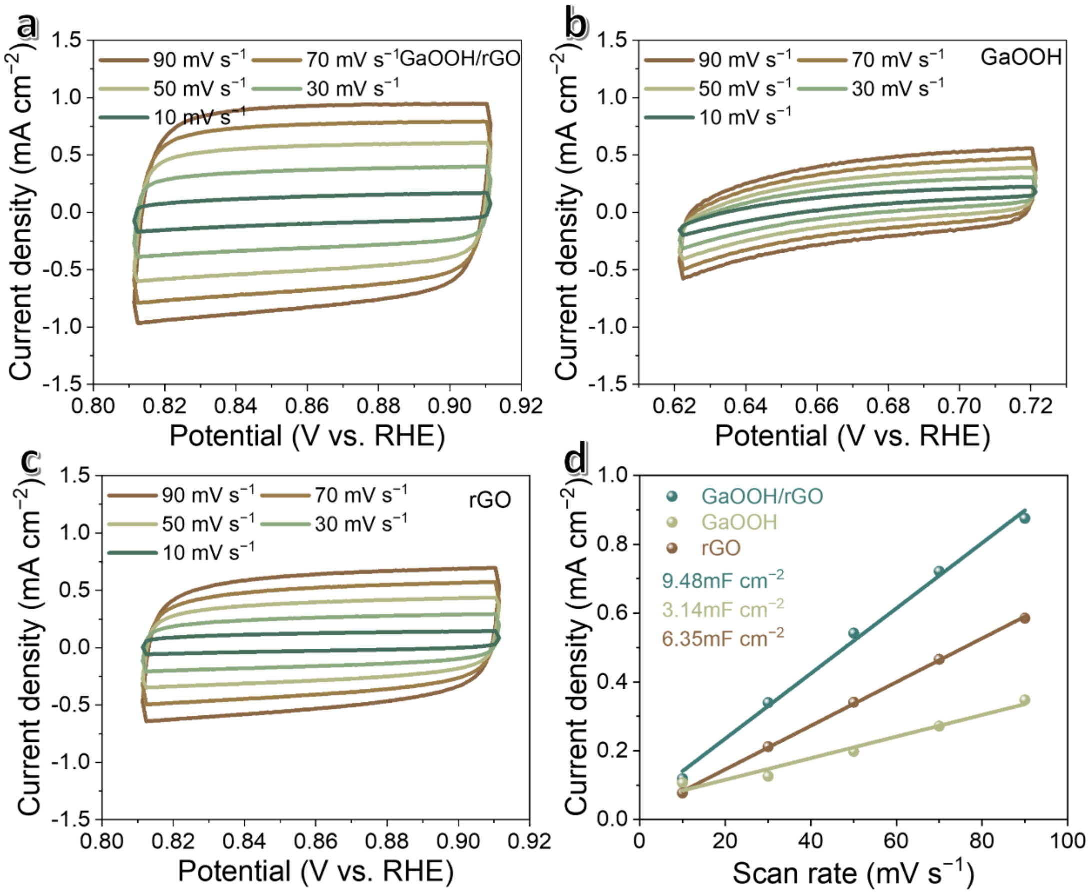

Supplementary Fig. 32. Electrochemical active surface area (ECSA) tests by CVs at different scan rates for **a**, GaOOH/rGO. **b,** GaOOH. and **c**, rGO, respectively. **d**, Linear fit results for electrochemical double-layer capacity (C_{dl}) of GaOOH/rGO, GaOOH and rGO. The experiments demonstrated that GaOOH/rGO has exhibited the highest double-layer capacity of 9.48 mF cm^{-2}, which exceeded both GaOOH (3.14 mF cm^{-2}) and rGO (6.35 mF cm^{-2}). Under electrochemical conditions, the highest ECSA of GaOOH/rGO was conducive to most active sites. This observation indicates that GaOOH/rGO has the capacity of adsorbing more CO_2. Source data for **a-d** are provided as a Source Data file.

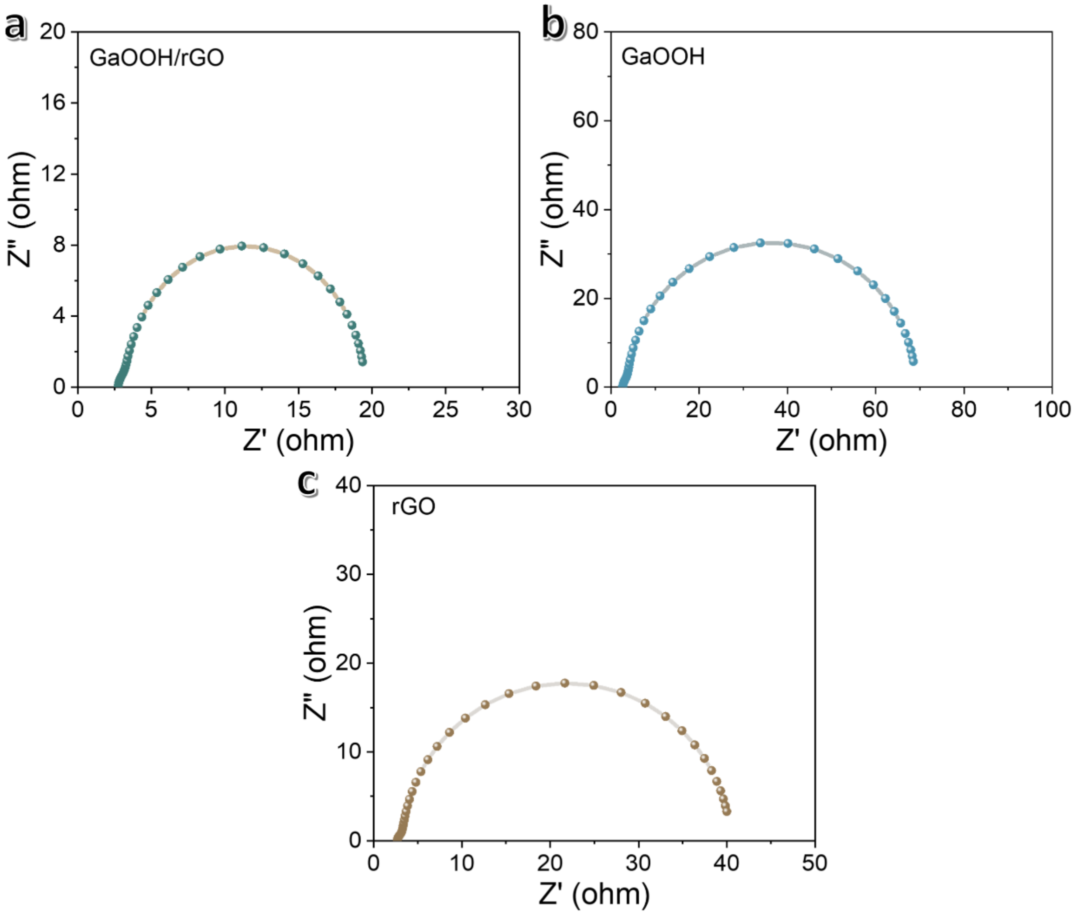

Supplementary Fig. 33. Electrochemical impedance spectroscopy tests (EIS) of **a,** GaOOH/rGO (19.9 Ω), **b,** GaOOH (70.1 Ω), and **c,** rGO (41.5 Ω), respectively. Source data for **a-c** are provided as a Source Data file.

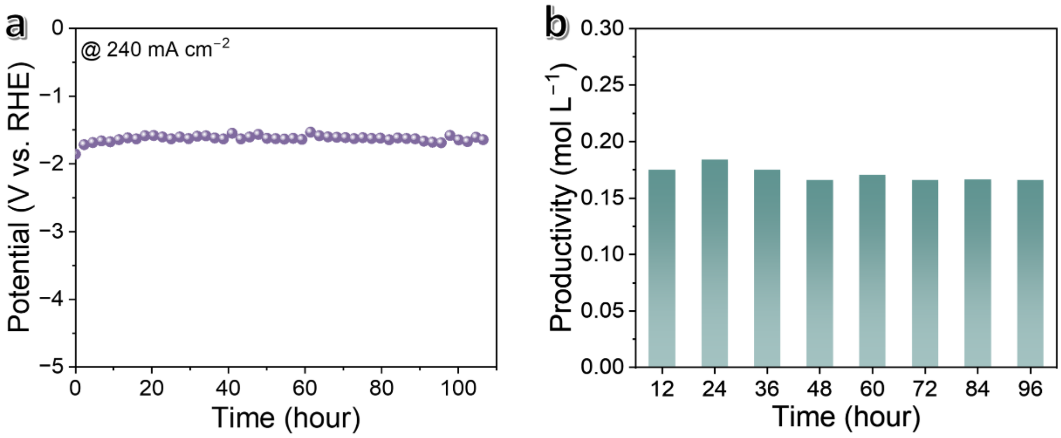

Supplementary Fig. 34. **a**, the 100-hour durability test of GaOOH/rGO catalyst at 240 mA cm^{-2}, operating in a single-pass electrolyte mode. **b**, the accumulated $NaHCO_3$ concentration during durability test at a 12-hour interval. The system has demonstrated stable performances with little potential alteration over 100 hours. The $NaHCO_3$ concentrations have been measured at every 12 hours, which are 0.173 mol L^{-1} for operating 12 hours, 0.182 mol L^{-1} for 24 hours, 0.173 mol L^{-1} for 36 hours, 0.164 mol L^{-1} for 48 hours, 0.169 mol L^{-1} for 60 hours, 0.164 mol L^{-1} for 72 hours, 0.165 mol L^{-1} for 84 hours and 0.164 mol L^{-1} for 96 hours, respectively. Source data for **a-b** are provided as a Source Data file.

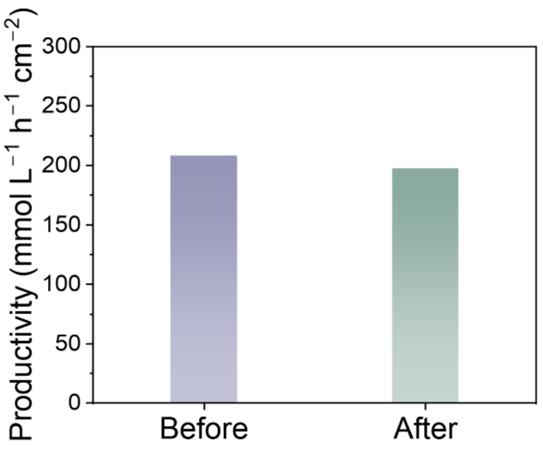

Supplementary Fig. 35. The productivity of $NaHCO_3$ for GaOOH/rGO electrode in 10-minute multiple-cycle tests before and after 100-hour durability test at 240 mA cm^{-2}. Source data fis provided as a Source Data file.

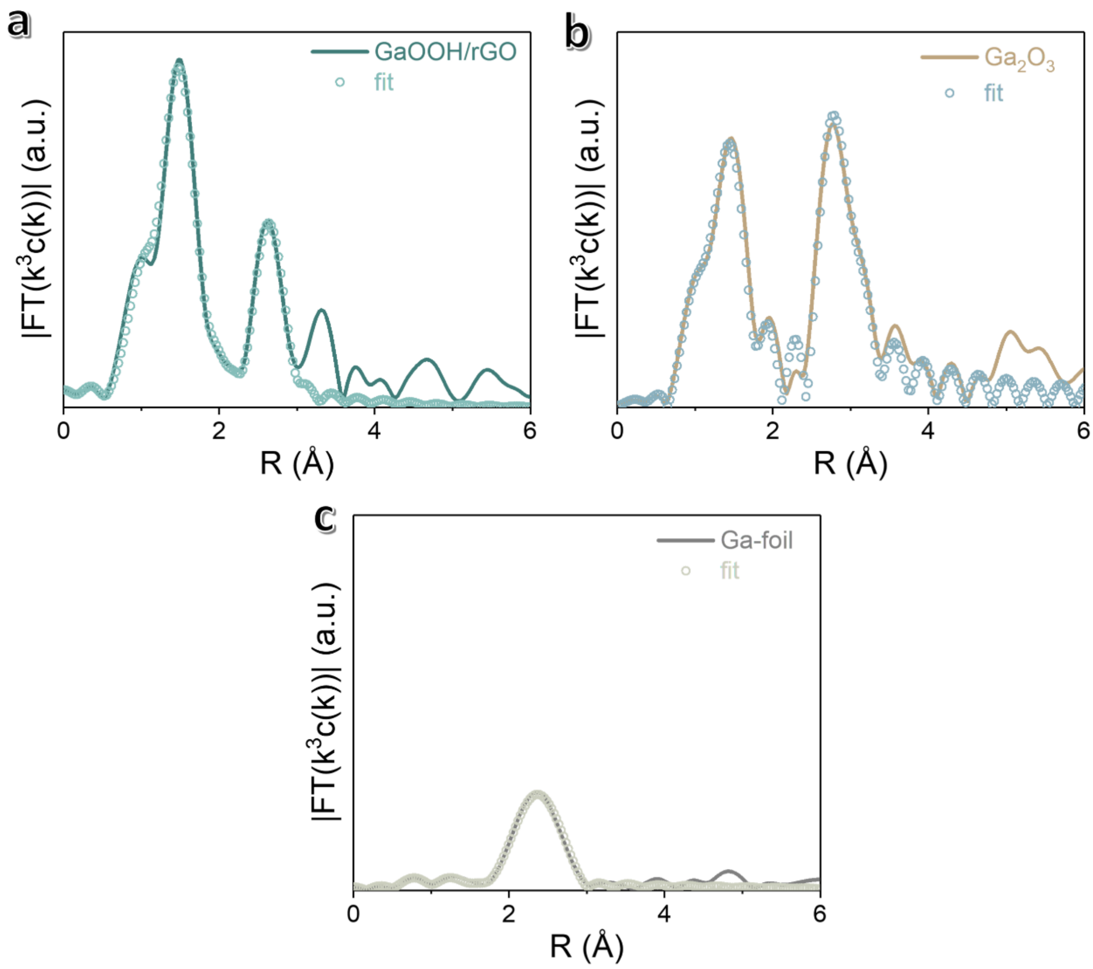

Supplementary Fig. 36. EXAFS curves and the corresponding fitting lines of **a,** GaOOH/rGO **b,** Ga_2O_3 and **c**, Ga-foil, respectively. Source data for **a-c** are provided as a Source Data file.

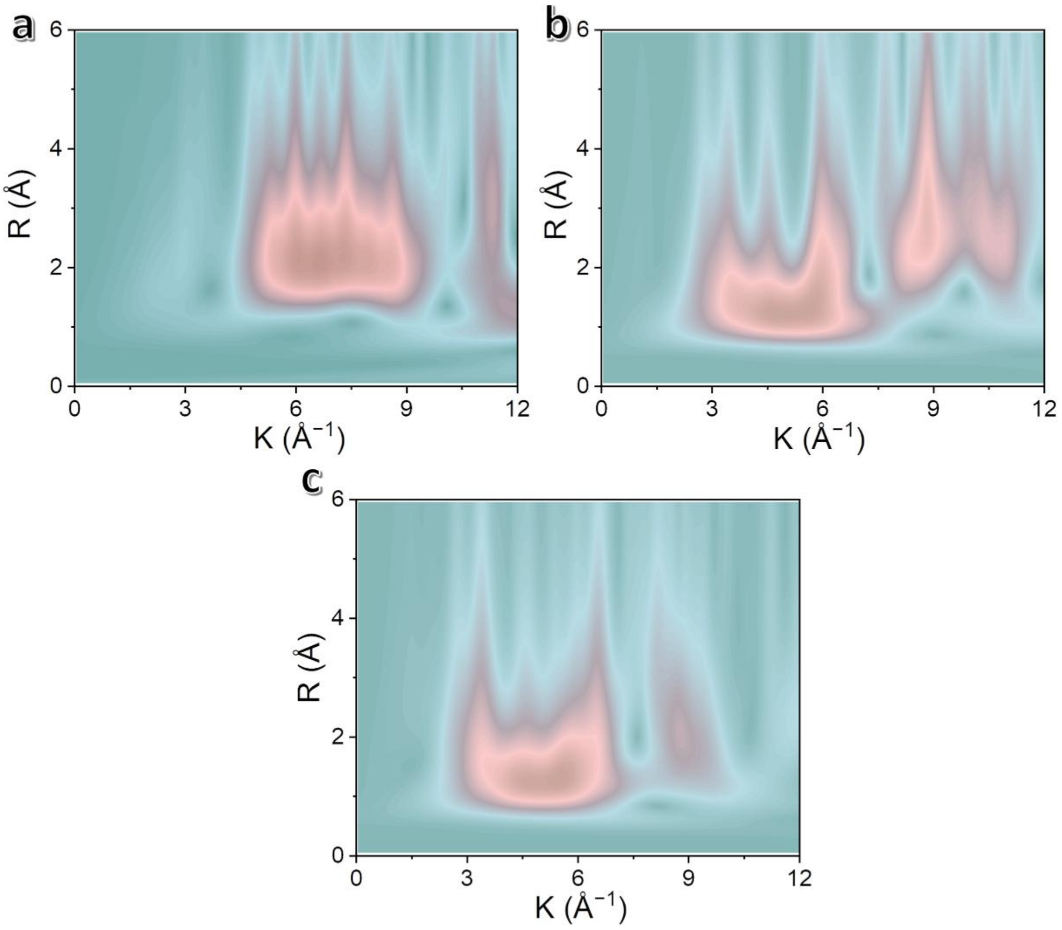

Supplementary Fig. 37. The wavelet transformed EXAFS pattern of **a**, Ga-foil, **b**, Ga_2O_3 and **c**, GaOOH/rGO, respectively. Source data for **a-c** are provided as a Source Data file.

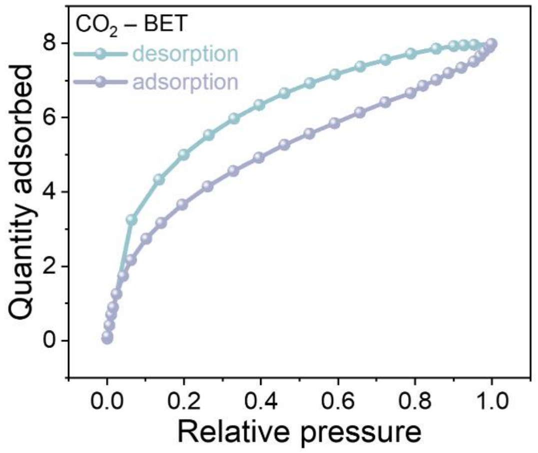

Supplementary Fig. 38. The BET test of GaOOH/rGO in CO_2 atmosphere. The BET results indicate the GaOOH/rGO with a pore area of 62.2 m^2 g^{-1} and a CO_2 adsorption capacity of 7.98 cm^3 g^{-1}. This result indicates GaOOH/rGO exhibiting good CO_2 adsorption capabilities. Source data is provided as a Source Data file.

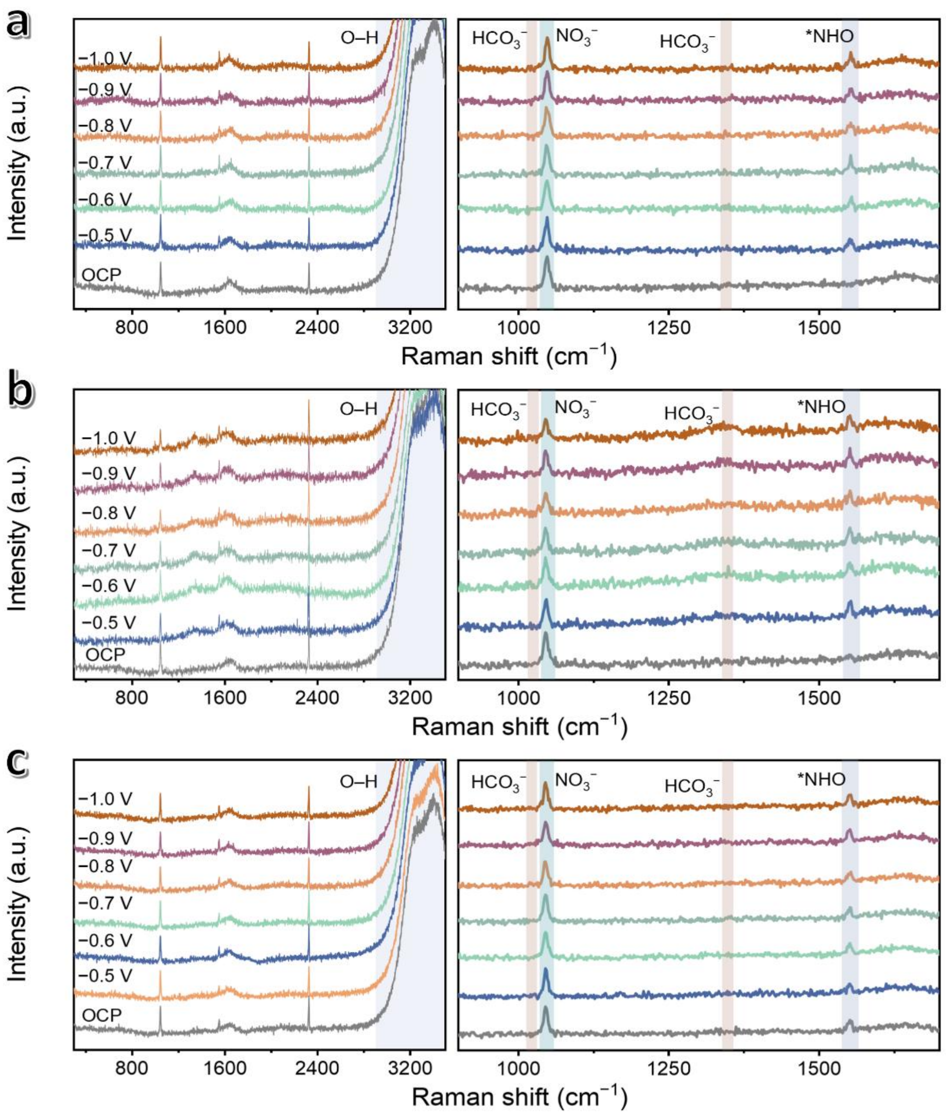

Supplementary Fig. 39. a–c, repetitive *operando* Raman tests for GaOOH/rGO system at the potential range of −0.5 V ~ −1.0 V vs. RHE. Source data for **a-c** are provided as a Source Data file.

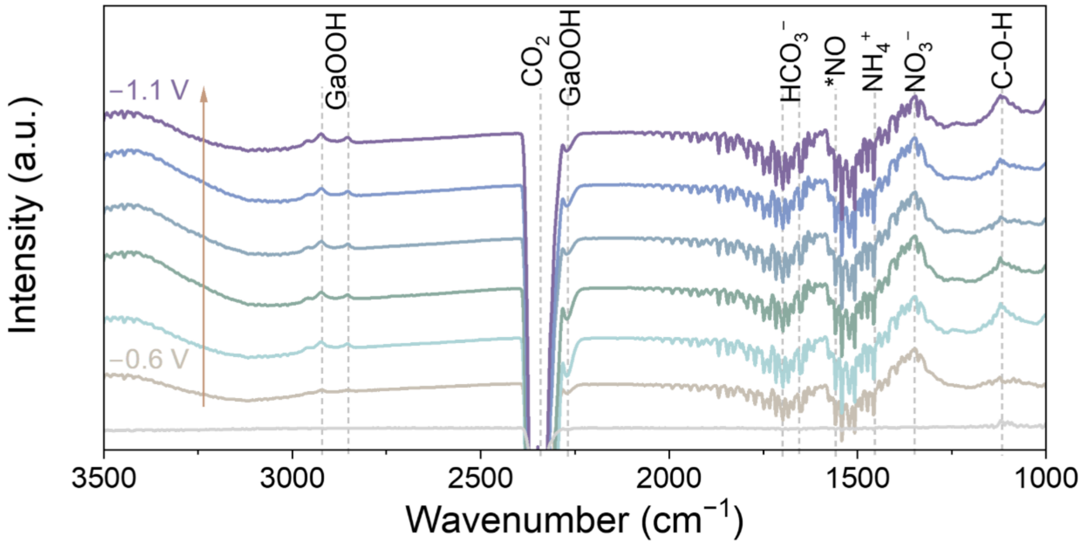

Supplementary Fig. 40. The *operando* FTIR tests for GaOOH/rGO system at the potential range of −0.6 V ~ −1.1 V vs. RHE. Source data is provided as a Source Data file.

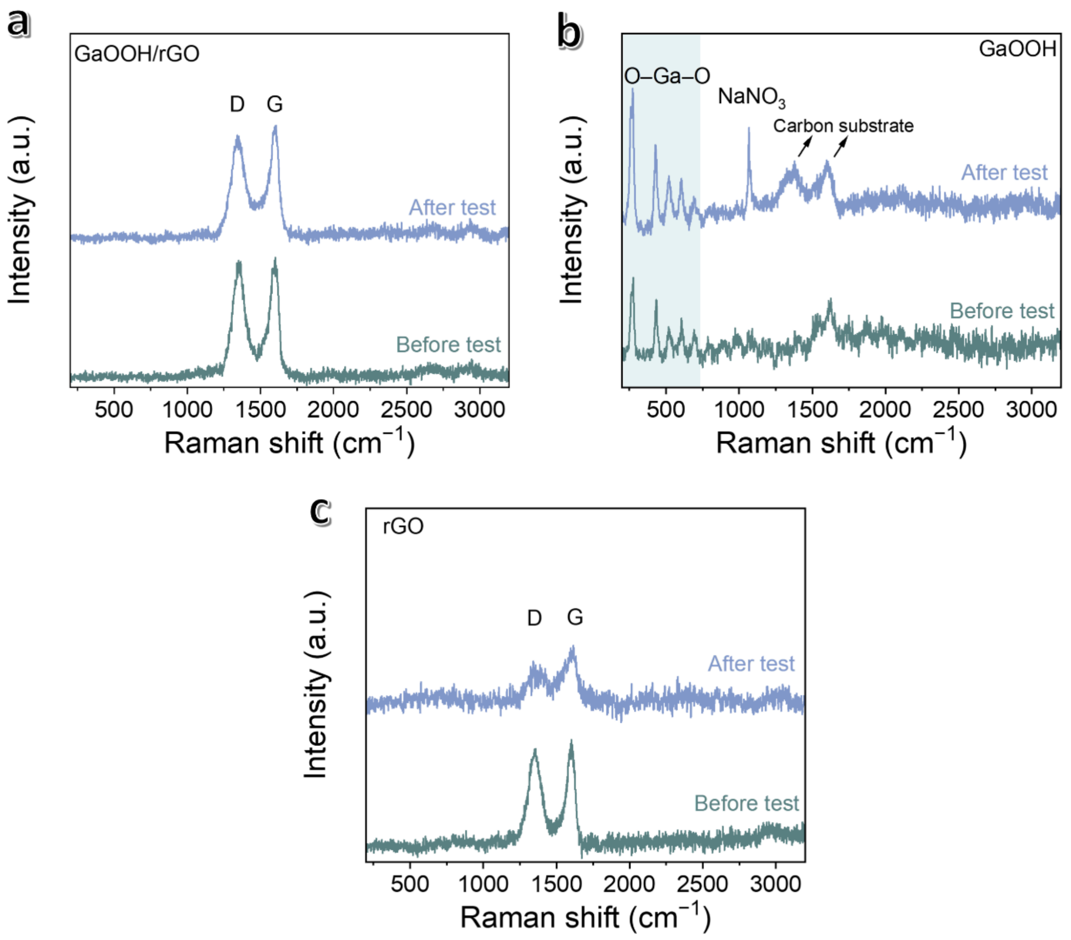

Supplementary Fig. 41. The Raman spectra for different electrodes before and after electrochemical testing. **a**, GaOOH/rGO, **b**, GaOOH and **c**, rGO, respectively. In Raman spectra, GaOOH/rGO has only exhibited D and G bands from carbon species, which is similar to the case of rGO, while there was no significant change in Raman signals before and after electrochemical tests. While for the GaOOH sample, several Raman vibration signals can be observed that belong to Ga–O (272, 429, 515, 605 and 694 cm^{-1}), with seldom change before and after tests. Source data for **a-c** are provided as a Source Data file.

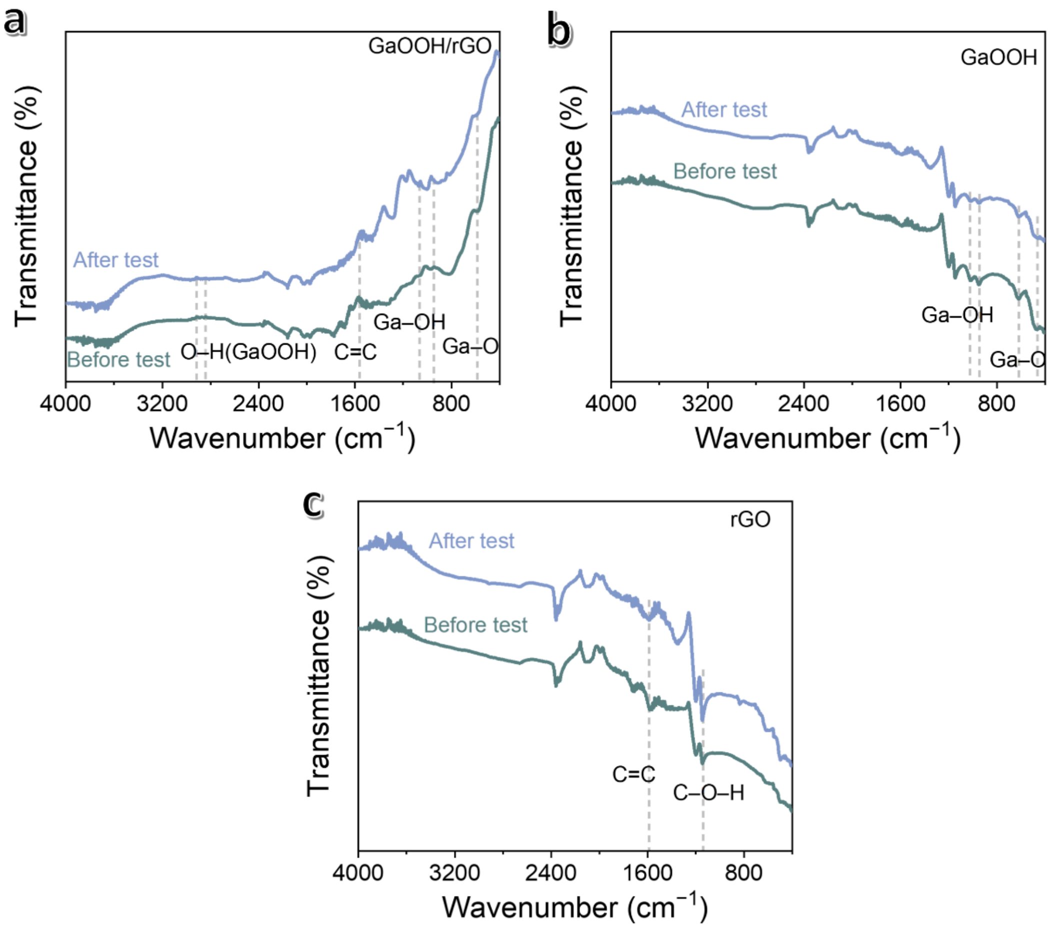

Supplementary Fig. 42. The FTIR spectra for different electrodes before and after electrochemical testing. **a**, GaOOH/rGO, **b**, GaOOH and **c**, rGO, respectively. The GaOOH/rGO has exhibited vibration signals of Ga–O (580 cm^{-1}), Ga–OH (945 and 1065 cm^{-1}), O–H (2841 and 2918 cm^{-1}) and C=C (1558 cm^{-1}), which are similar to GaOOH and different from rGO. Notably, there are seldom changes in peak intensities observed before and after test. Source data for **a-c** are provided as a Source Data file.

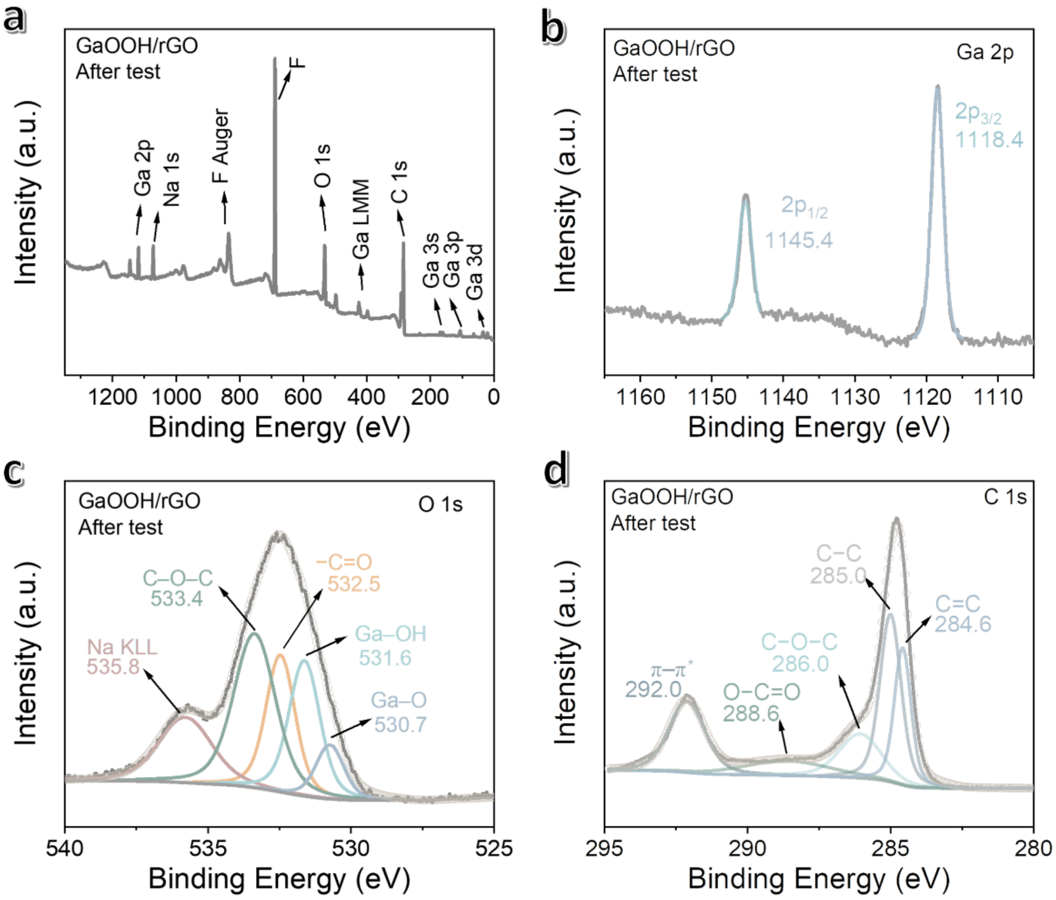

Supplementary Fig. 43. The XPS spectra for GaOOH/rGO catalyst after electrochemical testing. **a**, XPS survey of GaOOH/rGO. **b**, XPS Ga *2p* peaks of GaOOH/rGO. **c**, XPS O *1s* peaks of GaOOH/rGO. **d**, XPS C *1s* peaks of GaOOH/rGO. The Ga *2p* characteristic peak for the GaOOH/rGO catalyst has exhibited a slight shift towards lower binding energy, which is consistent with the characteristics of trivalent Ga (in Ga-metal, the binding energies for $2p_{3/2}$ and $2p_{1/2}$ are 1116.5 and 1143.3 eV, respectively).[17] In the O *1s* spectra, those peaks for Ga–O, Ga–OH, C=O, C–O–C, and Na KLL (derived from electrolyte) can be detected. As comparison to the characteristic peaks before testing, the binding energies of all peaks have exhibited seldom changes. Source data for **a-c** are provided as a Source Data file.

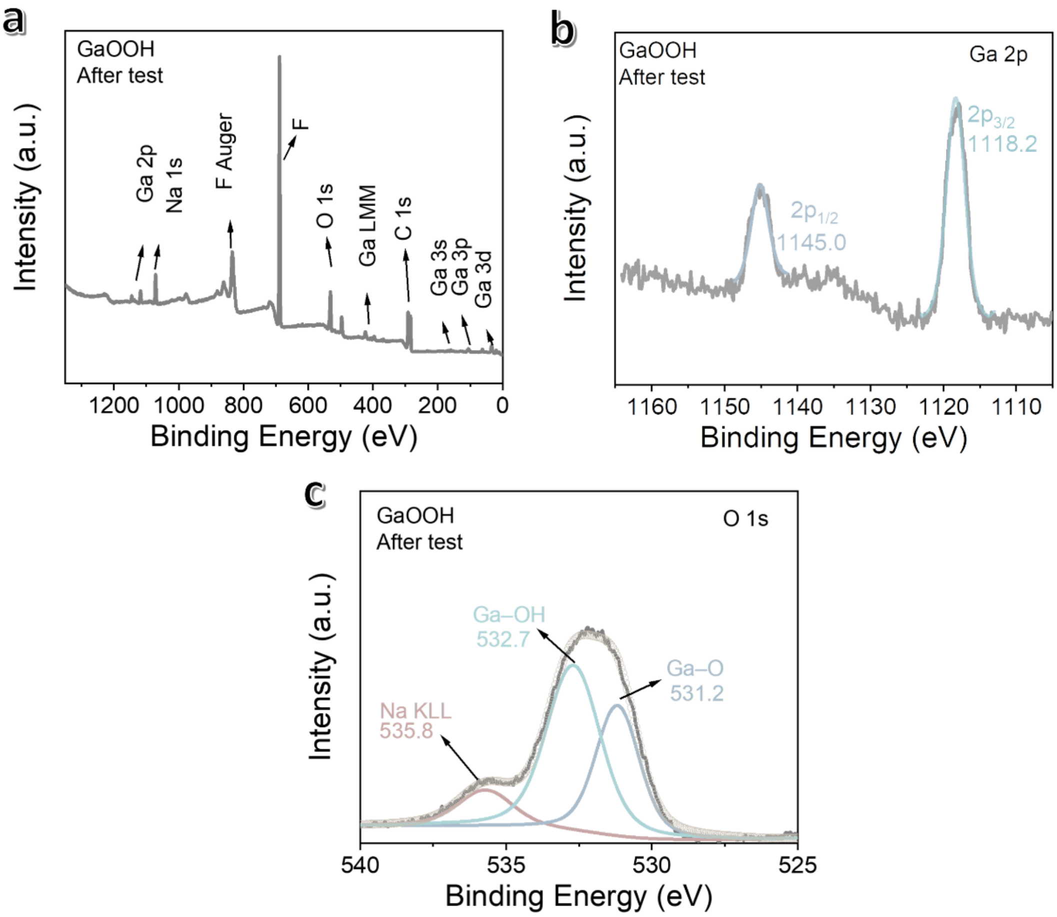

Supplementary Fig. 44. The XPS spectra for GaOOH catalyst after electrochemical testing. **a**, XPS survey of GaOOH. **b**, XPS Ga *2p* peaks of GaOOH. **c**, XPS O *1s* peaks of GaOOH. These characteristic signals show seldom change. Source data for **a-c** are provided as a Source Data file.

Based on above data, the material stability of GaOOH/rGO catalyst has been discussed.

Firstly, the electrodes were characterized by Raman spectra (Supplementary Fig. 41). GaOOH/rGO exhibited the D and G bands from carbon species, which is similar to the case of rGO, and there was no significant change in Raman signals before and after electrochemical testing. In the case of the GaOOH sample, several Raman vibration signals belonging to Ga–O (272, 429, 515, 605 and 694 cm^{-1}) were observed, with no significant alterations noted before and after test.

Secondly, FTIR characterizations were conducted (Supplementary Fig. 42). The vibration signals of Ga–O (580 cm^{-1}), Ga–OH (945 and 1065 cm^{-1}), O–H (2841 and

2918 cm^{-1}), and C=C (1558 cm^{-1}) have been exhibited by the GaOOH/rGO, and these are similar to those of GaOOH and different from those of rGO. It is noteworthy that there is frequently an absence of alterations in peak intensity levels.

Thirdly, XPS was performed before and after electrochemical test (Figure 2c; Supplementary Fig. 6–8; Supplementary Fig. 43–44). The Ga *2p* characteristic peak of the GaOOH/rGO samples has exhibited characteristics of trivalent Ga before and after test (in Ga-metal, the binding energies for $2p_{3/2}$ and $2p_{1/2}$ are 1116.5 and 1143.3 eV, respectively).[17] In the O *1s* spectra, the peaks for Ga–O, Ga–OH, C=O, C–O–C, and Na KLL (derived from electrolyte) were detected. As comparison to the characteristic peaks before the test, the binding energies of all peaks rarely undergo alteration. The positions of these peaks remained relatively static.

It is evident from the experimental data pertaining to Raman, FTIR and XPS that GaOOH/rGO is a stable metal-oxyhydroxide hybrid material. One of the factors contributing to this phenomenon is the *operando* generation of an alkaline environment during the electrocatalytic reduction reaction, which has been shown to contribute to the stability of oxyhydroxide.[18] As stated in previous literature, metal oxyhydroxide has been identified as an active material with the capacity to catalyze the reduction reaction.[19-21] Secondly, the strong interaction between rGO and GaOOH also stabilizes the active valence state of Ga, thereby inhibiting excessive reduction. It can thus be concluded that the oxygen-containing functional groups on the rGO surface form a coordination bond (Ga–O–C) with Ga^{3+}, thereby constraining and preventing excessive electron transfer that leads to the formation of Ga^0.[22, 23] Thirdly, the high conductivity of rGO promotes the directed transfer of electrons to active sites, thus avoiding the formation of elemental Ga due to excess electrons.[24] Consequently, despite Ga requiring electrons to initiate the reduction reaction (*e.g.*, the reduction of the target substrate NO_3^-), the high conductivity of rGO can efficiently transfer electrons to the Ga sites, thereby inhibiting over-reduction of Ga^{3+} to Ga^0.

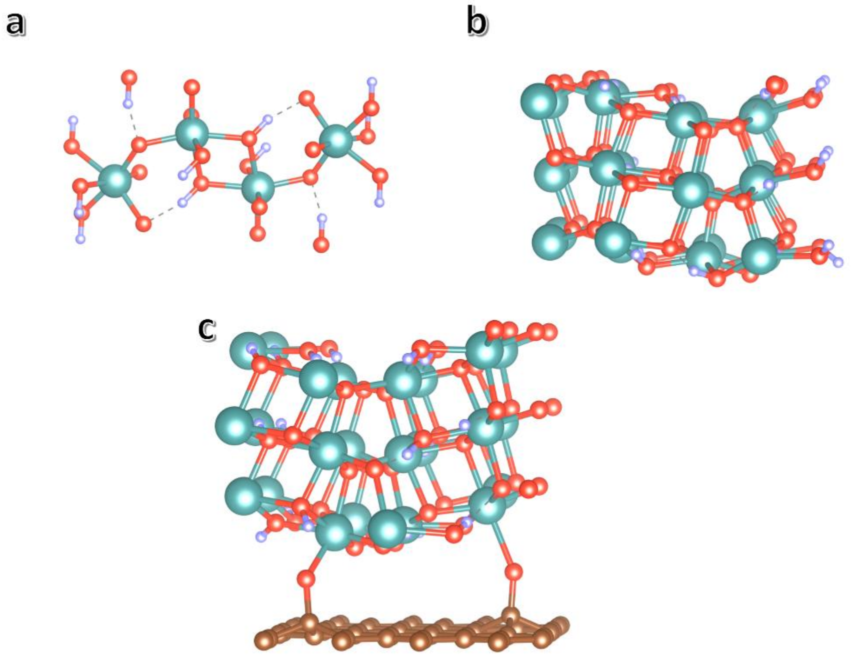

Supplementary Fig. 45. Theoretical structural models for DFT calculations. **a,** The unit cell structure of GaOOH. **b**, The supercell structure of the lattice plane (111) for GaOOH. **c**, The supercell structure of GaOOH/rGO.

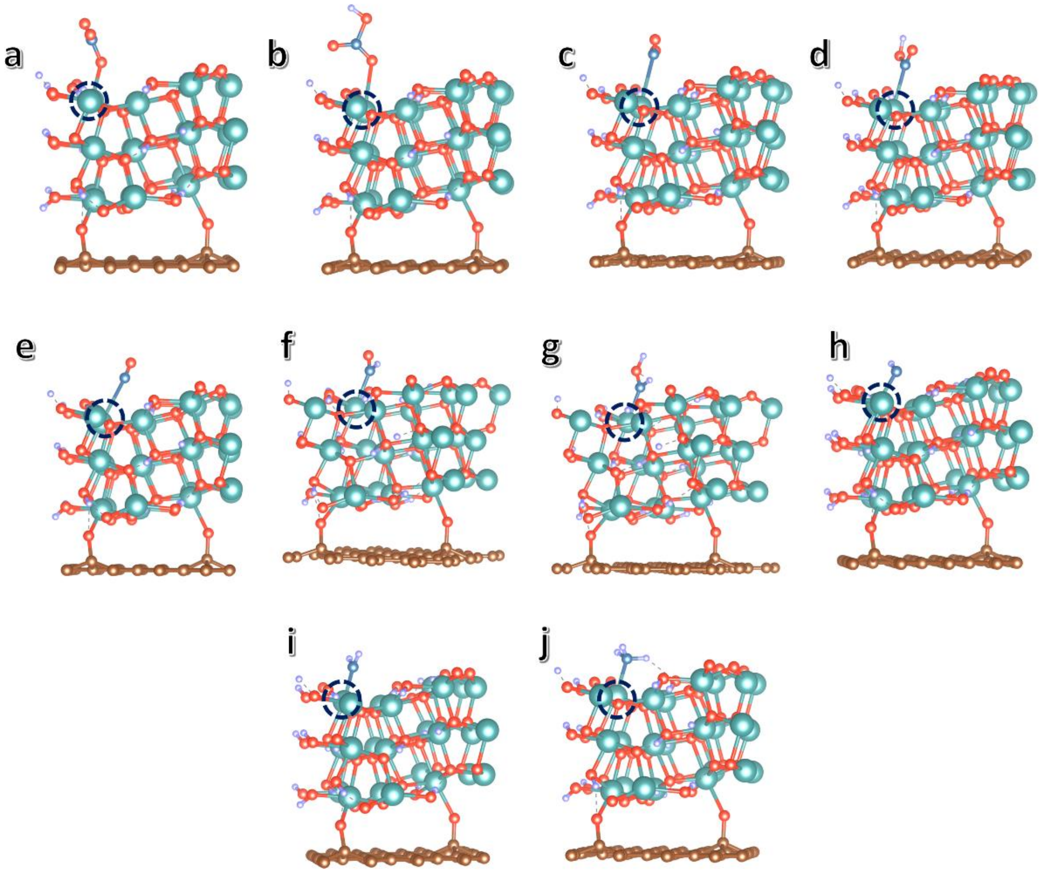

Supplementary Fig. 46. The optimized structures of reaction intermediates for NO_3RR on GaOOH/rGO surface, where the adsorbates from **a**–**j** are $*NO_3^-$, $*NO_3H$, $*NO_2$, $*NO_2H$, *NO, *NHO, *NHOH, *NH, $*NH_2$ and $*NH_3$, respectively.

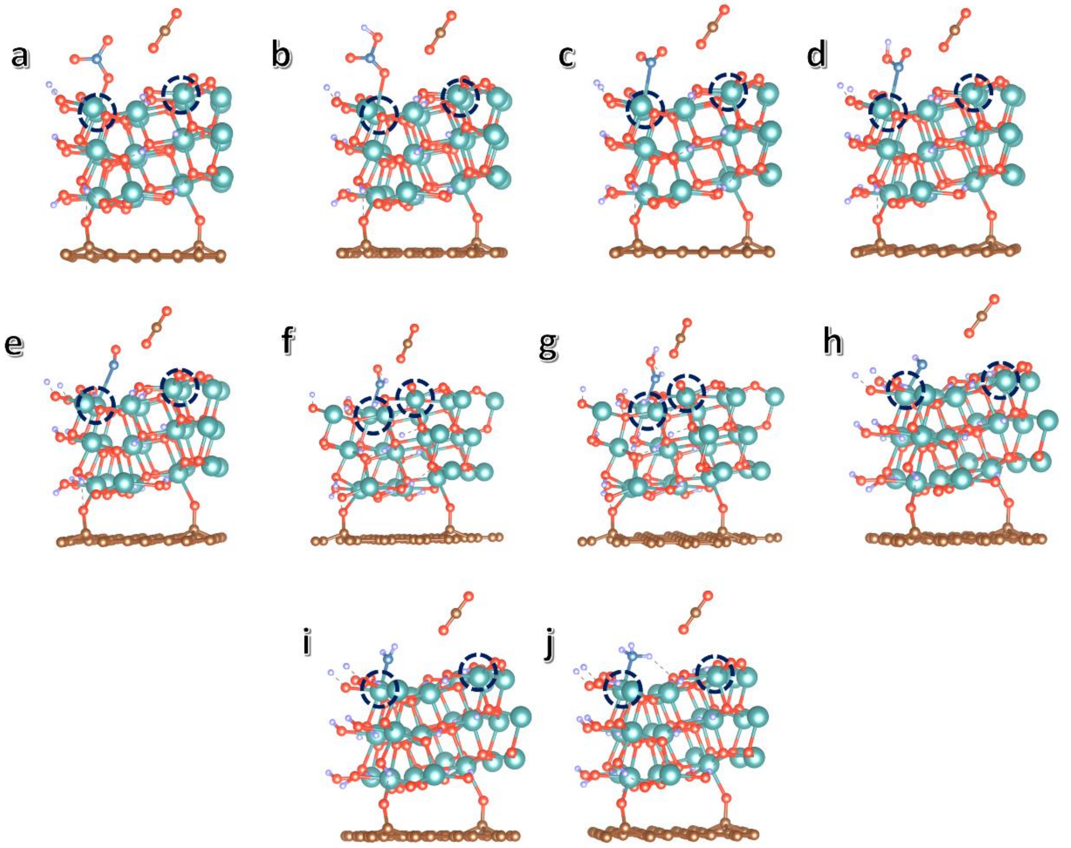

Supplementary Fig. 47. The optimized structures of reaction intermediates for NO_3RR on GaOOH/rGO surface with CO_2, where the adsorbates from **a**–**j** are *NO_3^-, *NO_3H, *NO_2, *NO_2H, *NO, *NHO, *NHOH, *NH, *NH_2 and *NH_3, respectively.

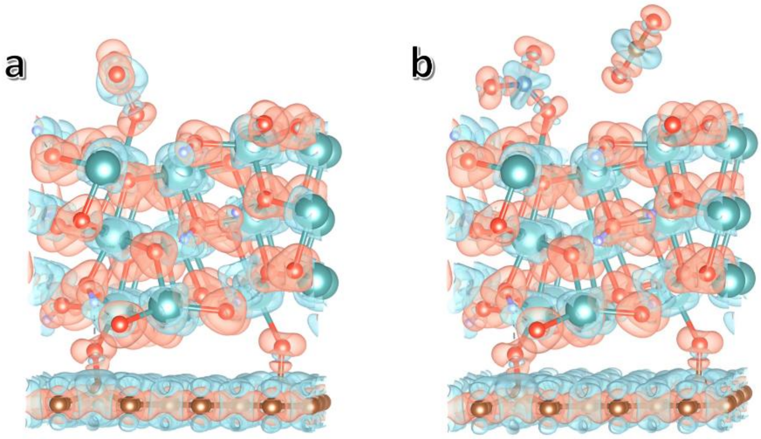

Supplementary Fig. 48. **a**, difference charge density of adsorbed $*NO_3^-$ on GaOOH/rGO. **b**, difference charge density of adsorbed $*NO_3^-$ on GaOOH/rGO covering with CO_2. Red and bule spheres represent charge accumulation and depletion, respectively.

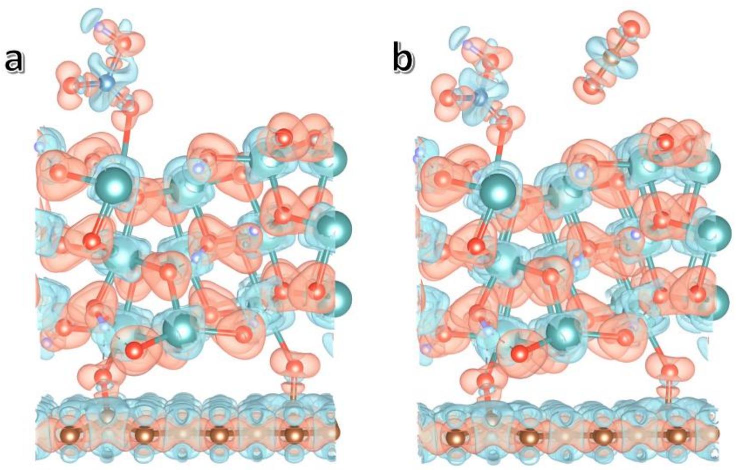

Supplementary Fig. 49. **a**, difference charge density of adsorbed *NO_3H on GaOOH/rGO. **b**, difference charge density of adsorbed *NO_3H on GaOOH/rGO covering with CO_2. Red and bule spheres represent charge accumulation and depletion, respectively.

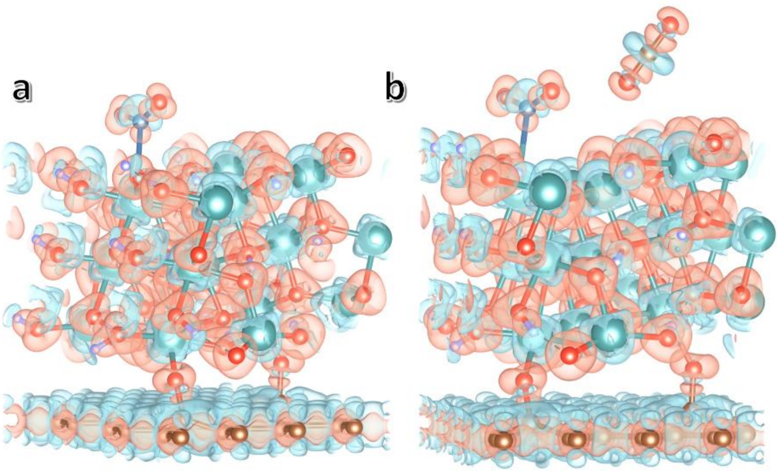

Supplementary Fig. 50. a, difference charge density of adsorbed *NO_2 on GaOOH/rGO. **b**, difference charge density of adsorbed *NO_2 on GaOOH/rGO covering with CO_2. Red and bule spheres represent charge accumulation and depletion, respectively.

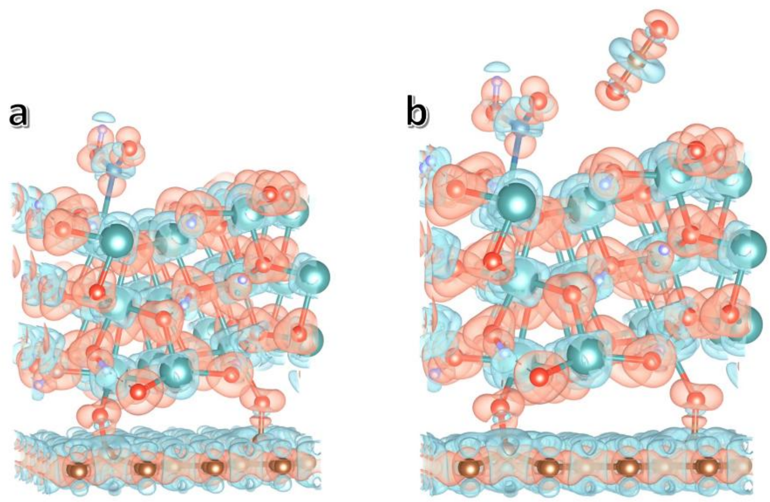

Supplementary Fig. 51. **a**, difference charge density of adsorbed *NO_2H on GaOOH/rGO. **b**, difference charge density of adsorbed *NO_2H on GaOOH/rGO covering with CO_2. Red and bule spheres represent charge accumulation and depletion, respectively.

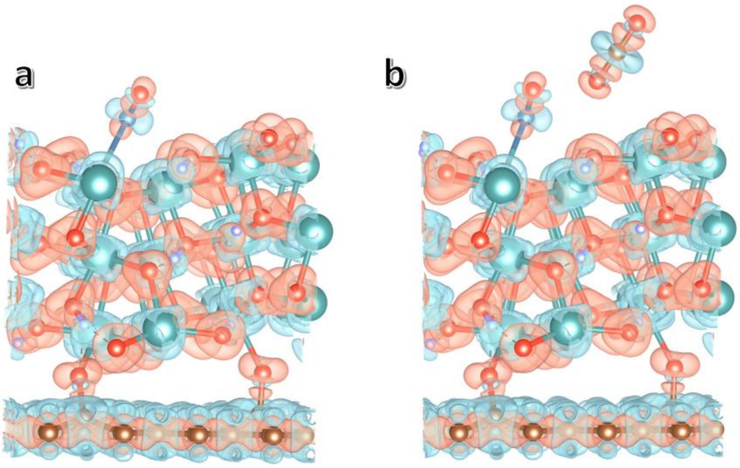

Supplementary Fig. 52. **a**, difference charge density of adsorbed *NO on GaOOH/rGO. **b**, difference charge density of adsorbed *NO on GaOOH/rGO covering with CO_2. Red and bule spheres represent charge accumulation and depletion, respectively.

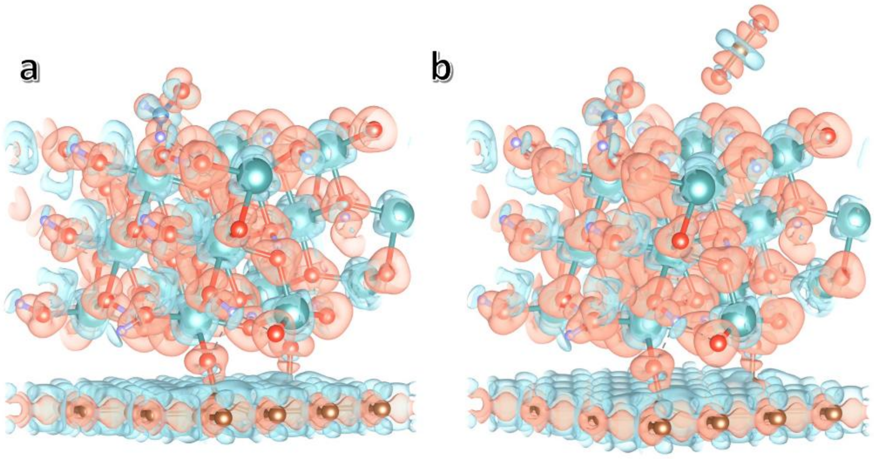

Supplementary Fig. 53. a, difference charge density of adsorbed *NHO on GaOOH/rGO. **b**, difference charge density of adsorbed *NHO on GaOOH/rGO covering with CO_2. Red and bule spheres represent charge accumulation and depletion, respectively.

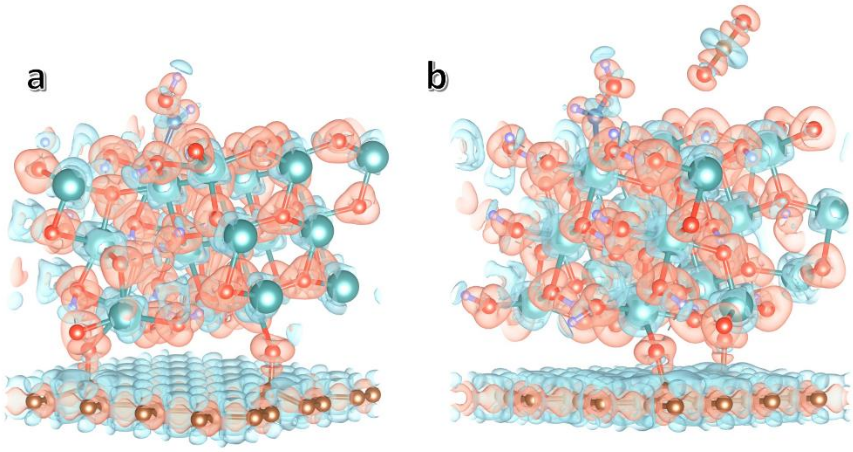

Supplementary Fig. 54. a, difference charge density of adsorbed *NHOH on GaOOH/rGO. **b**, difference charge density of adsorbed *NHOH on GaOOH/rGO covering with CO_2. Red and bule spheres represent charge accumulation and depletion, respectively.

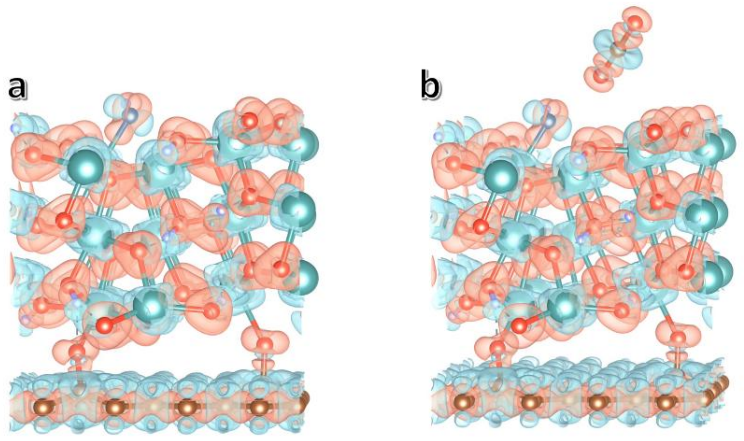

Supplementary Fig. 55. **a**, difference charge density of adsorbed *NH on GaOOH/rGO. **b**, difference charge density of adsorbed *NH on GaOOH/rGO covering with CO_2. Red and bule spheres represent charge accumulation and depletion, respectively.

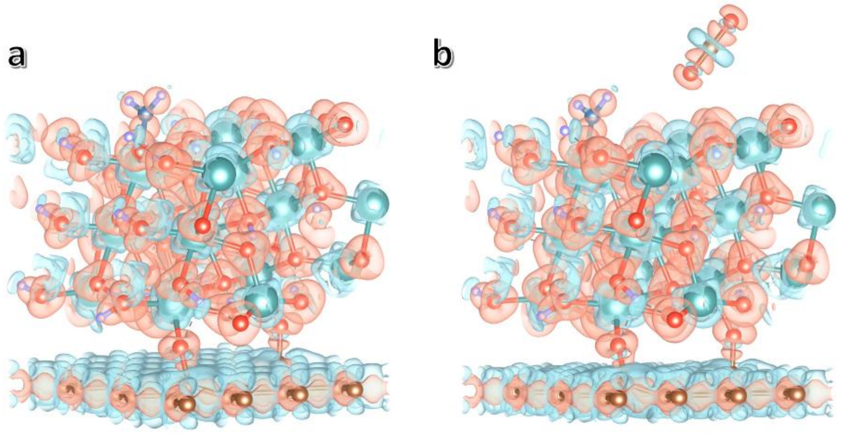

Supplementary Fig. 56. **a**, difference charge density of adsorbed *NH_2 on GaOOH/rGO. **b**, difference charge density of adsorbed *NH_2 on GaOOH/rGO covering with CO_2. Red and bule spheres represent charge accumulation and depletion, respectively.

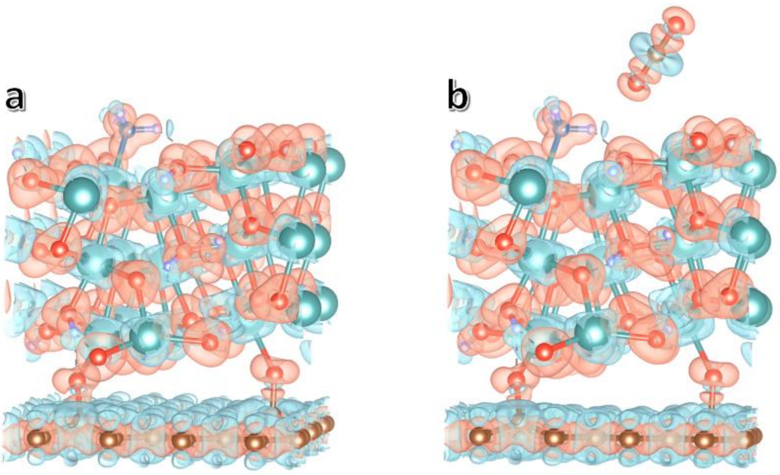

Supplementary Fig. 57. a, difference charge density of adsorbed *NH_3 on GaOOH/rGO. **b**, difference charge density of adsorbed *NH_3 on GaOOH/rGO covering with CO_2. Red and bule spheres represent charge accumulation and depletion, respectively.

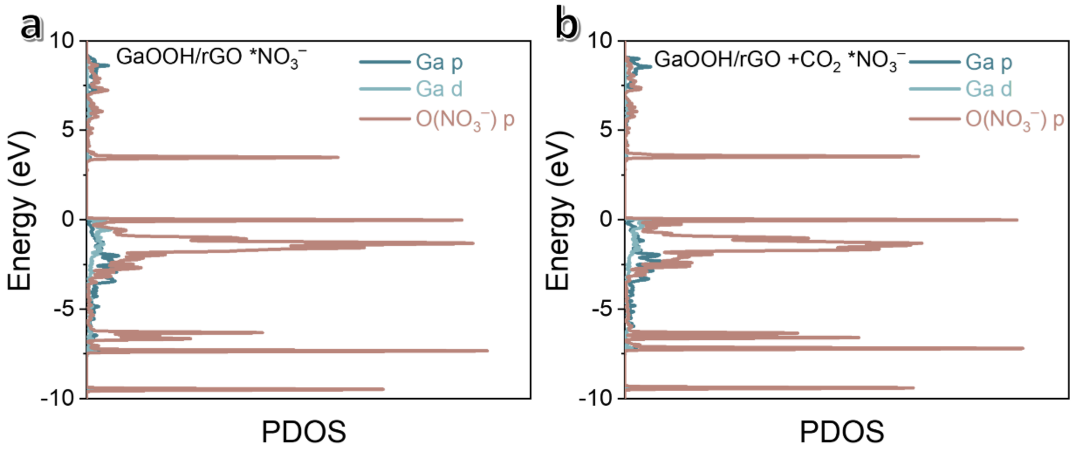

Supplementary Fig. 58. a, partial density of states (PDOS) of adsorbed $*NO_3^-$ on GaOOH/rGO. **b**, partial density of states (PDOS) of adsorbed $*NO_3^-$ on GaOOH/rGO covering with CO_2. Source data for **a-b** are provided as a Source Data file.

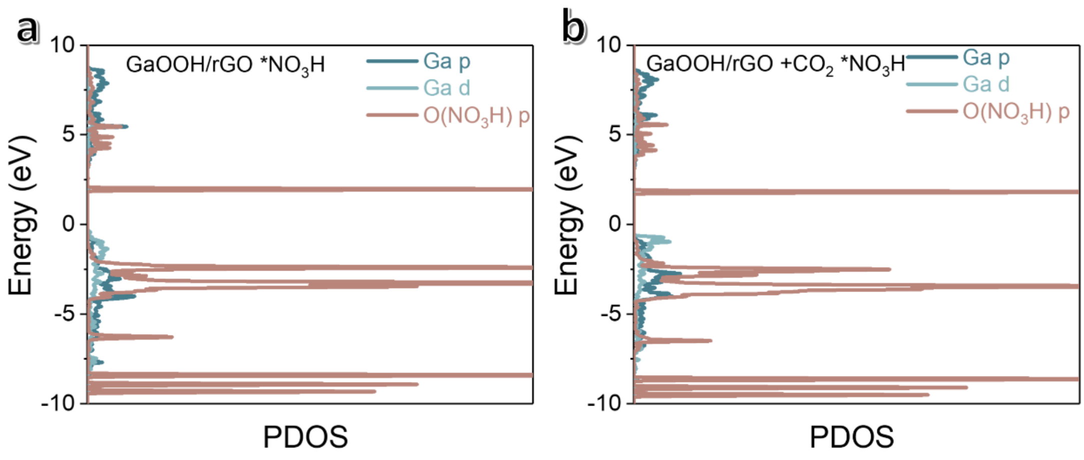

Supplementary Fig. 59. a, partial density of states (PDOS) of adsorbed *NO_3H on GaOOH/rGO. **b**, partial density of states (PDOS) of adsorbed *NO_3H on GaOOH/rGO covering with CO_2. Source data for **a-b** are provided as a Source Data file.

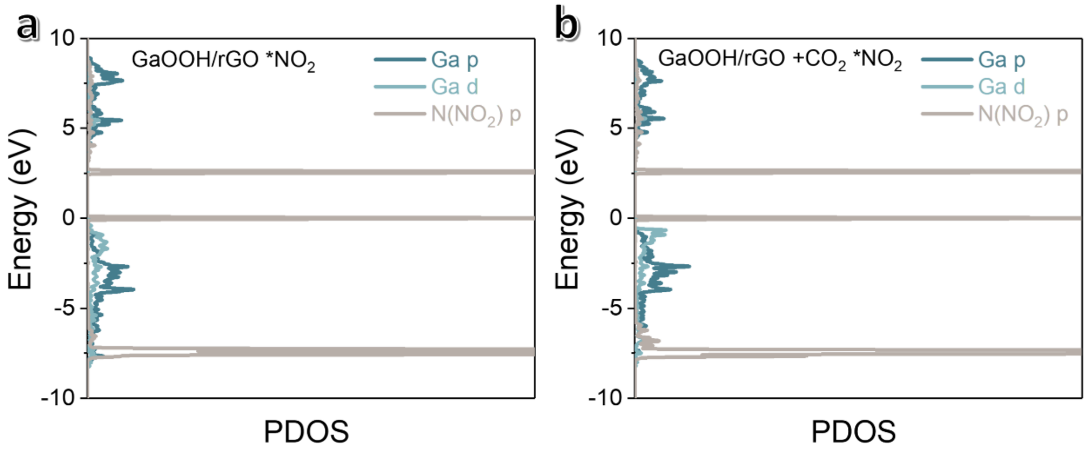

Supplementary Fig. 60. a, partial density of states (PDOS) of adsorbed $*NO_2$ on GaOOH/rGO. **b**, partial density of states (PDOS) of adsorbed $*NO_2$ on GaOOH/rGO covering with CO_2. Source data for **a-b** are provided as a Source Data file.

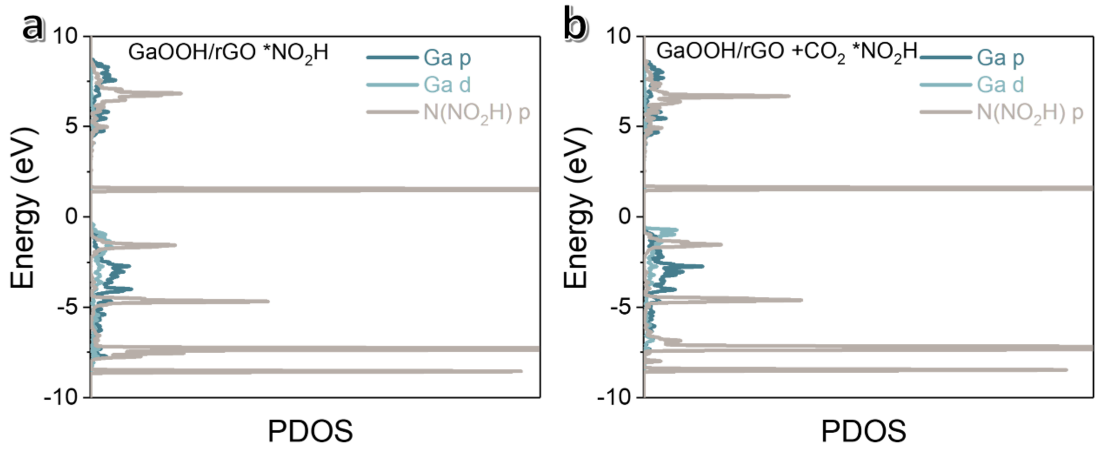

Supplementary Fig. 61. a, partial density of states (PDOS) of adsorbed $*NO_2H$ on GaOOH/rGO. **b**, partial density of states (PDOS) of adsorbed $*NO_2H$ on GaOOH/rGO covering with CO_2. Source data for **a-b** are provided as a Source Data file.

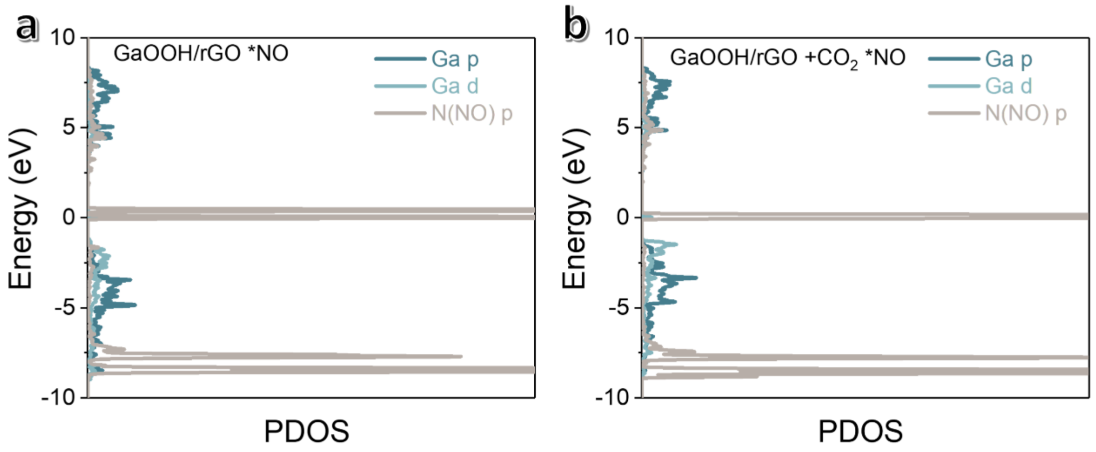

Supplementary Fig. 62. a, partial density of states (PDOS) of adsorbed *NO on GaOOH/rGO. **b**, partial density of states (PDOS) of adsorbed *NO on GaOOH/rGO covering with CO_2. Source data for **a-b** are provided as a Source Data file.

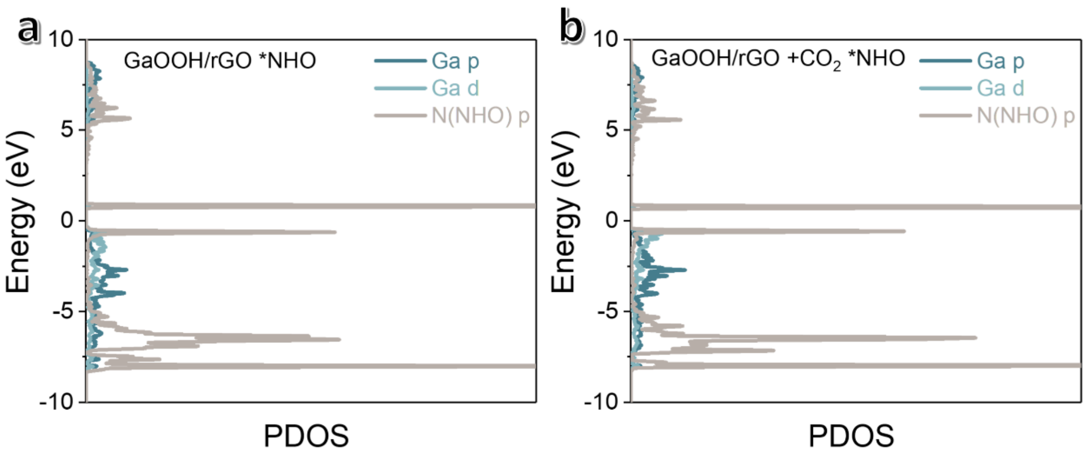

Supplementary Fig. 63. **a**, partial density of states (PDOS) of adsorbed *NHO on GaOOH/rGO. **b**, partial density of states (PDOS) of adsorbed *NHO on GaOOH/rGO covering with CO_2. Source data for **a-b** are provided as a Source Data file.

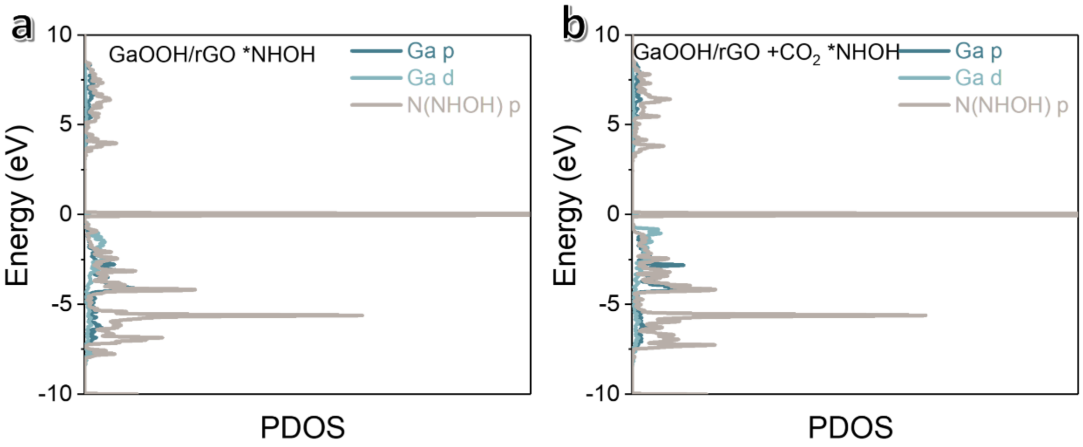

Supplementary Fig. 64. **a**, partial density of states (PDOS) of adsorbed *NHOH on GaOOH/rGO. **b**, partial density of states (PDOS) of adsorbed *NHOH on GaOOH/rGO covering with CO_2. Source data for **a-b** are provided as a Source Data file.

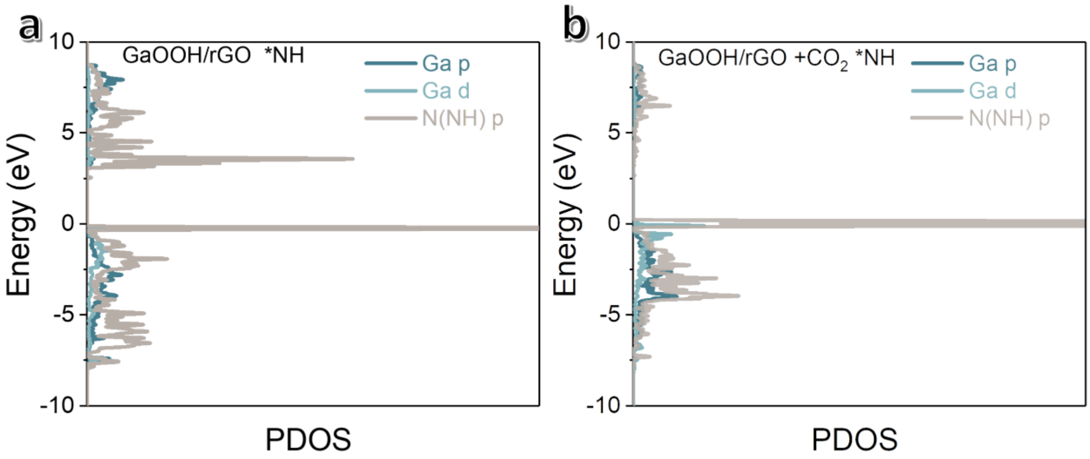

Supplementary Fig. 65. a, partial density of states (PDOS) of adsorbed *NH on GaOOH/rGO. **b**, partial density of states (PDOS) of adsorbed *NH on GaOOH/rGO covering with CO_2. Source data for **a-b** are provided as a Source Data file.

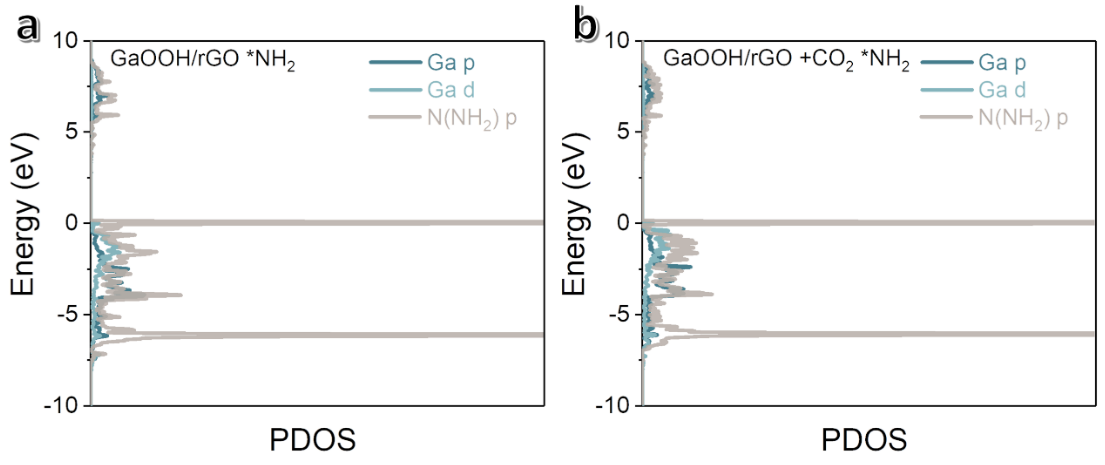

Supplementary Fig. 66. a, partial density of states (PDOS) of adsorbed $*NH_2$ on GaOOH/rGO. **b**, partial density of states (PDOS) of adsorbed $*NH_2$ on GaOOH/rGO covering with CO_2. Source data for **a-b** are provided as a Source Data file.

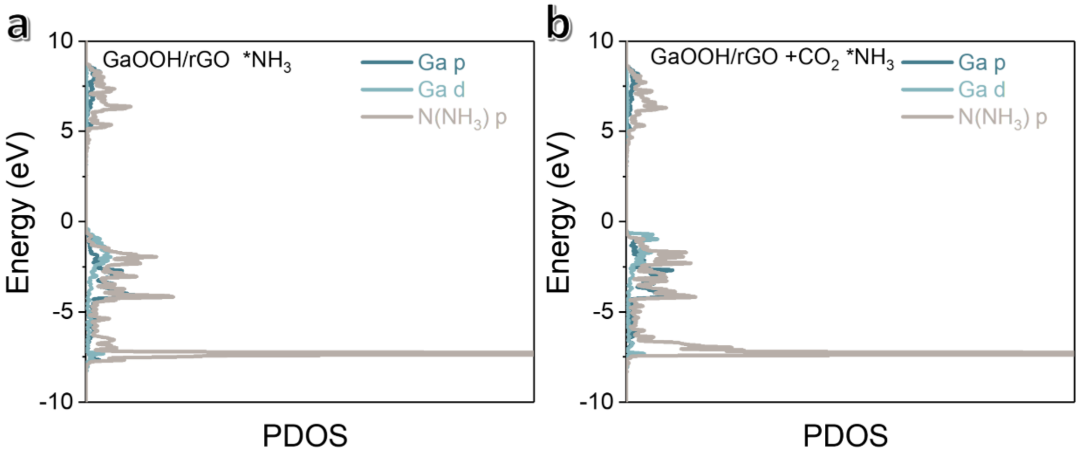

Supplementary Fig. 67. a, partial density of states (PDOS) of adsorbed *NH_3 on GaOOH/rGO. **b**, partial density of states (PDOS) of adsorbed *NH_3 on GaOOH/rGO covering with CO_2. Source data for **a-b** are provided as a Source Data file.

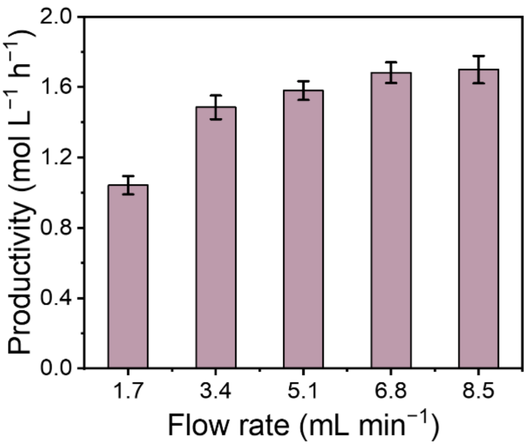

Supplementary Fig. 68. The $NaHCO_3$ productivities of GaOOH/rGO at a current of 2 A in different flow rates of electrolytes. Source data is provided as a Source Data file.

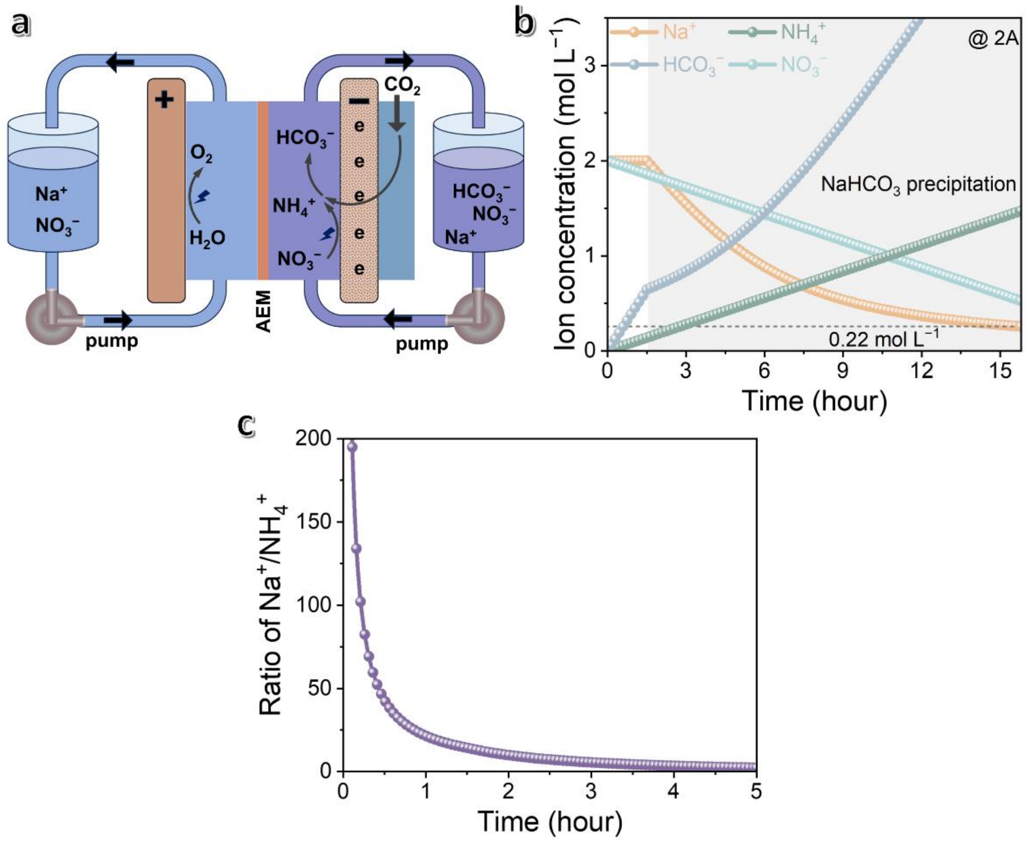

Supplementary Fig. 69. a, the schematic diagram of a cyclic system for *operando*-electrified Solvay process. **b**, the simulated concentrations of Na^+, NH_4^+, HCO_3^- and NO_3^- with reaction durations. **c**, the change of Na^+/NH_4^+ concentration ratios with reaction duration, which is an indication of $NaHCO_3/NH_4HCO_3$ ratio. Source data for **a-c** are provided as a Source Data file.

The calculation details are listed as follows: the initial solution was 50 mL of 2M $NaNO_3$. Following the durability test, the applied current was assumed to be 2 A, and yield rate of HCO_3^- was 0.43 mol L^{-1} h^{-1}. Thereby, the yield rate of NH_4^+ was calculated according to Faraday's law:

$$v_{NH_4^+} = \frac{I \cdot \eta \cdot 3600}{8 \cdot F \cdot V_0} \tag{41}$$

Where $v_{NH_4^+}$ means yield rate. I, η and F represent current, FE and faraday constant, respectively. V_0 is the volume of electrolyte.

Step 1: no precipitation

$$\left[NO_3^-\right]_t = \left[NO_3^-\right]_{t-\Delta t} - v_{NH_4^+} \cdot \Delta t \tag{42}$$

$$\left[Na^+\right]_t = \left[Na^+\right]_{t-\Delta t} \tag{43}$$

$$\left[NH_4^+\right]_t = \left[NH_4^+\right]_{t-\Delta t} + v_{NH_4^+} \cdot \Delta t \tag{44}$$

$$\left[HCO_3^-\right]_t = \left[HCO_3^-\right]_{t-\Delta t} + v_{HCO_3^-} \cdot \Delta t \tag{45}$$

Where [NO_3^-], [Na^+], [NH_4^+] and [HCO_3^-] are the ion concentrations in electrolyte, t and Δt are the reaction time and time step, respectively. The $v_{HCO_3^-}$ is yield rate of HCO_3^-.

Step 2: $NaHCO_3$ precipitation

The concentration of saturated $NaHCO_3$ solution is 1.14 mol L^{-1} at ambient condition, thus, the $K_{sp,NaHCO_3}$ of $NaHCO_3$ was calculated to be 1.30. The amount of precipitation was set to be *s*.

$$(\left[Na^+\right]_t - s) \cdot (\left[HCO_3^-\right]_t - s) \geq K_{sp,NaHCO_3} \tag{46}$$

The initial critical condition that has begun to precipitate has been calculated as:

$$s^2 - (\left[Na^+\right]_t + \left[HCO_3^-\right]_t)s + (\left[Na^+\right]_t \cdot \left[HCO_3^-\right]_t - K_{sp,NaHCO_3}) = 0 \tag{47}$$

$$s = \frac{(\left[Na^+\right]_t + \left[HCO_3^-\right]_t) - \sqrt{(\left[Na^+\right]_t + \left[HCO_3^-\right]_t)^2 - 4(\left[Na^+\right]_t \cdot \left[HCO_3^-\right]_t - K_{sp,NaHCO_3})}}{2} \tag{48}$$

Thus, the concentrations of Na^+ and HCO_3^- were obtained.

$$\left[HCO_3^-\right] = \left[HCO_3^-\right]_t - s \tag{40}$$

$$\left[Na^+\right] = \left[Na^+\right]_t - s \tag{50}$$

The concentrations of NH_4^+ and NO_3^- changed as a constant rate, respectively.

It is seen from above figure that the ratio of Na^+/NH_4^+ concentration changes continuously with the elongation of reaction duration. The initial $NaNO_3$ electrolyte contains of NO_3^- (2 mol L^{-1}) and Na^+ (2 mol L^{-1}). With the polarization at 2 A from 0 ~ 15 hours, the concentrations of NO_3^- decrease linearly from 2 mol L^{-1} to 0.6 mol L^{-1}, while that of NH_4^+ increases linearly from 0 mol L^{-1} to 1.4 mol L^{-1}, which is in line with the NO_3^-–to–NH_4^+ conversion.

In great contrast, the concentration of Na^+ remains stable at 2 mol L^{-1} in the early stage from 0 ~ 1.52 hours, and thereafter begins to decrease. It is known that CO_2–to–HCO_3^- conversion occurs during the reaction process, and the $[Na^+]\cdot[HCO_3^-]$ reaches a K_{sp} (1.30); so it is proposed that $NaHCO_3$ begin to precipitate at 1.52 hours. With the elongation of reaction duration, the Na^+ concentration sharply decreases, which is owing to accelerated $NaHCO_3$ solid precipitates with more HCO_3^- formed during this process. Finally, when the Na^+ concentration in the solution decreases to ~ 0.22 mol L^{-1}, the system stops precipitating $NaHCO_3$ (15.5 hours).

Based on the above data, we have plotted the change of Na^+/NH_4^+ concentration ratios with reaction duration. We have corrected all of the electrochemical data according to the reaction durations.

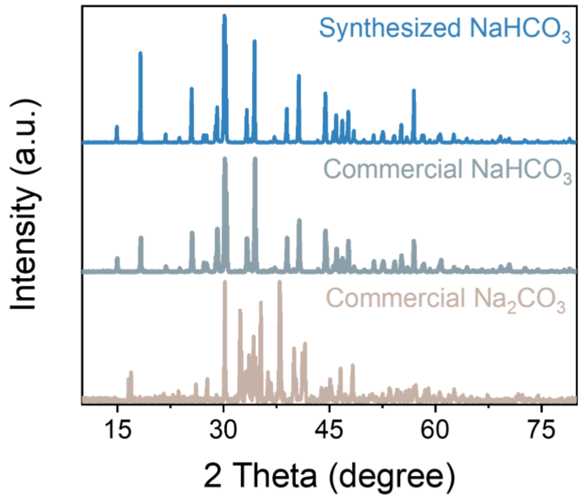

Supplementary Fig. 70. The XRD patterns of as-synthesized $NaHCO_3$, commercial $NaHCO_3$ and commercial Na_2CO_3. Source data is provided as a Source Data file.

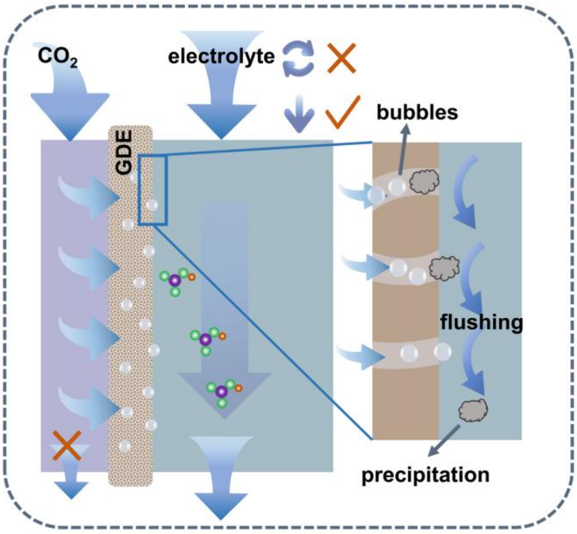

Supplementary Fig. 71. The proposed solutions to mitigate $NaHCO_3$ deposition on electrode surface, especially in long-time operation.

We have proposed the origin of this phenomenon as follows:

Firstly, within the operando-electrified Solvay system, dynamic pressure regulation and gas flow flushing techniques were implemented. This configuration is capable of sustaining a continuous supply of CO_2 during testing, thereby regulating the internal pressure of the gas chamber within the electrolytic cell. Consequently, the CO_2 gas is pumped through GDE and delivered to the electrode surface, thereby effectively flushing away $NaHCO_3$ deposits and averting blockages in gas channels.[25, 26]

Secondly, the electrolyte was circulated in a single-pass mode. It is evident that an electrolyte single-pass flow was adopted in order to prevent excessive accumulation of $NaHCO_3$ concentration, which would otherwise have been caused by multiple cycles. This has enabled precise modulation of the electrolyte environment, thereby attenuating the thermodynamic driving force responsible for the processes of $NaHCO_3$ crystallization and deposition.

In order to verify the aforementioned hypothesis, an attempt was made to characterize the microscopic structure of the electrode material after long-term operation by a scanning electron microscope (SEM; Supplementary Fig. 73). This observation is significant in that it demonstrated the absence of $NaHCO_3$ deposition on the electrode surface, where the material morphology remained nearly unaltered.

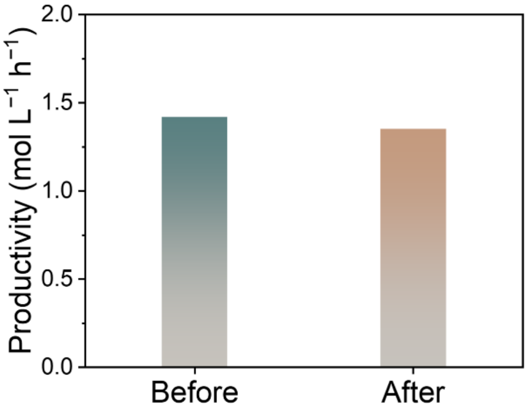

Supplementary Fig. 72. The $NaHCO_3$ productivity of GaOOH/rGO electrode in 10-minute multiple-cycle tests before and after long-duration stability tests. Source data is provided as a Source Data file.

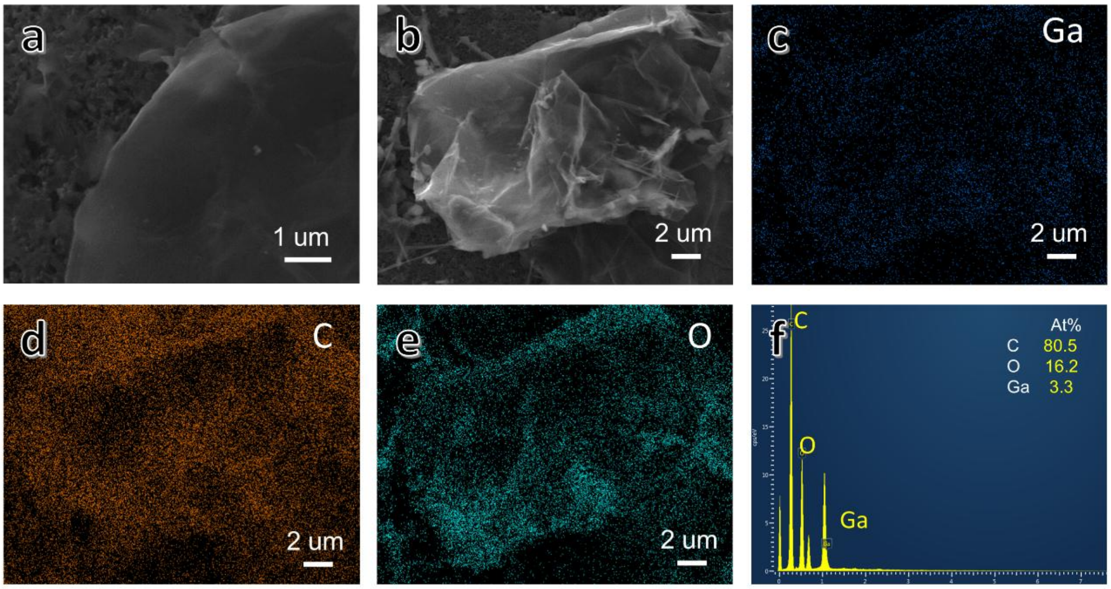

Supplementary Fig. 73 a–b, SEM images of GaOOH/rGO electrode after long-duration stability tests. **c–e**, EDS mapping images of GaOOH/rGO electrode after long-duration stability tests. **f**, Elements content of GaOOH/rGO electrode after long duration stability tests.

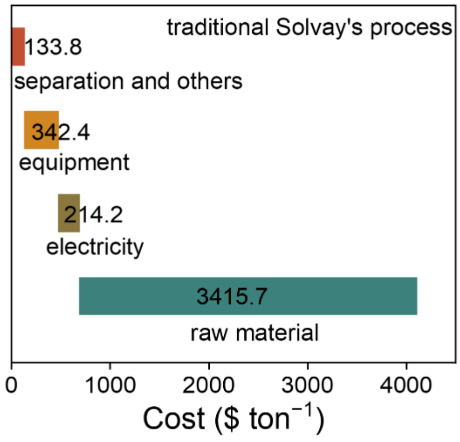

Supplementary Fig. 74. The roadmap to $NaHCO_3$ production cost by successive changes to cost-relevant parameters for traditional Solvay's process. Source data is provided as a Source Data file.

Based on above data, the potential industrialization of *operando*-electrified Solvay process has been discussed.

1) Firstly, the technological breakthrough in system stability is an essential condition. In our *operando*-electrified Solvay system, we have attempted to achieve catalyst and cell design optimization. The optimized GaOOH/rGO catalyst exhibits only ~ 5% decay in $NaHCO_3$ yield after 200 hours of continuous operation at 2A, attributed to its surface pH-swing arising from NO_3RR activity and CO_2RR passivity. Furthermore, the flow-cell configuration suppresses $NaHCO_3$ deposition *via operando* CO_2 flushing. The good system stability is confirmed by post-operation SEM images showing little electrode morphology change (Supplementary Fig. 73).

2) Next, the production cost is another vital criterion for industrialization. In the traditional Solvay process, the ammonia and alkaline as feedstock constitute a substantial proportion of the overall cost.[27]. In this respect, our *operando*-electrified Solvay process represents a significant reduction in the use of expensive performed raw materials. Our cost analysis reveals that the *operando*-electrified Solvay system

has a significantly lower cost than the traditional Solvay process.

3) For promoting further industrialization, our *operando*-electrified Solvay system still needs further improvement. Firstly, the issue of electricity costs must be addressed. The current global distribution of electricity is uneven, and the market for clean electricity has yet to be fully established. It is anticipated that a reduction in electricity costs will result in a period of significant development for our electrified plan. Secondly, the present system is deficient in the realm of large-scale pilot testing and verification. The processes of industrialization and expansion of production are not linear; rather, they are complex and multifaceted, and the engineering applications and process design involved require further exploration.

3. Supplementary Tables

Supplementary Table 1. The comparison with electrochemical production of $NaHCO_3$ in the literature (Reference numbers are marked in Fig. 2i in the main text). [28-34]

Reference number in Fig. 2i of the main text	Method	Raw materials	Productivity	Reference
	Flow cells	2 M $NaNO_3$ +CO_2	374.4 mmol/L/cm^2/h	This work
Ref. 1	Electrodialysis metathesis (EDM)	2 M NaCl+2M $NH_3 \cdot H_2O$ +CO_2	5.95 mmol/L/cm^2/h	Chem. Eng. Sci. 283, 119381 (2024).
Ref. 2	Electrodialysis metathesis (EDM)	0.1 M Na_2SO_4+CO_2	0.296 mmol/L/cm^2/h	Chem. Eng. J. 468, 143686 (2023).
Ref. 3	CO_2-mineralization fuel cell	1 M NaCl+$Ca(OH)_2$+CO_2	1.54 mmol/L/cm^2/h	Sci. China Technol. Sci. 57, 2335-2343 (2014).
Ref. 4	Bipolar membrane electrodialysis	0.5 M NaCl+$NaSO_4$+$MgCl_2$ +KCl+$CaCl_2$+CO_2	0.183 mmol/L/cm^2/h	J. Clean. Prod. 438, 140578 (2024).
Ref. 5	Membrane electrodialysis	2.25 M NaOH, 1.42 M Na_2CO_3	8.125 mmol/L/cm^2/h	Ind. Eng. Chem. Res. 57, 12583-12589 (2018)
Ref. 6	Electrodialysis	0.5M NaCl+CO_2	0.167 mmol/L/cm^2/h	Desalination 520, 115327 (2021).
Ref. 7	Bipolar membrane electrodialysis	0.01M HCl+0.01M NaOH+0.24M $NaNO_3$+seawater	0.104 mmol/L/cm^2/h	Sci. Total Environ. 820, 153272 (2022)

Supplementary Table 2. State-of-the-art literature for electrochemical co-synthesis from CO_2 and nitrate leading to C–N coupling products mainly including urea, and occasionally methylamine and ethylamine.[19, 35-107]

Literature citations	Catalyst	Feedstock	Product	Reference
19	V_O-InOOH	CO_2 + 0.1 M KNO_3	urea	ACS Nano 16, 8213–8222 (2022).
35	Fe@C-Fe_3O_4/CNTs	CO_2 + 0.1 M KNO_3	urea	Angew. Chem. Int. Ed. 62, e202210958 (2023).
36	Ru-Cu_9Bi/CNT	CO_2 + 0.1 M $KHCO_3$ + 0.1 M KNO_3	urea	Energy Environ. Sci. 17, 5183–5190 (2024).
37	graphene-In_2O_3	CO_2 + 0.1 M $KHCO_3$ + 0.1 M KNO_3	urea	Chin. Chem. Lett. 35, 108540 (2024).
38	$CoRuN_6$	CO_2 + 0.1 M KNO_3	urea	Appl. Catal. B: Environ. 336, 122917 (2023).
39	V_O-$In(OH)_3$ NBs	CO_2 + 1.0 M KNO_3	urea	Nano Lett. 24, 11730–11737 (2024).
40	Cu_1/In_2O_3	CO_2 + 0.1 M $KHCO_3$ + 0.1 M KNO_3	urea	ACS Nano 18, 25316-25324 (2024).
41	N-doped carbon	CO_2 + 0.1 M $KHCO_3$ + 10 mM KNO_3	urea	Carbon Energy 5, e345 (2023).
42	SAC Cu-TiO_2	CO_2 + 0.1 M $KHCO_3$ + 100 ppm KNO_3	urea	Appl. Catal. B: Environ. 338, 123056 (2023).
43	PdZn/C	CO_2 + 0.1 M KNO_3 + 0.2 M $KHCO_3$	urea	Nano-Micro Lett. 16, 247 (2024).
44	Fe_1/MoS_2	CO_2 + 0.1 M $KHCO_3$ + 0.1 M KNO_3	urea	ACS Nano 18, 27718–27726 (2024).
45	FeNC-Fe_1N_4	CO_2 + 0.1 M $KHCO_3$ + 0.1 M KNO_3	urea	Small 20, 2400036 (2024).

46	CuOxZnOy	CO_2 + 0.1 M Na_2SO_4 + 0.1 M $NaNO_3$	urea	Commun. Chem. 6, 199 (2023).
47	F-CNT	CO_2 + 0.1 M KNO_3	urea	Appl. Catal. B: Environ. 316, 121618 (2022).
48	MoOx/C	CO_2 + 0.1 M KNO_3	urea	Angew. Chem. Int. Ed. 62, e202301957 (2023).
49	Cu electrode	CO_2 + 0.1 M $KHCO_3$ and 50 mM KNO_3	urea	Appl. Catal. B: Environ. 316, 121512 (2022).
50	$Pd_{70}Cu_{12}Bi_{18}$	CO_2 + 0.1 M KNO_3	urea	Chin. J. Catal. 63, 270–281 (2024).
51	Cu_2O	CO_2 + 1 M KOH + 0.1 M KNO_3	urea	Energy Environ. Sci. 16, 2003–2013 (2023).
52	In_2O_3-NT	CO_2 + 0.1 M KNO_3	urea	Green Chem. 26, 6812–6821 (2024).
53	Cu-CeO_2	CO_2 + 0.1 M $KHCO_3$ + 50 mM KNO_3	urea	Adv. Mater. 35, 2300020 (2023).
54	FeNi@nC-1000	CO_2 + 0.1 M KNO_3	urea	Chin. J. Catal. 65, 153–162 (2024).
55	Co NPs@C	CO_2 + 0.2 M $NaHCO_3$ + 0.05 M $NaNO_3$	urea	Adv. Mater. 36, 2401221 (2024).
56	In_4SnS_8	CO_2 + 0.1 M KNO_3	urea	Inorg. Chem. Front. 11, 6010–6019 (2024).
57	$CuWO_4$	CO_2 + 0.1 M KNO_3	urea	Nat. Commun. 14, 4491 (2023).
58	a-TiS_2	CO_2 + 0.1 M KNO_3 + 0.1 M $KHCO_3$	urea	J. Colloid Interf. Sci. 679, 60-66 (2025).
59	W800-Cu	CO_2 + 0.1 M KNO_3 + 0.1 M $KHCO_3$	urea	AIChE J. 70, e18515 (2024).
60	TiO_2-Nafion	CO_2 + 0.1 M KNO_3	urea	ChemSusChem 10, 3999–4003 (2017).
61	Ru_1Co	CO_2 + 0.1 M KNO_3 + 0.1 M $KHCO_3$	urea	Adv. Funct. Mater. 34, 2406438 (2024).
62	VB_{12}-CNTs	CO_2 + 0.1 M KNO_3	urea	Appl. Catal. B: Environ. 351, 123941 (2024).

63	Cu	CO_2 + 0.02 M KNO_3 + 0.2 M $KHCO_3$	urea	J. Electroanal. Chem. 387, 143–145 (1995).
64	Cu/PI-500	CO_2 + 0.1 M KNO_3 + 0.1 M $KHCO_3$	urea	Angew. Chem. Int. Ed. e202410938 (2024).
65	Fe^{II}-Fe^{III}OOH@BiVO_4-n	CO_2 + 0.1 M KNO_3	urea	J. Energy Chem. 84, 385–393 (2023).
66	γ-Fe_2O_3	CO_2 + 1 M $KHCO_3$ + 0.1 M KNO_3	urea	Nat. Synth. 3, 1404–1413 (2024).
67	Fe-doped InOOH	CO_2 + 0.1 M KNO_3	urea	Inorg. Chem. 63, 20935–20939 (2024).
68	$CuInS_2$/TF	CO_2 + 0.05 M KNO_3 + 0.05 M $KHCO_3$	urea	Sci. Total Environ. 913, 169722 (2024).
69	Fe_1Cu_3-C/NC	CO_2 + 0.1 M KNO_3	urea	J. Colloid Interf. Sci. 674, 834–840 (2024).
70	FeNi/NC	CO_2 + 0.1 M KNO_3	urea	Mater. Chem. Front. 7, 4952–4960 (2023).
71	Ti-DHTP	CO_2 + 0.1 M KNO_3 + 0.5 M K_2SO_4	urea	Adv. Funct. Mater. 34, 2400892 (2024).
72	PdCu/CBC	CO_2 + 0.05 M KNO_3	urea	EES Catal. 1, 45–53 (2023).
73	TiN_X	CO_2 + 0.1 M KNO_3	urea	Chem. Eng. J. 486, 150178 (2024).
74	PcNi-Fe-O	CO_2 + 0.1 M KNO_3	urea	Angew. Chem. Int. Ed. 63, e202410625 (2024).
75	B-FeNi-DASC	CO_2 + 0.1 M $KHCO_3$ + 50 mM KNO_3	urea	Nat. Commun. 13, 5337 (2022).
76	CuPc-Amino	CO_2 + 0.1 M $KHCO_3$ + 0.05 M KNO_3	urea	Nat. Commun. 15, 8858 (2024).
77	CoPc-COF@TiO_2 NTs	CO_2 + 0.3 M $KHCO_3$ + 0.2 M KNO_3	urea	Sci. China Chem. 66, 1417–1424 (2023).
78	Zr-Fe/CN	CO_2 + 0.1 M KNO_3	urea	Water Res. 266, 122317 (2024).

79	Cu@Zn nanowires	CO_2 + 0.2 M $KHCO_3$ + 0.1 M KNO_3	urea	ACS Nano 16, 9095–9104 (2022).
80	Vo-CeO_2	CO_2 + 0.1 M $KHCO_3$ + 50 mM KNO_3	urea	J. Am. Chem. Soc. 144, 11530–11535 (2022).
81	Cu_2O	CO_2 + 0.1 M KNO_3	urea	Angew. Chem. Int. Ed. 63, e202406515 (2024).
82	NiFe NC-PNHA	CO_2 + 0.5 M $KHCO_3$ + 0.02 M KNO_3	urea	Chem. Eng. J. 492, 152161 (2024).
83	N-PHCS	CO_2 + 0.1 M $KHCO_3$ + 0.05 M KNO_3	urea	ACS Appl. Mater. Interfaces 16, 50726–50735 (2024).
84	Fe-TPP/CNTs	CO_2 + 0.2 M $KHCO_3$ + 0.1 M KNO_3	urea	Nat. Sustain. 7, 442–451 (2024).
85	GaN nanowires	CO_2 + 0.5 M KNO_3	urea	ACS Catal. 14, 2588–2596 (2024).
86	XC72R-AuPd	CO_2 + 0.075 M $KHCO_3$ + 0.025 M KNO_3	urea	Appl. Catal. B: Environ. 318, 121819 (2022).
87	Rh_1Cu	CO_2 + 0.1 M KNO_3 + 0.1 M $KHCO_3$	urea	ACS Nano 18, 29856–29863 (2024).
88	Ag GDE	CO_2 + 0.1 M KNO_3 + 0.1 M $KHCO_3$	urea	Sustain. Energ. Fuels, (2024).
89	$In(OH)_3$-S	CO_2 + 0.1 M KNO_3	urea	Nat. Sustain. 4, 868–876 (2021).
90	Zn/Cu	CO_2 + 500 ppm KNO_3 + 0.1 M $KHCO_3$	urea	Nat. Catal. 6, 939–948 (2023).
91	Ru/Pt/Pd-Cu CF	CO_2 + 0.1 M $NaNO_3$	urea	ACS Sustain. Chem. Eng. 10, 15869–15875 (2022).
92	Cu_1Ru	CO_2 + 0.1 M KNO_3 + 0.1 M $KHCO_3$	urea	Acs Energy Lett. 9, 4624–4632 (2024).
93	nitrogen-doped carbon	CO_2 + 0.1 M KNO_3 + 0.1 M $KHCO_3$	urea	Nat. Commun. 15, 176 (2024).

94	Cu/ZnO	CO_2 + 0.1 M KNO_3	urea	ACS Energy Lett. 8, 3373–3380 (2023).
95	Ag-CuNi(OH)$_2$	CO_2 + 0.1 M KNO_3 + 0.1 M $KHCO_3$	urea	Angew. Chem. Int. Ed. 63, e202410105 (2024).
96	Cu-SP-OMe	CO_2 + 0.01 M KNO_3 + 0.1 M $KHCO_3$	urea	Angew. Chem. Int. Ed. 62, e202307123 (2023).
97	Cu_1Mo_1/NC	CO_2 + 0.1 M KNO_3 + 0.1 M $KHCO_3$	urea	Chem. Eng. J. 497, 154455 (2024).
98	Vo-S-IO-6	CO_2 + 0.1 M KNO_3	urea	Appl. Catal. B: Environ. 338, 122962 (2023).
99	TiO_2-C	CO_2 + 0.1 M KNO_3	urea	Angew. Chem. Int. Ed. 63, e202403980 (2024).
100	a-$Cu_{0.1}CoB_X$ metallene	CO_2 + 0.1 M KNO_3	urea	Small 2407679 (2024).
101	Cu-GS-800	CO_2 + 0.1 M KNO_3 + 0.1 M $KHCO_3$	urea	Adv. Energy Mater. 12, 2201500 (2022).
102	$CuPd_1Rh_1$-DAA	CO_2 + 0.1 M KNO_3 + 0.1 M $KHCO_3$	urea	Adv. Mater. 36, 2402160 (2024).
103	Cu-Bi	CO_2 + 0.1 M KNO_3 + 0.2 M $KHCO_3$	urea	J. Am. Chem. Soc. 146, 25813–25823 (2024).
104	Cu-HATNA	CO_2 + 0.1 M KNO_3 + 0.1 M $KHCO_3$	urea	Chem. Commun. 60, 3669–3672 (2024).
105	RP-CuAu	CO_2 + 0.1 M $KHCO_3$ + 0.1 M KNO_3	urea	J. Am. Chem. Soc. 147, 8871–8880 (2025).
106	Cu catalyst	CO_2 + 0.1 M KNO_3 + 1.0 M $KHCO_3$	ethylamine	J. Energy Chem. 65, 367–370 (2022).
107	CoPc-NH_2/CNT	CO_2 +0.1 M $KHCO_3$ + 0.5 M KNO_3	Methylamine	Nat. Sustain. 4, 725–730 (2021).

Supplementary Table 3. The comparison of ECSA, EIS, productivity, energy consumption and reaction onset potentials for different samples.

Properties \ Sample	GaOOH/rGO	GaOOH	rGO
EIS /Ω	19.9	70.1	41.5
ECSA / mF cm^{-2}	9.48	3.14	6.35
Productivity / mmol L^{-1} h^{-1} cm^{-2}	293.0	228.3	218.8
Energy consumption / kg kWh^{-1}	0.582	0.809	1.038
Onset potential / V vs. RHE	−0.17	−0.29	−0.39

Supplementary Table 4. EXAFS fitting for Ga foil, Ga_2O_3 and GaOOH/rGO

Material	shell	CN	R(Å)	ΔE_0(eV)	σ^2($Å^2$)	R factor
Ga-foil	Ga–Ga	4.000	2.71749	4.073	0.01758	0.0115517
Ga_2O_3	Ga–O	3.007	1.87316	−7.422	0.01430	0.0144739
	Ga–Ga	3.001	3.28814	−7.422	0.00172	
	Ga–Ga	2.004	3.43283	−7.422	0.01034	
GaOOH/rGO	Ga–O	2.491	1.94762	−1.388	0.01131	0.0042390
	Ga–Ga	3.122	2.97437	−1.388	0.00914	

NOTE: CN: coordination number; R: bond lengths between central atoms and surrounding coordination atoms; σ^2: Debye-Waller factor to account for both thermal and structural disorders; ΔE_0: the difference of the zero kinetic energy value between the sample and theoretical model; R factor is used to measure the goodness of the fitting.

Supplementary Table 5. The comparison with chemical production of $NaHCO_3$ in the literature (Reference numbers are marked in Fig. 4f in the main text). [108-111]

Reference number in Fig. 4f of the main text	Method	Raw materials	Productivity	Reference
	Scale-up prototype model cell	2 M $NaNO_3$ +CO_2	3.63 mol L^{-1} h^{-1}	This work
Ref. 8	Modified Solvay process	1.2 M NaCl + CO_2 + KOH	0.18 mol L^{-1} h^{-1}	Crystals 13, 470 (2023).
Ref. 9	Modified Solvay process	1.2 M NaCl + CO_2 +0.6 M NH_4HCO_3	0.21 mol L^{-1} h^{-1}	Crystals 13, 470 (2023).
Ref. 10	Modified Solvay process	1.2 M NaCl + CO_2 +1.2 M NH_4HCO_3	0.25 mol L^{-1} h^{-1}	Crystals 13, 470 (2023).
Ref. 11	Modified Hou's process	0.97 M NaCl + CO_2 +NH_3	0.32 mol L^{-1} h^{-1}	Benyahia, F. Carbon dioxide mineralization using reject brine (2016).
Ref. 12	Modified Hou's process	1.45 M NaCl + CO_2 +NH_3	0.48 mol L^{-1} h^{-1}	Benyahia, F. Carbon dioxide mineralization using reject brine (2016).
Ref. 13	Modified Hou's process	1.93 M NaCl + CO_2 +NH_3	0.60 mol L^{-1} h^{-1}	Benyahia, F. Carbon dioxide mineralization using reject brine (2016).
Ref. 14	Brine treatment and CO_2 capture	0.5 M NaCl+0.75 M NaOH +CO_2	0.004 mol L^{-1} h^{-1}	Desalination 442, 62–74 (2018).
Ref. 15	Brine treatment and CO_2 capture	0.96 M NaCl+1.44 M NaOH +CO_2	0.01 mol L^{-1} h^{-1}	Desalination 442, 62–74 (2018).
Ref. 16	Na_2SO_4 waste and CO_2 capture	3.52 M Na_2SO_4+CO_2+20% Na_2CO_3+NH_3	0.79 mol L^{-1} h^{-1}	Korean J. Chem. Eng. 41, 2163–2172 (2024).

Supplementary Table 6. The comparisons of reaction rates, CO_2 selectivity and energy efficiency between our *operando*-electrified system and the traditional Solvay method.[111-113]

Methods	Reaction rates / mol L^{-1} h^{-1}	CO_2 selectivity / %	Energy efficiency / ton $NaHCO_3$ GJ^{-1}
operando-electrified system	3.63	~ 70	0.16
traditional Solvay process	0.79 (Korean J. Chem. Eng. 41, 2163–2172 (2024).)	~ 83 (J. Clean. Prod. 468, 143087 (2024).)	0.10 (Ind. Eng. Chem. Res. 58, 3450–3458 (2019).)

The conversion rate of CO_2 (or CO_2 selectivity) is 70% under the conditions of 500 mA cm^{-2} & 10 mL min^{-1} CO_2, which is calculated as follows:

$$CO_2 \text{ conversion rate} = \frac{C \times V}{r \times t/V_m} \quad (51)$$

Where C represents the production concentration in the electrolyte, V means the volume of electrolyte, r and t refer to gas flow rate and reaction time, respectively. And V_m is the molar volume of gas (22.4 L mol^{-1} under standard conditions). According to the above conditions, the values of C, V, r and t are 374.4 mmol L^{-1}, 50 mL, 10 mL min^{-1} and 1 hour, respectively. The final conversion rate of CO_2 is calculated to be 70%.

Supplementary Table 7. The solubility of $NaNO_3$, NH_4HCO_3, NH_4NO_3 and $NaHCO_3$.[114]

solute	solubility g / 100 g H_2O (20°C)
$NaNO_3$	87.2
NH_4HCO_3	21.7
NH_4NO_3	101.7
$NaHCO_3$	9.6

Reference

1. Haegel, N. M., et al. Terawatt-scale photovoltaics: Trajectories and challenges. *Science* **356**, 141–143 (2017).
2. Jouny, M., Luc, W. & Jiao, F. General techno-economic analysis of CO_2 electrolysis systems. *Ind. Eng. Chem. Res.* **57**, 2165–2177 (2018).
3. Chinabgao, *Price of sodium nitrate* (2023); https://www.chinabgao.com/jiage/8795181.html.
4. Hangyan, *Price of carbon dioxide* (2022); https://www.hangyan.co/charts/2953735139055634387.
5. Shin, H., Hansen, K. U. & Jiao, F. Techno-economic assessment of low-temperature carbon dioxide electrolysis. *Nat. Sustain.* **4**, 911–919 (2021).
6. Zhang, X., et al. Electrochemical regeneration of high-purity CO_2 from (bi)carbonates in a porous solid electrolyte reactor for efficient carbon capture. *Nat. Energy* **10**, 55–65 (2024).
7. Na, J., et al. General technoeconomic analysis for electrochemical coproduction coupling carbon dioxide reduction with organic oxidation. *Nat. Commun.* **10**, 5193 (2019).
8. Lee, J. H., Lee, J. H., Park, I. K. & Lee, C. H. Techno-economic and environmental evaluation of CO_2 mineralization technology based on bench-scale experiments. *J. CO_2 Util.* **26**, 522–536 (2018).
9. Chinabgao, *Price of sodium chloride* (2025); https://www.chinabgao.com/jiage/lvhuana/.
10. Chyxx, *Price of ammonia* (2024); https://www.chyxx.com/shuju/1175093.html.
11. Smm, *Price of quick lime* (2024); https://www.smm.cn/mpdb/1705462053488_price_china.
12. Zhu, X., et al. Modulating Ru–Co bond lengths in Ru_1Co single-atom alloys through crystal phase engineering for electrocatalytic nitrate–to–ammonia conversion. *Nat. Commun.* **16**, 5742 (2025).
13. Zhi, Q. J., et al. Dithiine-linked metalphthalocyanine framework with undulated layers for highly efficient and stable H_2O_2 electroproduction. *Nat. Commun.* **15**, 678 (2024).

14. Lin, Y. X., et al. Optimizing surface active sites via burying single atom into subsurface lattice for boosted methanol electrooxidation. *Nat. Commun.* **16**, 286 (2025).
15. Basak, H. K., Adak, M. K., Rajput, A. & Chakraborty, B. Low Pt loading on wolframite-type $NiWO_4$ to excel the electrocatalytic water splitting and ammonia oxidation reaction. *ACS Appl. Mater. Interfaces* **17**, 9391–9406 (2025).
16. Yamaguchi, S., Ebe, H., Minegishi, T. & Sugiyama, M. Introduction of a conductive layer into flood-resistant gas diffusion electrodes with polymer substrate for an efficient electrochemical CO_2 reduction with copper oxide. *ACS Appl. Mater. Interfaces* **16**, 17371–17376 (2024).
17. Zatsepin, D. A., et al. Atomic structure, electronic states, and optical properties of epitaxially grown β–Ga_2O_3 layers. *Superlattices and Microstruct.* **120**, 90–100 (2018).
18. Lertanantawong, B., Riches, J. D. & O'mullane, A. P. Room temperature electrochemical synthesis of crystalline GaOOH nanoparticles from expanding liquid metals. *Langmuir* **34**, 7604–7611 (2018).
19. Lv, C., et al. A defect engineered electrocatalyst that promotes high-efficiency urea synthesis under ambient conditions. *ACS Nano* **16**, 8213–8222 (2022).
20. Yang, C. F., et al. Electrochemically reconstructed Cu–FeOOH/Fe_3O_4 catalyst for efficient hydrogen evolution in alkaline media. *Adv. Energy Mater.* **12**, 2200077 (2022).
21. Ye, F., et al. The role of oxygen-vacancy in bifunctional indium oxyhydroxide catalysts for electrochemical coupling of biomass valorization with CO_2 conversion. *Nat. Commun.* **14**, 2040 (2023).
22. Chang, H.-W., et al. X-ray absorption spectroscopic study on interfacial electronic properties of FeOOH/reduced graphene oxide for asymmetric supercapacitors. *ACS Sustain. Chem. Eng.* **5**, 3186–3194 (2017).
23. Genovese, C., et al. Operando spectroscopy study of the carbon dioxide electro-reduction by iron species on nitrogen-doped carbon. *Nat. Commun.* **9**, 935 (2018).

24. Zhu, L., et al. Composite of CoOOH nanoplates with multiwalled carbon nanotubes as superior cathode material for supercapacitors. *J. Phys. Chem. C* **119**, 7069–7075 (2015).

25. Liang, J., et al. Efficient bubble/precipitate traffic enables stable seawater reduction electrocatalysis at industrial-level current densities. *Nat. Commun.* **15**, 2950 (2024).

26. Liang, J., et al. Electroreduction of alkaline/natural seawater: Self-cleaning Pt/carbon cathode and on-site co-synthesis of H_2 and Mg hydroxide nanoflakes. *Chem* **10**, 3067–3087 (2024).

27. Rahimi-Ahar, Z. & Ghareghashi, A. Methods and current developments of the Solvay process for brine management and CO_2 capture: A critical review. *Environ. Chem.* **21**, 1–14 (2024).

28. Bi, J. T., et al. A continuous electrodialysis metathesis integrated with in-situ CO_2 utilization for controllable $NaHCO_3/NH_4Cl$ or Na_2CO_3/NH_4Cl production from NaCl and $NH_3 \cdot H_2O$. *Chem. Eng. Sci.* **283**, 119381 (2024).

29. Chen, T. Y., et al. Carbon dioxide capture coupled with magnesium utilization from seawater by bipolar membrane electrodialysis. *Sci. Total Environ.* **820**, 153272 (2022).

30. Mustafa, J., Al-Marzouqi, A. H., El-Naas, M. H. & Ghasem, N. Electrodialysis based waste utilization methodology for the desalination industry. *Desalination* **520**, 115327 (2021).

31. Mustafa, J., et al. Synergistic approach for carbon dioxide capture and reject brine treatment: Integrating selective electrodialysis and bipolar membrane electrodialysis. *J. Clean. Prod.* **438**, 140578 (2024).

32. Wang, H. Y., et al. A direct electrochemical substitution electrodialytic system for CO_2 conversion into high value-added soda. *Chem. Eng. J.* **468**, 143686 (2023).

33. Wang, M. Y., Liu, L. & Wang, Z. Direct electrodeposition of Ga and the simultaneous production of NaOH and $NaHCO_3$ from carbonated spent liquor by membrane electrolysis. *Ind. Eng. Chem. Res.* **57**, 12583–12589 (2018).

34. Xie, H. P., et al. Generation of electricity from CO_2 mineralization: Principle and realization. *Sci. China Technol. Sci.* **57**, 2335–2343 (2014).

35. Geng, J., et al. Ambient electrosynthesis of urea with nitrate and carbon dioxide over iron-based dual-sites. *Angew. Chem. Int. Ed.* **62**, e202210958 (2023).

36. Yu, Y. D., et al. Achieving efficient urea electrosynthesis through improving the coverage of a crucial intermediate across a broad range of nitrate concentrations. *Energy Environ. Sci.* **17**, 5183–5190 (2024).

37. Mao, Y. N., et al. Ambient electrocatalytic synthesis of urea by co-reduction of NO_3^- and CO_2 over graphene-supported In_2O_3. *Chin. Chem. Lett.* **35**, 108540 (2024).

38. Liu, C. C., et al. The asymmetric orbital hybridization in single-atom-dimers for urea synthesis by optimizing the C–N coupling reaction pathway. *Appl. Catal. B: Environ.* **336**, 122917 (2023).

39. Xu, Z. F., et al. Atomic defects engineering boosts urea synthesis toward carbon dioxide and nitrate coelectroreduction. *Nano Lett.* **24**, 11730–11737 (2024).

40. Zhang, Y., et al. Atomically dispersed Cu on In_2O_3 for relay electrocatalytic conversion of nitrate and CO_2 to urea. *ACS Nano* **18**, 25316–25324 (2024).

41. Chen, C., et al. Balancing sub-reaction activity to boost electrocatalytic urea synthesis using a metal-free electrocatalyst. *Carbon Energy* **5**, e345 (2023).

42. Zheng, J. G., et al. Boosting efficient C–N bonding toward photoelectrocatalytic urea synthesis from CO_2 and nitrate via close Cu/Ti bimetallic sites. *Appl. Catal. B: Environ.* **338**, 123056 (2023).

43. Zhou, W. L., et al. Boosting electrochemical urea synthesis via constructing ordered Pd–Zn active pair. *Nano-Micro Lett.* **16**, 247 (2024).

44. Du, W. Y., et al. Boosting electroreduction of nitrate and CO_2 to urea on a tandem Fe_1/MoS_2 catalyst. *ACS Nano* **18**, 27718–27726 (2024).

45. Li, Z. W., et al. Boosting up electrosynthesis of urea with nitrate and carbon dioxide via synergistic effect of metallic iron cluster and single-atom. *Small* **20**, 2400036 (2024).

46. Anastasiadou, D., et al. Carbon dioxide and nitrate co-electroreduction to urea on CuO_xZnO_y. *Commun. Chem.* **6**, 199 (2023).

47. Liu, X. W., et al. Carbon nanotubes with fluorine-rich surface as metal-free electrocatalyst for effective synthesis of urea from nitrate and CO_2. *Appl. Catal. B: Environ.* **316**, 121618 (2022).
48. Sun, M. M., et al. Carbon-anchored molybdenum oxide nanoclusters as efficient catalysts for the electrosynthesis of ammonia and urea. *Angew. Chem. Int. Ed.* **62**, e202301957 (2023).
49. Krzywda, P. M., et al. Carbon–nitrogen bond formation on Cu electrodes during CO_2 reduction in NO_3^- solution. *Appl. Catal. B: Environ.* **316**, 121512 (2022).
50. Zhou, S. L., et al. Continuous-flow electrosynthesis of urea and oxalic acid by CO_2–nitrate reduction and glycerol oxidation. *Chin. J. Catal.* **63**, 270–281 (2024).
51. Shin, S., et al. Copper with an atomic-scale spacing for efficient electrocatalytic co-reduction of carbon dioxide and nitrate to urea. *Energy Environ. Sci.* **16**, 2003–2013 (2023).
52. Fang, H. J., et al. Durable and efficient urea electrosynthesis using carbon dioxide and nitrate over defect-rich In_2O_3 nanotubes. *Green Chem.* **26**, 6812–6821 (2024).
53. Wei, X. X., et al. Dynamic reconstitution between copper single atoms and clusters for electrocatalytic urea synthesis. *Adv. Mater.* **35**, 2300020 (2023).
54. Sun, Z.-S., et al. Efficient electrocatalytic urea synthesis from CO_2 and nitrate over the scale-up produced FeNi alloy-decorated nanoporous carbon. *Chin. J. Catal.* **65**, 153–162 (2024).
55. Fan, X. Y., et al. Efficient electrochemical co-reduction of carbon dioxide and nitrate to urea with high faradaic efficiency on cobalt-based dual-sites. *Adv. Mater.* **36**, 2401221 (2024).
56. Li, M., et al. Efficient electrosynthesis of urea using CO_2 and nitrate over a bifunctional In_4SnS_8 catalyst. *Inorg. Chem. Front.* **11**, 6010–6019 (2024).
57. Zhao, Y. L., et al. Efficient urea electrosynthesis from carbon dioxide and nitrate via alternating Cu–W bimetallic C–N coupling sites. *Nat. Commun.* **14**, 4491 (2023).
58. Yuan, D., et al. Efficient urea electrosynthesis from CO_2 and nitrate on amorphous TiS_2. *J. Colloid Interf. Sci.* **679**, 60–66 (2025).

59. Chen, S. B., et al. Efficient urea electrosynthesis via coordination of the reaction rate of carbon dioxide and nitrate co-reduction. *AIChE J.* **70**, e18515 (2024).

60. Saravanakumar, D., et al. Electrocatalytic conversion of carbon dioxide and nitrate ions to urea by a titania-nafion composite electrode. *ChemSusChem* **10**, 3999–4003 (2017).

61. Wan, Y. Y., et al. Electrocatalytic urea production with nitrate and CO_2 on a Ru-dispersed Co catalyst. *Adv. Funct. Mater.* **34**, 2406438 (2024).

62. Cong, M. Y., et al. Electrocatalytic urea synthesis from CO_2 and nitrate co-reduction on natural vitamin B_{12} coupled carbon nanotubes. *Appl. Catal. B: Environ.* **351**, 123941 (2024).

63. Shibata, M., Yoshida, K. & Furuya, N. Electrochemical synthesis of urea on reduction of carbon dioxide with nitrate and nitrite ions using Cu-loaded gas-diffusion electrode. *J. Electroanal. Chem.* **387**, 143–145 (1995).

64. Wang, Y. J., et al. Electron deficiency is more important than conductivity in C–N coupling for electrocatalytic urea synthesis. *Angew. Chem. Int. Ed.*, e202410938 (2024).

65. Yin, H.-Q., et al. Electrochemical urea synthesis by co-reduction of CO_2 and nitrate with Fe^{II}–$Fe^{III}OOH@BiVO_4$ heterostructures. *J. Energy Chem.* **84**, 385–393 (2023).

66. Huang, D.-S., et al. Electrosynthesis of urea by using Fe_2O_3 nanoparticles encapsulated in a conductive metal-organic framework. *Nat. Synth.* **3**, 1404–1413 (2024).

67. Cai, H. X., et al. Enhanced electrocatalytic urea synthesis over iron-doped InOOH nanosheets under ambient conditions. *Inorg. Chem.* **63**, 20935–20939 (2024).

68. Chen, Y., et al. Exploration of a novel electrochemical CN coupling process: Urea synthesis from direct air carbon capture with nitrate wastewater. *Sci. Total Environ.* **913**, 169722 (2024).

69. Hou, T., et al. FeCu bimetallic clusters for efficient urea production via coupling reduction of carbon dioxide and nitrate. *J. Colloid Interf. Sci.* **674**, 834–840 (2024).

70. Hou, T., et al. $FeNi_3$ nanoparticles for electrocatalytic synthesis of urea from carbon dioxide and nitrate. *Mater. Chem. Front.* **7**, 4952–4960 (2023).

71. Liu, X. F., et al. High C-selectivity for urea synthesis through O-philic adsorption to form *OCO intermediate on Ti-MOF based electrocatalysts. *Adv. Funct. Mater.* **34**, 2400892 (2024).

72. Zhang, S. B., et al. High-efficiency electrosynthesis of urea over bacterial cellulose regulated Pd–Cu bimetallic catalyst. *EES Catal.* **1**, 45–53 (2023).

73. Fang, H. J., et al. Highly efficient and selective electrosynthesis of urea via co-reduction of carbon dioxide and nitrate over the TiN_x catalyst. *Chem. Eng. J.* **486**, 150178 (2024).

74. Qiu, X.-F., et al. Highly efficient electrosynthesis of urea from CO_2 and nitrate by a metal-organic framework with dual active sites. *Angew. Chem. Int. Ed.* **63**, e202410625 (2024).

75. Zhang, X. R., et al. Identifying and tailoring C–N coupling site for efficient urea synthesis over diatomic Fe–Ni catalyst. *Nat. Commun.* **13**, 5337 (2022).

76. Li, H., et al. Ligand engineering towards electrocatalytic urea synthesis on a molecular catalyst. *Nat. Commun.* **15**, 8858 (2024).

77. Li, N., et al. Metalphthalocyanine frameworks grown on TiO_2 nanotubes for synergistically and efficiently electrocatalyzing urea production from CO_2 and nitrate. *Sci. China Chem.* **66**, 1417–1424 (2023).

78. Liu, X. J., et al. Multi-win situation of wastewater purification, carbon emission reduction and resource utilization: Conversion of refractory organics and nitrate to urea and ammonia in a flow-through electrochemical integrated system. *Water Res.* **266**, 122317 (2024).

79. Meng, N. N., et al. Oxide-derived core-shell Cu@Zn nanowires for urea electrosynthesis from carbon dioxide and nitrate in water. *ACS Nano* **16**, 9095–9104 (2022).

80. Wei, X. X., et al. Oxygen vacancy-mediated selective C–N coupling toward electrocatalytic urea synthesis. *J. Am. Chem. Soc.* **144**, 11530–11535 (2022).

81. Li, M., et al. Photoelectrocatalytic synthesis of urea from carbon dioxide and nitrate over a Cu_2O photocathode. *Angew. Chem. Int. Ed.* **63**, e202406515 (2024).

82. Wang, Z. C., et al. Polycation-functionalized interface enable in situ capturing of CO_2 and filtering of proton for efficient C–N coupling toward highly selective urea electrosynthesis. *Chem. Eng. J.* **492**, 152161 (2024).
83. Gao, W. T., et al. Promoting electrocatalytic reduction of CO_2 and nitrate to urea on N-doped porous hollow carbon spheres. *ACS Appl. Mater. Interfaces* **16**, 50726–50735 (2024).
84. Hu, Q., et al. Pulsed co-electrolysis of carbon dioxide and nitrate for sustainable urea synthesis. *Nat. Sustain.* **7**, 442–451 (2024).
85. Dong, W. J., et al. Photoelectrochemical urea synthesis from nitrate and carbon dioxide on GaN nanowires. *ACS Catal.* **14**, 2588–2596 (2024).
86. Wang, H., et al. Realizing efficient C–N coupling via electrochemical co-reduction of CO_2 and NO_3^- on AuPd nanoalloy to form urea: Key C–N coupling intermediates. *Appl. Catal. B: Environ.* **318**, 121819 (2022).
87. Xiang, J. Q., et al. Relay catalysis of isolated rhodium-alloyed copper boosts urea electrosynthesis from nitrate and CO_2. *ACS Nano* **18**, 29856–29863 (2024).
88. Kani, N. C., et al. Screening transition metal electrodes for achieving near 100% selectivity to urea via electroreduction of NO_3^- and CO_2 at 100 mA cm^{-2} current density. *Sustain. Energ. Fuels*, Advance Article (2024).
89. Lv, C., et al. Selective electrocatalytic synthesis of urea with nitrate and carbon dioxide. *Nat. Sustain.* **4**, 868–876 (2021).
90. Luo, Y. T., et al. Selective electrochemical synthesis of urea from nitrate and CO_2 via relay catalysis on hybrid catalysts. *Nat. Catal.* **6**, 939–948 (2023).
91. Qin, J. Z., et al. Selective electrochemical urea synthesis from nitrate and CO_2 using in situ Ru anchoring onto a three-dimensional copper electrode. *ACS Sustain. Chem. Eng.* **10**, 15869–15875 (2022).
92. Wang, F. Z., et al. Selective urea electrosynthesis from nitrate and CO_2 on isolated copper alloyed ruthenium. *ACS Energy Lett.* **9**, 4624–4632 (2024).
93. Li, Y., et al. Sequential co-reduction of nitrate and carbon dioxide enables selective urea electrosynthesis. *Nat. Commun.* **15**, 176 (2024).

94. Wang, Y., et al. Spatial management of CO diffusion on tandem electrode promotes NH_2 intermediate formation for efficient urea electrosynthesis. *ACS Energy Lett.* **8**, 3373–3380 (2023).

95. Ye, W., et al. A strongly coupled metal/hydroxide heterostructure cascades carbon dioxide and nitrate reduction reactions toward efficient urea electrosynthesis. *Angew. Chem. Int. Ed.* **63**, e202410105 (2024).

96. Zhao, Q. L., et al. Sustainable and high-rate electrosynthesis of nitrogen fertilizer. *Angew. Chem. Int. Ed.* **62**, e202307123 (2023).

97. Wang, H., et al. A sustainable approach: Repurposing harmful algal biomass as carbon-based catalysts for nitrogen fertilizer electrosynthesis from nitrate and CO_2. *Chem. Eng. J.* **497**, 154455 (2024).

98. Li, Z. Y., et al. Synergistic electrocatalysis of crystal facet and O-vacancy for enhancive urea synthesis from nitrate and CO_2. *Appl. Catal. B: Environ.* **338**, 122962 (2023).

99. Huang, X. M., et al. The tandem nitrate and CO_2 reduction for urea electrosynthesis: Role of surface N-intermediates in CO_2 capture and activation. *Angew. Chem. Int. Ed.* **63**, e202403980 (2024).

100. Wu, Y. J., et al. Trace Cu-induced low C–N coupling barrier on amorphous Co metallene boride for boosting electrochemical urea production. *Small* 2407679 (2024).

101. Leverett, J., et al. Tuning the coordination structure of Cu–N–C single atom catalysts for simultaneous electrochemical reduction of CO_2 and NO_3^- to urea. *Adv. Energy Mater.* **12**, 2201500 (2022).

102. Chen, K., et al. Urea electrosynthesis from nitrate and CO_2 on diatomic alloys. *Adv. Mater.* **36**, 2402160 (2024).

103. Song, X. N., et al. Urea synthesis via coelectrolysis of CO_2 and nitrate over heterostructured Cu–Bi catalysts. *J. Am. Chem. Soc.* **146**, 25813–25823 (2024).

104. Zhang, M.-D., Huang, J.-R., Liao, P.-Q. & Chen, X.-M. Utilisation of carbon dioxide and nitrate for urea electrosynthesis with a Cu-based metal-organic framework. *Chem. Commun.* **60**, 3669–3672 (2024).

105. Zhao, C., et al. Tailoring activation intermediates of CO_2 initiates C–N coupling for highly selective urea electrosynthesis. *J. Am. Chem. Soc.* **147**, 8871–8880 (2025).

106. Tao, Z. X., et al. Cascade electrocatalytic reduction of carbon dioxide and nitrate to ethylamine. *J. Energy Chem.* **65**, 367–370 (2022).

107. Wu, Y. S., et al. Direct electrosynthesis of methylamine from carbon dioxide and nitrate. *Nat. Sustain.* **4**, 725–730 (2021).

108. Ali, A., et al. Production of sodium bicarbonate with saline brine and CO_2 co-utilization: comparing modified Solvay approaches. *Crystals* **13**, 470 (2023).

109. Benyahia, F. Carbon dioxide mineralization using reject brine. United States Patent 9475000B2 (2016).

110. Dindi, A., Quang, D. V., Alnashef, I. & Abu-Zahra, M. R. M. A process for combined CO_2 utilization and treatment of desalination reject brine. *Desalination* **442**, 62–74 (2018).

111. Lim, C., et al. Effect of Na_2CO_3 on production of $NaHCO_3$ using desulfurized Na_2SO_4 waste. *Korean J. Chem. Eng.* **41**, 2163–2172 (2024).

112. Ewis, D., Alnouri, S. Y. & El-Naas, M. H. A techno-economic assessment of conventional and modified Solvay processes for CO_2 capture and reject brine desalination. *J. Clean. Prod.* **468**, 143087 (2024).

113. Wu, Y. F., et al. Soda ash production with low energy consumption using proton cycled membrane electrolysis. *Ind. Eng. Chem. Res.* **58**, 3450–3458 (2019).

114. Haynes, W. M. *CRC Handbook of Chemistry and Physics*, Ch. 97 (CRC Press, 2016).
