# Supplementary material for: Operando-electrified solvay process
Source: Nat Commun. 2025 Oct 14;16:9128. doi: 10.1038/s41467-025-63539-3 (PMC12521556; doi:10.1038/s41467-025-63539-3)
Supplement: Supplementary file 2 — Description of Additional Supplementary Files [file 41467_2025_63539_MOESM2_ESM.pdf]

Description of Additional Supplementary Files

Supplementary Data 1: The optimized structures of reaction intermediates for NO_3RR on GaOOH/rGO surface with and without CO_2
